# Health system responsiveness: a systematic evidence mapping review of the global literature

**DOI:** 10.1186/s12939-021-01447-w

**Published:** 2021-05-01

**Authors:** Gadija Khan, Nancy Kagwanja, Eleanor Whyle, Lucy Gilson, Sassy Molyneux, Nikki Schaay, Benjamin Tsofa, Edwine Barasa, Jill Olivier

**Affiliations:** 1 School of Public Health and Family Medicine, Health Policy and Systems Division, University of Cape Town, Cape Town, South Africa; 2 Kenya Medical Research Institute (KEMRI)-Wellcome-Trust Research Programme, Kilifi, Kenya; 3 Department of Global Health and Development, London School of Hygiene and Tropical Medicine, London, UK; 4 Nuffield Department of Medicine, Center for Tropical medicine and Global Health, University of Oxford, Oxford, UK; 5 University of the Western Cape, School of Public Health, Cape Town, South Africa

**Keywords:** Responsiveness, Health system, Accountability, Feedback loops, User experience, Evidence mapping

## Abstract

**Background:**

The World Health Organisation framed responsiveness, fair financing and equity as intrinsic goals of health systems. However, of the three, responsiveness received significantly less attention. Responsiveness is essential to strengthen systems’ functioning; provide equitable and accountable services; and to protect the rights of citizens. There is an urgency to make systems more responsive, but our understanding of responsiveness is limited. We therefore sought to map existing evidence on health system responsiveness.

**Methods:**

A mixed method systemized evidence mapping review was conducted. We searched PubMed, EbscoHost, and Google Scholar. Published and grey literature; conceptual and empirical publications; published between 2000 and 2020 and English language texts were included. We screened titles and abstracts of 1119 publications and 870 full texts.

**Results:**

Six hundred twenty-one publications were included in the review. Evidence mapping shows substantially more publications between 2011 and 2020 (*n* = 462/621) than earlier periods. Most of the publications were from Europe (*n* = 139), with more publications relating to High Income Countries (*n* = 241) than Low-to-Middle Income Countries (*n* = 217). Most were empirical studies (*n* = 424/621) utilized quantitative methodologies (*n* = 232), while qualitative (*n* = 127) and mixed methods (*n* = 63) were more rare. Thematic analysis revealed eight primary conceptualizations of ‘health system responsiveness’, which can be fitted into three dominant categorizations: 1) unidirectional user-service interface; 2) responsiveness as feedback loops between users and the health system; and 3) responsiveness as accountability between public and the system.

**Conclusions:**

This evidence map shows a substantial body of available literature on health system responsiveness, but also reveals evidential gaps requiring further development, including: a clear definition and body of theory of responsiveness; the implementation and effectiveness of feedback loops; the systems responses to this feedback; context-specific mechanism-implementation experiences, particularly, of LMIC and fragile-and conflict affected states; and responsiveness as it relates to health equity, minority and vulnerable populations. Theoretical development is required, we suggest separating ideas of services and systems responsiveness, applying a stronger systems lens in future work. Further agenda-setting and resourcing of bridging work on health system responsiveness is suggested.

**Supplementary Information:**

The online version contains supplementary material available at 10.1186/s12939-021-01447-w.

## Introduction

The World Health Report of 2000 (WHR2000), ‘Health systems: improving performance’ broke ground framing health systems performance and development around three intrinsic goals: good health, fairness of financial contributions, and responsiveness to the expectations of the population – stressing the importance of responsiveness in particular in reducing inequalities, and improving the situation of the worst-off [1, 2].

The potential and significance of a responsive health system is that it should provide inclusive, legitimate, participatory and accountable services, ensure the social rights of citizens, and draw attention to the needs of minority groups [3–5]. More broadly, it should support nation-building, state-legitimacy, public participation, and social cohesion [6–8]. A responsive health system is also said to contribute to other health system goals such as improved access and acceptability of services, and improved health-seeking behavior, and therefore ultimately contribute to improved population health [9, 10]. However, the WHR2000 also foregrounded a debate around health systems as a ‘social good’ (values-based health systems), arguing that improved health system responsiveness is a legitimate endeavor in and of itself, irrespective of whether it directly improves population health or not. As emphasized by Da Silva [11] in a related report: ‘The greater the responsiveness of the health system to the expectations of individuals’ regarding the non-health enhancing aspects of care the higher will be the level of welfare achieved, irrespective of its impact on health’ (p.2). Non-health enhancing aspects of care here may include dignity of patients, confidentiality of information, autonomy, prompt attention, quality of the amenities, choice of provider, provider-patient communication and access to social support networks (for in-patients).

Health system responsiveness is also thought to improve systems functioning, for example, improving information flow and feedback, and improving capacities for decision-making within the health system [12, 13]. Therefore, interventions towards health system responsiveness are thought to have a health system strengthening effect, for example by strengthening ‘feedback channels’ [13, 14]. We use the term ‘feedback channel’ to describe the varied ways relevant information and evidence about systems functionality is fed back from those being served by the system (public/patients/community) to those actors with strategic decision-making authority over systems functionality. Feedback is channeled via formal mechanisms intended to facilitate the flow of feedback, such as complaints processes, but also via informal channels such as social media, or relational networks (more below). Effective feedback strengthens system functionality by ensuring that those being served by the system (public/patient/community) have voice in decision-making about *their* health system, and decision-makers have enough and the right information to make informed strategic decisions [15, 16]. This feedback enhances the chances for an effective systemic response to public/patient/community experience and views.

There have been multiple calls for initiatives and interventions to support health system responsiveness. Some are more ‘short-route’ interventions, such as efforts to strengthen information systems, to legitimize complaints systems, to increase community participation and voice, and the introduction of varied accountability mechanisms [17–19]. There are also ‘long-route’ interventions, such as democratic elections to vote in different government leadership, or macro-level systems interventions responding to national surveys or datasets. The short-route interventions are more prevalent, and more widely reported (more below). The most common are the ‘shortest-route’ feedback interventions such as formal facility-focused mechanisms focused on gathering patients’ perspectives on the quality of care they received, usually administered at the point of service during or immediately after care, such as score/report cards, social audits, and e-grievance systems such as toll-free hotlines and web-based portals have been introduced [20–22]. Also increasingly common are interventions which initiate accountability mechanisms such as clinic committees, intersectoral health forums, and community monitoring, these being one step removed from direct patient feedback [23–29].

We recognize that there are many issues (e.g. sufficient resources, having qualified staff and appropriate structures and supports) that contribute to responsive health systems and that patient voice is only one a component. The public (the ‘population’) continue to experience a range of problems in both high income countries (HIC) and low to middle income countries (LMICs): from lack of service availability to limited access; poor quality of services to ethical infringements and rights violations; commercial exploitation to collusion and corruption; rigid bureaucratic norms; to inadequate measures or processes and rules for accountability [27, 30–32]. Patients often experience inappropriate provider behavior including disrespect, abuse and inattention, and outright denial of care, much of which never gets reported through formal channels or mechanisms [9, 33–36]. It has also been shown that many health system actors (such as providers or policy makers) display limited *receptivity* to concerns raised by patients and the broader public [13]. The public continue to struggle to engage with the system about their problems and to secure appropriate responses and remedies [7, 22, 28]. Access to feedback channels and, more importantly, the ability to leverage reaction or response to feedback, is often inequitable, determined by social and educational status and the social capital that can be mustered [28, 37, 38] – yet while responsiveness as a health system goal is intended to draw attention to the needs of the vulnerable, such inequity has received little attention [32, 39].

Therefore, while there is great potential for enhanced health system responsiveness to improve systems’ functioning, ensure minority or vulnerable groups have more voice, and even lead to improved health, there is little evidence of this potential being fully leveraged. Two decades after the WHR2000, there has been substantial research and intervention work aimed at the goals of good health and fair financing, but in comparison, astonishingly little on health system responsiveness [13, 14, 40, 41]. There are still major questions about *every* aspect of responsiveness: its framing (for example, is it the same as accountability?); theorization (should the focus be on patient or population expectations?); resulting measurement (do you just measure patient satisfaction?); and praxis (what is a responsive health system, and how do you intervene to make a system more responsive?). What evidence there is to date, has not been collated in any useful way that allows researchers and practitioners to engage fully on the issue or develop it further.

In response to this, we conducted a systematic evidence mapping review on health system responsiveness, with a global scope, but seeking specifically to support research on LMICs. The aim was to comprehensively and descriptively map the currently dispersed terrain of evidence relating to ‘health system responsiveness’, in order to understand the current state of knowledge and identify evidence gaps for further work. The review is framed by the question, *what evidence is there on health system responsiveness, how it is framed, theorized, and measured; and what empirical evidence exists of related interventions in health systems?*

## Method

This systematic evidence mapping review was conducted by a team from [blinded] and [blinded], conducted during 2017–2020, resulting in output articles such as this, as well as a comprehensive documentary database (the database continues to be updated beyond the review end-date). Systematic evidence mapping reviews are increasingly being performed to map diverse literature in public health and health policy and systems research (HPSR) and involves systematic synthesis, organisation and interpretation across a large body of evidence, using rigorous and replicable strategies [42–44]. The approach is commonly used to organise and make available literature, as well as to describe the breadth and depth of this literature, identify its main characteristics and its gaps for future research [43–47]. The main characteristics we sought to describe were the quantity of evidence (areas of saturation and gaps), design and focus of research, and patterns pertaining to the content of literature such as the dominant framing of responsiveness.

While this review approach includes assessment of relevance and quality (of the publication source), it does not set out to assess the rigor of findings within the included studies, nor seek to compare the outcomes or effectiveness of interventions described. This is a common characteristic of evidence mapping reviews, as the approach is designed to describe a large quantity of literature, rather than delve deeply into each included item [42]. The application of deeper analysis pertaining to more specific research questions is understood as a subsequent activity and output after the evidence mapping review is concluded. Thus, the focus on a broad scoping of the terrain results in reduced analytical depth, and a large number of items needing to undergo full-text review and be reported to readers (in this case, over 800 items underwent full-text review). During the review process, we regularly considered approaches to reduce included items. For example, one possibility would have been to exclude items relating to ‘accountability’ as they are reviewed elsewhere [7, 31, 48, 49]. However, too many-directly relevant items important to understanding health system responsiveness made this an unviable exclusion option. Another option would have been to limit to items relating only to LMIC-settings, but again this would have removed core items relating to the conceptualization of health system responsiveness. Therefore, while such limitations might have reduced the final cluster included, they would have undermined the main aim of the review: to evidence the full breadth of most relevant publications relating to health system responsiveness, across diverse disciplinary terrains, in order to fully describe what is known about health system responsiveness at this time, and also be a comprehensive resource for future work.

We followed recommended phases for evidence mapping synthesis reviews including: 1) determining the scope and question of the topic under review; 2) searching for and selecting evidence; 3) mapping and reporting the findings of existing research; and 4) identifying evidence gaps [45, 50]. In the first phase, we refined the scope of the main review by conducting an initial rapid scoping review, which provided the analytical frame for the systematic review extraction process. Items found through the scoping review were subsumed (and assessed again) in the larger systematic review phase. We also conducted HPSR topic-expert consultations in the first phase (*n* = 6, [blinded]) – including experts in responsiveness, governance and accountability, in order to clarify topic scope and foci [45]. They supported the identification of search terms, topic areas, and key publications (conceptual and empirical). During this phase we refined study eligibility criteria and data extraction items for the evidence mapping component.

Next, we conducted a qualitative systematized review, keeping records of all searches conducted. Searches were performed using three electronic databases namely: EbscoHost (which is inclusive of Academic Search Premier; AfricaWide; Health Source; PsyhcInfo; SocIndex; and Cinalhl), PubMed, and Google Scholar. The initial staged searches were conducted during June–September 2019. To be eligible for inclusion, a paper needed to include ‘responsiveness’ and ‘health system’ and their variations (see Supplementary files). Initial pilot searches further refined the search terms and identified exclusion clusters.

Additional literature was sourced through reference list searches, expert consultations, hand-searching through Google search results (first 100 items, of varied search term variations), and through online repositories such as the WHO and World Bank online repositories. These searches were conducted iteratively until saturation was reached and no new relevant materials, nor further topics were found [51].

All abstracts were screened and included if they met the following inclusion criteria: (1) peer or institutionally-reviewed; (2) provided conceptual or empirical information on: responsiveness, accountability (internal and external) or user feedback within a health system; (3) published in English; and (4) published between 2000 and 2019 (earlier relevant material was included if directly relevant, although few were found). This period of publication was motivated by the inception of the conceptualisation and measurement of responsiveness by the WHO in 2000. No geographical limits were set.

We excluded items that met the following exclusion criteria: (1) studies about physiological or biomedical responsiveness to medication or treatment program; (2) responsiveness as a psychometric property of data collection instruments; (3) responsiveness that was not related to health or the health sector; 4) studies on feedback between providers only (e.g. performance feedback); (5) studies that focused on patient-reported outcomes measures (PROMS, specifically focused on the clinical aspects of care); (6) items where full texts could not be sourced; and (7) items that did not provide substantial information on health system responsiveness in the full text, or used ‘responsiveness’ in a descriptive, non-specific manner.

We examined the titles and abstracts/summaries to identify relevant items for further full-text screening. Three reviewers compared the eligible full-text documents and resolved discrepancies through discussions and consensus. During the initial screening process, we categorized items broadly as ‘empirical’ or ‘conceptual’. During the full-text review phase, we also conducted a further quality assessment phase, in which quality of publication source was assessed for all items (for example, publication indexed, or publishing institution known), and empirical items were further checked for clarity relating to stated aims, methodology (and rigor relating to execution of this methodology), and substantiation of findings. From the remaining items, we then extracted descriptive data into an extraction sheet, including: year of publication, publication type, country, region coverage, country status (economic ranking), study design, populations/samples, contribution (empirical/conceptual), and underpinning ideas and framing of responsiveness (see Supplementary materials). Refresher searches were conducted (using the same search terms and processes) quarterly (Dec 2019, March 2020, July 2020, Oct 2020), to check for newly published literature.

Our analysis in the review can be considered mixed methods given that we performed quantitative analysis (descriptive statistics) as well as qualitative (thematic) analysis. More specifically, we generated frequencies tables to determine the bibliographic results of the of the body of evidence, used thematic analysis to identify existing conceptualizations and dominant categorizations of health systems responsiveness.

## Results

**Fig. 1:**
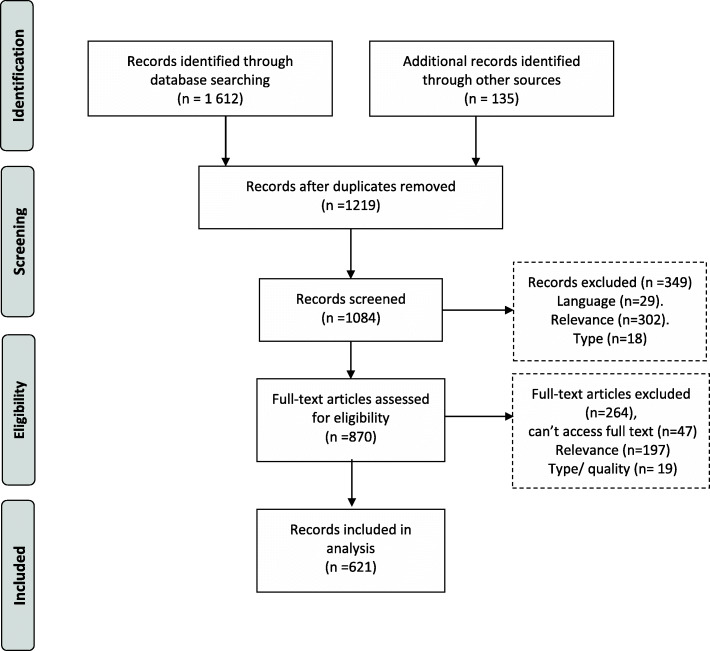
PRISMA flow diagram

The database search yielded a total of 1084 records, and an additional 134 records found by other means (Fig. 1). We collated the records and deleted duplicates, leaving 1219 records to be screened by title and abstract. After screening 870 items were included for full-text screening of potential relevance. The 2020 refresher searches resulted in 15 items being added. Ultimately, 621 items that were relevant to health system responsiveness were identified and included (see Supplementary materials for full listing of all 621 items).

### Bibliographic characteristics of the body of literature on health system responsiveness

**Fig. 2:**
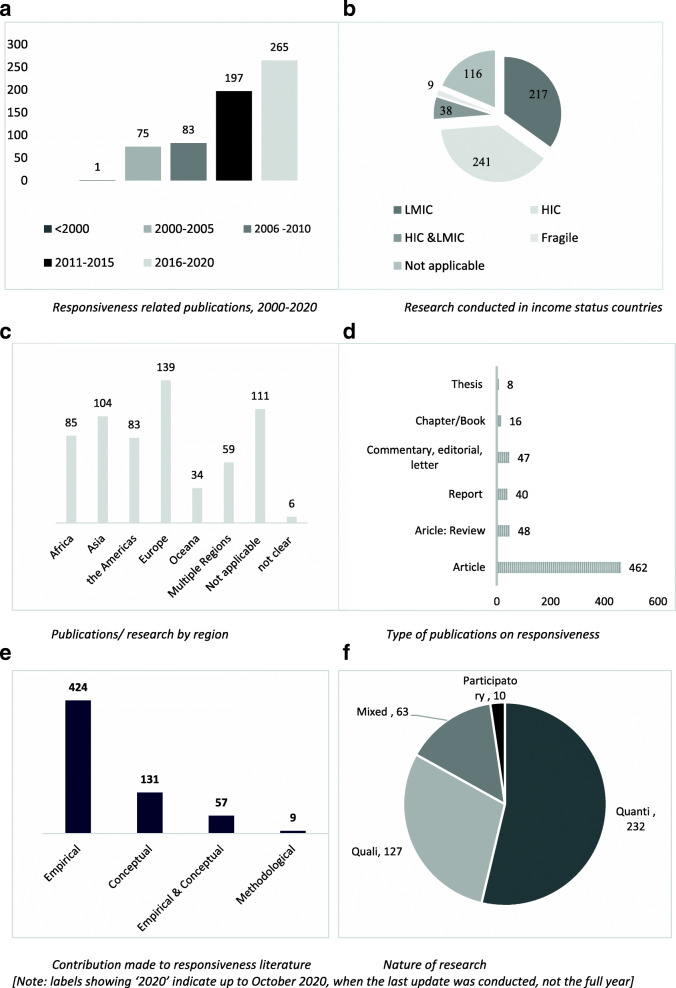
Consolidated graphics relating to publication rate, location and type

In the first results section we report on the included items (what we would term the evidence map), reviewing the collection of 621 items against consideration of publication rate, geographic location/focus, publication type, and empirical versus conceptual contribution. We have consolidated the graphics in Fig. 2 for ease of viewing.

In the last 20 years, there has been growth in interest and therefore publications on health system responsiveness. However, these are still very small numbers compared to the other goals such as health financing. After publication of the WHR2000, there was relatively limited interest in responsiveness (as indicated by publication numbers), until a decade later, around 2011 (see Fig. 2a). Slightly more items focus on HICs (241/621) versus LMICs (217/621), although more countries are classified as LMICs than HICs globally (Fig. 2b). Only nine (9/621) items on focus on fragile and conflict affected states (Fig. 2b) such as Afghanistan, the Democratic Republic of Congo and Sierra Leone. A large cluster focus on Europe (139/621), with a slightly smaller cluster on Asia (104/621), and the Americas (85/621) (See Fig. 2c). When disaggregated, the majority of the European publications relate to European-HICs (132/139), with only 8/139 relating to European-LMICs.

There were several types of included publications namely: peer-reviewed articles (empirical studies and reviews), chapters and books, theses, institutional reports (from multilateral or donor organizations such as the WHO, World Bank, United Nations (UN), and The President’s Emergency Plan for AIDS Relief (PEPFAR); civil society, research and academic institutions), and commentaries/editorials/letters. Most items were articles (462/621), including 48 review articles (mostly focused on accountability). Commentaries/editorials/letters made up the next largest grouping (47/621) and there were 40/621 institutional reports (Fig. 2d). With regard to the nature of contribution (shown in Fig. 2e). most items reported on empirical research (426/621); reporting quantitative data (232/426), with relatively fewer qualitative data-based studies (127/426) and mixed methods’ studies (63/426) (Fig. 2f). Conceptual items (131/621), reflected on issues relating to responsiveness. A few (57) papers presented the combination empirical-conceptual work (Fig. 2e).

### Underpinning ideas about health system responsiveness within the literature

The body of evidence contains varying definitions of health system responsiveness (Table 1). Authors seem to agree that health system responsiveness involves not only the system’s *ability* to respond, but also the actual response. For example, Joarder [55] defines responsiveness as the ‘ … social actions that providers do to meet the legitimate expectations of service seekers’ thus focusing on the tangible activities, processes and interaction between providers and service seekers (p.3). Lodenstein et al. [13] state that responsiveness is a culmination of system factors and processes such as ‘ … broader governance and health system context, features of the social accountability initiatives, motives and perceptions of providers at a particular point in time’ (p.2). Terms are used inconsistently across varied definitions, and there also seems to be little consensus in these definitions about who the system should be responsive to (some suggest service users, while others prescribe a broader focus towards citizens, communities and the public).

**Table 1:** Varying definitions for the concept of responsiveness

“Health system responsiveness indicates the ability of a health system to meet the population’s legitimate expectations regarding non-medical and non-financial aspects of the care process” [52]	
“Health systems responsiveness entails an actual experience of people’s interaction with their health system, which confirms or disconfirms their initial expectations” [14]	
“Responsiveness relates to a system’s ability to respond to the legitimate expectations of potential users about non-health enhancing aspects of care and in broad terms can be defined as the way in which individuals are treated and the environment in which they are treated, encompassing the notion” [53]	
“Responsiveness of human resources for health (HRH) is defined as the social actions that health providers do to meet the legitimate expectations of service seekers” [54]	
“Responsiveness of health providers to citizens’ concerns is thus the result of a combination of the broader governance and health system context, features of the social accountability initiative and motives and perceptions of providers at a particular point in time” [13]	

Within the 621 included publications, only eight explicitly provide a clear conceptualization or framing of health system responsiveness, and there are links between these eight. Table 2 provides an outline of these eight conceptualizations, describing the key features of each, where the conceptualization originates, what tools have developed from this, and an assessment of whether the conceptualization has had ‘traction’ within the broader included literature (that is, has it been taken up by other studies, tested empirically, or adapted further), as part of the ‘mapping’ of ideas about health system responsiveness.

**Table 2:** Explicit conceptualizations of health system responsiveness

Key features or components of conceptualizations	‘Impact’ of conceptualization in the literature
**WHO responsiveness framing: responsiveness as performance goal:** 2 categories (respect for patients, patient orientation); 8 domains: ▪ Dignity of a patient; confidentiality of information; autonomy; prompt attention; quality of the amenities; choice of provider; provider-patient communication; social support networks (for in-patients)	▪ Origin: Stems from WHR2000 [2] ▪ Linked tools: Data collection tool available to measure responsiveness ▪ Traction of idea: Some adaptations suggested for contexts and specific conditions [56, 57]
**WHO responsiveness framing: through a rights-based lens:** Adaptation of WHO framing, going further to recognizes that human rights/principles should enhance responsiveness through: ▪ A synergy of interrelated domains namely 1) protecting rights and maintaining health; 2) authority and accountability; and 3) cohesion	▪ Origin: Gostin et al. offer an adaptation, a conceptual lens to understand responsiveness [202] ▪ Linked tools: Does not provide a tool to measure responsiveness ▪ Traction of idea: No other related empirical work
**Health System Responsiveness Assessment Information System (HS-RAIS):** A Framework to measure responsiveness of the information system building block consisting of 10 components: ▪ Minimum datasets; data sources; data gathering; data analysis; feedback and dissemination; legislative needs; objectives of health system responsiveness assessment; repetition period; executive committee; stewardship	▪ Origin: Fazaeli et al. offer a framework developed after assessing responsiveness of Information Systems in Iran [1] ▪ Linked tools: Tool adapted from WHO tool, for evidence-based decision-making ▪ Traction of idea: No empirical studies found utilizing/testing this idea of responsiveness
**Provider responsiveness for HRH:** Conceptual framework to examine provider responsiveness (HRH lens). 5 domains: ▪ Friendliness; Respect; Informing and guiding; Gaining trust; Financial sensitivity	▪ Origin: Joarder proposes components of provider responsiveness [54], based on the WHO framing ▪ Linked tools: Provides a questionnaire to measure physicians’ responsiveness ▪ Traction of idea: The responsiveness tool developed was used to empirically compare the responsiveness of public and private physicians in rural Bangladesh.
**System-wide determinants of responsiveness:** Analytic framework to understand system-wide determinants of responsiveness consisting 4 components: ▪ Environment; Characteristics of population; Access/utilization; Responsiveness	▪ Origin: Robone et al. offer an adaptation based on WHR2000 [53] ▪ Linked tools: Does not provide a tool to measure responsiveness ▪ Traction of idea: The framework was developed to analyze determinants of responsiveness in 66 countries
**Responsiveness as social accountability:** Framing and tool to analyze key relationships of accountability and mechanisms that enhance service responsiveness, comprising 4 mechanism types: ▪ Delegation; Compact (service, policy stakeholders); Voice of citizens; Client power	▪ Origin: Garza used the World Bank model of relationships for accountability [58] ▪ Linked tools: Does not provide a tool to measure responsiveness ▪ Traction of idea: Model was empirically and analytically employed to analyze Mexico’s HS and three reforms
**Social accountability initiatives for health providers responsiveness** ▪ Provider responsiveness is an outcome of citizen engagement and oversight measures ▪ Responsiveness specifically defined as the actual changes/ improvements implemented at service/program level	▪ Origin: Lodenstein et al. develops this conceptualization out of a realist review, emphasizing context-specificity in regard to social accountability initiatives [13] ▪ Linked tools: Does not provide a tool to measure responsiveness ▪ Traction of idea: No empirical studies found utilizing/testing this idea of responsiveness
**Responsiveness as users’ experiences of HS interaction** ▪ Present factors that shape users’ expectations as well as the systems response. The experience of the interaction is central to responsiveness.	▪ Origin: Mirzoev and Kane offer this conceptualization out of a scoping review, which recognizes historical, political, cultural and socioeconomic context of people-system interaction [14] ▪ Linked tools: Does not provide a tool to measure responsiveness ▪ Traction of idea: No empirical studies found utilizing/testing this idea of responsiveness

We found no single widely accepted or clearly dominant framing of health system responsiveness among these eight, but unsurprisingly, the WHO-powered conceptualization first presented in the WHR2000 [54] shows the most traction, that is ‘the health system’s ability to meet the population’s legitimate expectations regarding non-health aspects of their interactions with the system’ (p.1). Responsiveness in this earlier WHR2000 framing comprises two main categories (respect for persons and patient orientation), with eight domains, namely: dignity of patients, confidentiality of information, autonomy, prompt attention, quality of the amenities, choice of provider, provider-patient communication and access to social support networks (for in-patients) [52]. There are now several variations of this idea – and four of the eight framings in Table 2 are self-declared adaptations of the WHR2000 conceptualization. Two of the four, offer conceptual frameworks and measurement tools for improving responsiveness of a specific building block (human resources and data information systems), while the other two offer a rights-based lens and analytic tool to understand system-wide determinants of responsiveness. Five of the eight provide both a conceptualization, and a developed tool for measurement of responsiveness against that conceptualization – while the remaining three are purely conceptual offering a framework or lens to understand health system responsiveness. It is not always possible to trace the development of a particular conceptualization from publication to publication over the 20-year period, and instead there appears to be a more disjointed ‘picking’ of ideas from different eras/topics/contexts.

### Three dominant categorizations of health system responsiveness

Beyond these eight conceptualizations, the explicit or implicit framing of health system responsiveness across the 621 included studies can be organized into three interrelated dominant ‘categorizations’:

1. *The unidirectional user-service interface*: strongly influenced by the WHO framing, items in this categorization tend to assesses responsiveness as a (usually national scale) service performance and quality indicator, and the preferred method for measurement, via the WHO designed quantitative instrument, is an exit survey at point of care, or household survey of patient experiences.
2. *Responsiveness as feedback between users and the system*: in this related cluster, the focus is on modes of gathering feedback from patients and patient representatives (usually gathered before, during or after care), and sometimes shows how feedback is utilized for service improvements.
3. *Responsiveness as accountability*: which mainly reports on processes and structures that support accountability (often broader than the patient, for example, community accountability). Specific tools and mechanisms are suggested and assessed, thought to ensure that stakeholders (users, public, provider and system) are answerable and held accountable for their actions.

**Fig. 3:**
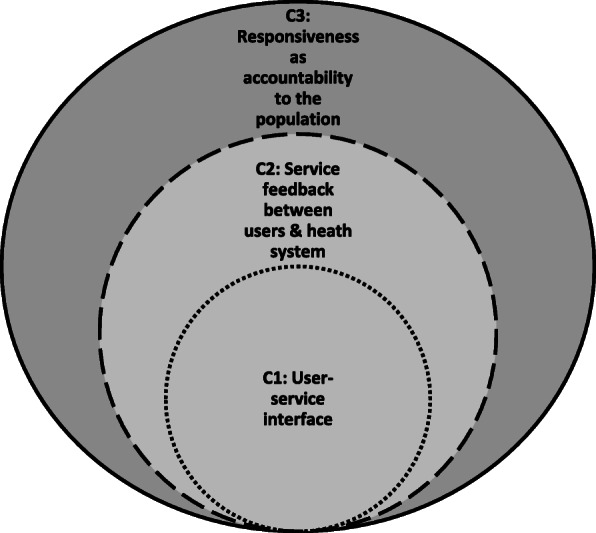
Relationship between the dominant categorizations

These categorizations are indicative, emerging from our review analytics, intended to give the reader a feel for the landscape (rather than to impose rigid classifications/typologies). The categorizations are therefore not totally distinct from each other, with obvious overlaps and relationships between them (see Fig. 3). For example, as illustrated in Table 3, the first two categorizations (user-service interface, and service feedback) focus on interactions at facility-level, and often gather feedback from users at point of exit, while the second and third categorizations (service feedback and accountability) include collecting feedback from ‘non-users’.

**Table 3:** Comparison of dominant categorizations of responsiveness in the literature

	Categorization 1: ‘Unidirectional user-service interface’	Categorization 2: ‘Service feedback between users and health system’	Categorization 3: ‘Accountability’
**Responsive to whom?**	Users/patients who have accessed health services	Various users (patients, patient representatives, intermediaries, those seeking access)	All: ‘citizens’, community, community representatives, patient advocates, civil society, the public, population
**How is health system responsiveness understood?**	As a quality checking and service improvement indicator Responsiveness assessments on particular building blocks	Processes to obtain feedback from users and respond to the feedback at a service level – placing user experiences as central to responsiveness	A responsive health system is a product of an accountable service and system Focus on formal (and sometimes informal) mechanisms to enhance accountabilty
**Setting of focus and flow of feedback and responsive**	In facility, at point of exit measured at the level of the individual, ussually uniderectional	Inside or outside or facility (before, during, after care)	Various, usually peripheral to the service (‘outside’ the facility)
**Representation in the literature**	25% (155/621) Decade most published: 2011-2020 Geographic spread: mostly LMICs (76/155), HICs (26/155) Empirical/Conceptual: mostly empirical (124/155)	40% (251/621) Decade most published: 2011-2020 Geographic spread: mostly HICs (118/251), LMICs (83/251) Empirical/Conceptual: Mostly empirical (179/251)	32% (196/621) Decade most published: 2011-2020 Geographic spread: mostly LMIC (89/196), HIC (53/196) Empirical/Conceptual: Mostly empirical (116/196)
**Exemplar study from this category**	Bramesfeld et al. provide an empirical measurement of the overall responsiveness of mental health services in Germany, comparing in and out-patients, using the WHO tool. Service responsiveness was assessed quantitively against 9 domains (attention, dignity, clear communication, autonomy, confidentiality, basic amenities, choice of provider, access to social support – and continuity) [59].	Serapioni and Duxbury showcase Mixed Advisory Committees (MAC) as a channel for obtaining, analysing and responding to the public within the Italian health care system. The advisory committtees included public and system actors. The MAC was a deliberative and participatory public consultation approach aimed at monitoring and assessing health-care quality from users’ perspectives [60]	Andrews et al. describe a participatory collaboration to design a web-based data collection and monitoring plan for health councils to use in New Mexico. The plan was co-developed (by users and systems actors) as a mechanism for accountability. Joint evaluations were conducted to outline processes and systems-level outcomes for county council development, planning, and community action [61].

Sorting literature into these categorizations is also complicated by different use of the same terms. For example, to avoid confusion, in the discussion below, we have clustered the varied terms used for ‘individuals’, grouping ‘patients’, ‘clients’, ‘users’ (from categorization 1 and 2) who are all effectively health service users; and grouping ‘citizens’ and ‘community’ (from categorization 3) who form the broader public and could also include users or potential users. Despite these overlaps and complexities, we find the proposed categorizations a useful way of understanding how health system responsiveness is framed and understood across the literature.

#### Description of categorization 1: ‘Unidirectional user-service interface’

For categorization 1, health system responsiveness is understood primarily as a performance and service quality indicator [53, 62]. This seems to originate in the WHR2000 on health system performance and was likely also influenced by the increased importance given to ‘patient-centered care’ in that decade, which includes emphasis on non-clinical aspect of care. The focus in this categorization is on gathering feedback from users about their experiences of their interacting with the health service [59–61, 63].

As depicted in Table 3, 25% (155/621) of the included items aligned with this categorization of health system responsiveness. Most papers were published in the last decade, and particularly in the last 5 years (2016 > 2020 = 57/155); more items focused on LMICs than HICs (LMIC = 76/155), and of these most focused on Asia (47/155), and then African LMICs (17/155). Items in this categorization were mostly empirical (124/155), with sub-clusters of responsiveness assessments of specific services (e.g. mental health, HIV, antenatal and reproductive services, ambulatory and chronic care), and of services for specific groups (e.g. older adults, people with mental health problems, physical disabilities, and migrants).

Measurement of responsiveness within this category was primarily quantitative, usually applying the WHO’s responsiveness survey instrument [11, 54, 64]. This instrument measures eight domains indicative of overall responsiveness level, and measures the distribution of responsiveness by groups (the inequality score) [11, 65, 66]. The importance of the domains of responsiveness varies between higher and lower income countries [11]. Measuring responsiveness in this way is primarily aimed at producing quantifiable indicators that denote overall health system performance [67]. With data collected at a national level through household surveys conducted as part of the WHO’s Multi-Country Survey Study on Health and Responsiveness 2000–2001 [68] and the World Health Surveys 2001–2004 [64]. These resulted in a global ‘ranking’ of countries by their overall level of responsiveness e.g. Italy, France and Spain were ranked as the top three most responsive systems in Europe [69]. Such surveys have not been repeated since, so there is no way of knowing whether countries have improved/regressed in relation to their national responsiveness assessment. More recent studies have applied the tool to measure responsiveness at the meso-level (organization/facility) or for specific services or programs [70–72]. There are also responsiveness assessments for the health system building blocks. Relating to the service delivery building block, Joarder, for example, offers a conceptual framework and measurement tool (a questionnaire) for service provider responsiveness which considers both the provider (service delivery context and practiced responsiveness) and client (demand) elements [55]. Fazaeli et al. also provide a framework and measurement tool for information and data system responsiveness [1].

Across items in this category, the WHO responsiveness tool has been validated and adaptations suggested – such as the addition of domains for education and information sharing [3, 36], effective care [73, 74], trust [73, 75], coordination and responsibility [62]. Several authors have argued that the WHO’s conceptualization and tools have inherent inadequacies [69, 76, 77]. For example, it is observed that the early WHO framing emerged out of key informant interviews with experts, but it is not clear who these experts were, and where they were drawing their experience from [69]. Others have observed that while this framing was intended to produce quantifiable indicators to allow for easier comparison across countries, services, and population groups [72], measuring the performance of a complex health system is not so easily done, and a single tool is unlikely to be adequate to assess a multi-dimensional compound measure such as responsiveness – or allow for fair comparison across vastly different health systems contexts [69].

#### Description of categorization 2: Feedback loops between users and health service providers

The publications in categorization 2 (Table 3), focus on a bi-directional flow of information between the health system and public, usually focused on health services specifically, on the grounds that, for example according to WHR2000 [54], ‘[the] effective flow of information between the health system and the population is a key element of responsiveness’ (p.3). This cluster comprises 40% of the included items (251/621). Most items were published in the last decade, and in the last 5 years (2016 > 2020 = 117/251); most are empirical (179/251); and most relate to HICs (118/251) rather than LMICs (83/251). There was a cluster of studies relating to Europe (72/251), then Africa (39/251), Asia (38/251), and the Americas (37/251).

In this cluster, authors focus on *actions taken* in response to user and public feedback, usually emphasizing the need for robust information systems, and shared decision-making in the development, provision and improvement of services to meet the expectations and needs of the public [15, 56, 78, 79]. It is also stressed that feedback from users is important to enhance transparency and accountability [15], so there is some overlap with the third categorization (accountability). Like the first categorization, this one relies mainly on gathering feedback relating to user experiences with the service – but has an additional focus on the action taken as a result, to ensure (usually individual) feedback is fed back to effect institutional change (usually service improvement). The types of actions most commonly described are analysis of feedback data to identify poor performance/service provision, and to improve safety and quality improvement procedures. Other types of action included the involvement of users in co-design or development of services. In this cluster, there is focus on user feedback at different timepoints (potential/current/previous users), and varied synonyms for feedback are present, such as patient-evaluations/expectations/preferences/experience/involvement [57, 80]. In this cluster, while it is apparent how feedback might improve the quality of a particular service, there are no robust (causal) explanations provided for how gathering feedback and resulting service improvement, leads to a more responsive health *system*. There are a few efforts in this direction, for example, the *Patient Feedback Response Framework* proposed by Sheard et al. [81] offers a way to assess systems change in response to feedback loops, proposing three stages: 1) *normative legitimacy*, or providers’ sense of moral obligation and receptivity to user feedback; 2) *structural legitimacy* emanates from providers’ perceived power within organizations (e.g. the perceived autonomy, authority and availability of resources to develop strategic plans in response to patient feedback); and 3), *organizational readiness to change*, a collective resolve to pursue the courses of action involved in change implementation [81].

Within this categorization, publications can be divided into four themes: 1) receptivity of systems actors, which includes the exploration of users’ and providers’ perspective regarding feedback loops such as complaint management processes, patient experience and user involvement in services [82–85]; 2) the empirical collection and analysis of feedback data [86–92]; 3) the utilization feedback to effect change including improved health outcomes, health worker behavioral skills to enhance community/public communication and relationship, [15, 79, 81, 85, 93, 94]; and 4) the direct involvement of users in the improvement of services [95–97]. Notably, all four themes included some level of action, focused on the systems response and not just merely gathering user feedback.

With regard to methodologies, feedback is usually collected at an individual (micro) level through self-reported instruments such as satisfaction-, quality-, or experience of care surveys [87, 92, 98–102], as well as analysis of complaint and feedback management procedures [103, 104], unstructured qualitative feedback and follow-ups [89, 105, 106], provider rating reviews [86, 90, 107]. Satisfaction surveys and feedback gathered via complaints processes are by far the most commonly reported of these.

#### Description of categorization 3: Responsiveness as accountability

In the third categorization, responsiveness is understood as a broader issue of accountability, not only to users, but to the broader public. According to Baharvand [108] ‘responsiveness in the public sector is called accountability. And needs a proper accountability system’ (p.1). Even if these studies assess micro/individual level interventions, for example, their framing of responsiveness is usually as a broader ‘social good’, and the assumption is that when accountability to the broader public (or community) is strengthened, the health system becomes more responsive [13, 108]. Here, health system responsiveness is framed as inextricably part of ‘social accountability’ [13, 14, 109], where responsiveness to the public’s needs is a consequence of the interaction of broader governance and health system contexts [13].

In the available evidence, 32% (196/621) of the items pertained to this category. Most of these were recently published in 2011–2020 (146/196), mostly in the last 5 years (2016 > 2020 = 85); most are empirical (116/196); with most assessments focused on specific services (e.g. reproductive health); and most relate to LMIC settings (89/196), with regional clusters focused on Africa (44/196) and Asia (38/196).

Within this category, there is variation in how authors frame accountability, but they can still be usefully divided into those addressing responsiveness as it relates to ‘internal accountability’ (within health system and at different levels) or ‘external accountability’ (between the health system and community or civil society) [31, 58, 110–113]. Most related to assessments of external accountability (122/196), rather than internal accountability (45/196). Those reporting on internal accountability processes tend to address institutional governance and oversight mechanisms/processes that address building blocks such as health financing [112–114], clinical and services [113, 115–117], provider and human resources [30, 118]. Enhanced internal accountability is understood to make the system more responsive, although there is general acknowledgement of the complexity of internal accountability as a result of interdependent relationships between health systems actors [58, 109, 119–121]. Brinkerhoff stresses that accountability involves two way relationships, where those in positions of power are obligated to provide information about and/ or justification for their actions to other actors [122, 123]. Examples of empirical assessments of internal accountability by Hamal et al. and the Human Rights Watch, show how accountability failures (i.e. lack of monitoring of policy implementation and health services such as the maternal death review processes in the Indian and South African public systems), have implications for maternal health outcomes and inequities [48, 124]. Studies focused on external accountability tend to look at feedback between ‘community’ and system and tend to focus on the depth and level of involvement of actors (passive or active). For example, evaluations of ‘citizen engagement’ where the public directly or indirectly hold politicians, providers, policy-makers accountable for their actions or performance [23, 125, 126]. A recent study that has gained traction is a realist review of accountability initiatives by Lodenstein et al. who argue that social accountability has two dimensions: citizen engagement, and citizen oversight and monitoring, and that when the context enables civic engagement, through internal (formal) accountability measures as well as civil society, and media (informal), it changes provider incentives which results in provider responsiveness [13]. They argue that civic engagement without oversight mechanisms will not result in responsiveness but rather a minimum degree of ‘receptivity’. Accountability, by nature, is largely relational and interestingly we recognize the role of reciprocity in mediating responsive relationships (and networks) between stakeholders (system, providers, beneficiaries).

Among the papers in this cluster, the preferred method to assess internal accountability is the measurement of performance and quality assurance indicators for various building blocks (e.g. quality of care standards, financial efficiency), usually quantitively measured and narrowly framed [110, 117]. With regard to external accountability, there are generally two sub-clusters: one focusing on the creation of spaces for user involvement and citizen engagement/decision-making [23, 110, 127–129], usually measuring performance using quantitative approaches. The second sub-cluster focuses on the degree and quality of engagement and participation [130–133], and these tend to apply mixed method approaches [12, 134, 135], described as necessary for the complexity involved in this assessment.

### Mechanisms (and their feedback loops) that potentially support health system responsiveness

Moving beyond the conceptualisations of responsiveness found in the literature – this review next considers what is presented as best practice to make a health system more responsive. Across the (621) included studies, reports of interventions intended to enhance health system responsiveness focus predominantly on the introduction/strengthening of a particular ‘mechanism’. There is generally much greater mention of mechanism type, functioning and implementation approach than how feedback gathered via these mechanisms is acted upon, or how the system responds; there is also more on how mechanisms affect specific services than how multiple mechanisms/feedback channels impact on *overall* system responsiveness (more below).

Specific mechanisms are considered within all three categorizations, although most frequently mentioned in the Category 3 (accountability) cluster. There, ‘mechanisms’ are understood to be governance tools that facilitate and enhance (internal) accountability within a health system, or (external) between health system and the public [12, 13, 136]. Similarly, the term ‘mechanism’ is used for tools/interventions/activities intended to enhance feedback and therefore responsiveness within the system or between the public and the system. Some mechanisms are formally mandated (e.g. in policy), initiated by the system and institutionalized; but it is important to note that there are also informal forms of feedback that are important for system responsiveness, but are not always specifically sought out (e.g. advocacy via civil society or complaints via social media) [12, 13]. In Table 4, we provide examples of common ways the connection between feedback, mechanism, and responsiveness are described – noting that terms such as ‘feedback’, ‘mechanism’, ‘process’, ‘initiative’, and ‘intervention’ are used interchangeably. These descriptions are predominantly located within the C3 framing (accountability), and slightly present in the C2 framing (feedback loops between users and health service providers); while generally missing from the C1 framing (unidirectional user-service interface).

**Table 4:** Examples of descriptions of connections between mechanisms, feedback, and responsiveness

“An ideal feedback process involves the gathering of feedback (the mechanism), and the communication of a response, which forms a feedback loop” [16]	
“… as a mechanism of accountability, social auditing enables views of stakeholders (such as communities and funders) to be considered in developing or revising organizational values and goals, and in designing indicators for assessing performance” [137]	
“Feedback mechanisms offer beneficiaries the opportunity to approach an organisation to ask questions and receive a response, increasing their understanding of the program, reducing potential tensions and potentially developing their trust in the organisation” [138]	
“… community scorecards are citizen-driven accountability measures that enhance citizens’ civic involvement and voices and complement conventional supply-side mechanisms of accountability, such as political checks and balances, accounting and auditing systems, administrative rules, and legal procedures” [139]	
“… community empowerment initiatives often target capacity to exercise oversight and to provide feedback to service providers” [124]	
“These diverse social accountability processes share three broad components as a part of their theory of change, namely information, collective action and official response” [140]	

Almost half of all included items (302/621) focus on responsiveness *mechanisms* (Table 5) – in particular formally mandated/institutionalized ones such as: community monitoring, complaint management procedures, satisfaction or quality of care surveys, incident reporting, intersectoral action/collaboration, health facility committees (HFCs) and hospital boards, medico-legal cases, ombudsman, patient charters, satisfaction−/experience−/quality of care surveys, social audits, and scorecard/report cards. Informal feedback is less prominent.

**Table 5:** Mechanisms that potentially support health system responsiveness (organized by publication prominence)

Mechanism / feedback	Total in sub-cluster	Underlying research themes within publications	Example of this mechanism
**Satisfaction surveys/ experience or quality of care surveys (formal)**	82/301 (27%)	Designing surveys/ tools to measure; Empirical measurement of indicators; Response from and within the system or interventions informed by this mechanism	Saadat et al. examines the relationship between a healthcare reform plan and patient satisfaction in hospitals in Iran [137]
**HFCs and hospital boards (formal)**	54/301 (18%)	Legitimizes HFCs in HS, roles and responsibilities; Describes implementation, functioning and processes of mechanism; Impact shown on health services, health outcomes, participation and accountability	Oguntunde et al. assess the effectiveness of HFC (as an intervention) to increase access and utilization of Maternal and Child services in Nigeria [139]
**Complaint management procedures (formal)**	41/301 (14%)	Complaint management process; complaint analysis across time or cross-sectional; Response from and within the system or interventions informed by this mechanism	Gurung et al. analyzed complaint management systems in primary health care in Nepal [18]
**Scorecard/Report Cards (formal)**	37/301 (12%)	Development of scorecard; Implementation and measuring effect of this mechanisms	Ho et al. document the implementation of community scorecards in two provinces of Democratic Republic of Congo [140]
**Incident reporting (formal)**	32/301 (11%)	Incident reporting practices and analysis; Interventions to improve incident reporting; Barriers and facilitators for incident reporting	Gallagher and Kupas, analyzed emergency medical services safety incidents reported on an anonymized web-based reporting system 2003–2010 [141]
**Community Monitoring (formal)**	22/301 (7%)	Technical/theoretical literature on accountability via community monitoring; Documents implementation, and evaluates impact of this mechanism	Shukla and Sinha documents CB monitoring implementation in India, highlighting effects on community mobilization and quality of care [129]
**Data systems (formal)**	18/301 (6%)	Patient views on data system items; Designing and test interfaces/tools to engage user involvement in systems development; Response from the system	Andrews et al. conducted participatory evaluation to create an online data collection and monitoring system for New Mexico’s Community Health Councils [132]
**Intersectoral collaboration (formal)**	18/302 (6%)	Technical or theoretical literature; Effects on system change	Janse van Rensburg et al. explore extent and nature of state/non-state mental health service collaboration in South Africa [142]
**Social media (informal)**	17/302 (6%)	Feedback on experiences of services; Enabling patient participation, monitoring and decision-making	Antheunis et al. examines patients’/professionals’ motives for using social media in health care and barriers and expectations for health-related social media use in the Netherlands [143]
**Social Audits (formal)**	9/302 (3%)	Technical or theoretical literature on accountability that include this mechanism; Implementation of mechanism	Schaaf et al. conduct a realist analysis on the implementation of Citizen Voice and Action program implemented in Zambia [144]
**Medico-Legal (formal)**	9/302 (3%)	The role of judiciaries in enforcing rights; Analysis of litigation cases	Biehl et al. analyzed lawsuits filed against the state in Brazil, affirming the heterogeneity of the judicialization phenomenon [145]
**Patient Charters (formal)**	8/302 (3%)	Implementation of this mechanism	Gurung et al. investigate level of awareness of a Charter and implementation factors in Nepal’s primary health care system [146]
**Citizen Juries (formal)**	5/302 (2%)	Decision-making and policy formation; Health research priorities	Chuengsatiansup et al. examine how citizens’ jury enhance public participation in the development long-term care policy for elders in Thailand [147]
**Ombud (formal)**	3/302 (1%)	Role of ombudsman in complaint management procedure	Gil analyses context of complaints and assessment of institutional violence towards older people by National Inspection Service in Portugal [148]
**Media (informal)**	2/302 (1%)	User views/ feedback interface and complaints; Health advocacy	Cullinan describes implementation of pilot study (OurHealth) on civic journalism in South Africa [149].
**Social protests (informal**^a^**)**	2/302 (1%)	Protests action by the public as feedback; Response from system	Sri et al. documents maternal death investigation as response to protest action in India [150]

^a^ We acknowledge that there may be protests that are mandated, however we are regarding social protests as an ‘informal mechanism’ given they generally do not request feedback, and also fall outside of what traditionally has been considered as an example of health policy and/or related legislation

In this cluster of 302 items relating to mechanisms (Table 5), most provide a general, usually conceptually-based, description of a particular formal mechanisms and their role in health systems functioning and strengthening [17, 133, 137–144]. There are also two smaller clusters of items, namely: 1) publications that report an *evaluation of a* mechanism; and 2) publications that *describe* the process of implementing a particular mechanism. The evaluative sub-cluster contains mainly empirical quantitative studies such as quasi-experimental, randomized-controlled or matched interventions designs (pre and post intervention) [145–147]. The effectiveness of mechanisms are commonly measured against the improvement of quality of care and coverage indicators [145, 148, 149]; health outcome indicators [147, 150] and indicators of (degree of) *voice/participation* [150–153]. While this review does not assess the validity of study findings, on the whole, there are significantly fewer reports of evaluated ‘success’ of mechanisms (in achieving intended outcomes, or showing improvement in responsiveness), than reports of mechanisms failing to achieve intended effects/outcomes/impact. For example, while HFCs are one of the most widely described mechanism type, significant challenges are reported, across all regions. Challenges include lack of awareness of HFCS, inadequate planning and monitoring of the functioning processes, power imbalances between communities and health system actors and low levels of political will [148].

The studies focusing on mechanism implementation mainly rely on mixed methodologies and qualitative designs (e.g. ethnographic, narrative and document analysis) [154–157], and offer insights relating to the operational processes and configuration of how these mechanisms function best, including specific activities such as training/meeting approaches and composition; implementation challenges or enablers [128, 135, 156, 158–160]; the roles of various systems actors in the functioning of these mechanisms, and the nature of relationships and networks (e.g. between state and non-state actors), as well as issues relating to leadership, representation, power dynamics, trust and communication [128, 159, 161–165]. It is also emphasized across this literature that mechanisms operate in a specific context, and their functioning cannot be separated from their context [162, 166]. Molyneux et al. [7] offer a framework that assesses factors influencing the functioning and impact of community accountability mechanism, including the design (details of the mechanisms and how it ought to operate, who should be involved), and process (how the mechanisms are actually functioning) [7].

Across the 302 items (Table 5), the mechanisms that receive the most attention are satisfaction surveys, quality of care surveys; HFCs and hospital boards; scorecards and complaint management systems are the more commonly reported mechanisms – suggesting they might be the most commonly implemented in practice. In relation to the publications considering satisfaction surveys, while most focused on empirically assessing user experiences [146, 167–169], there were a few that documented a reaction/response (actual or intentional) because they employed strategies to use satisfaction survey data to improve services [93, 170–172].

The cluster of publications relating to informal feedback and its (potential) impact on health system responsiveness was significantly smaller (21/301) than that examining formal feedback. The most commonly described of this form of feedback was via social media, the studies being primarily descriptive, often relating to the potential for user experiences (or complaints) to be fed through social media to service engagement [90, 168, 173–176]. For example, a case study on whether Twitter supports interpersonal communication and feedback to health services in the UK for people with mental disorders [177].

There are also a few other items relating to other forms of media (such as a description of civic journalism initiatives within five provinces in South Africa [178]; and social protest (such as the description of public protest in India attempting to hold systems actors accountable, and make demands for the system to be more responsive to needs of pregnant woman [179].

## Discussion

This review confirms there is continued and growing interest in health system responsiveness (evidenced by the rapid increase in recent publications), and its substantive relevance as a concept and area of focus - as a value, a key performance goal, and an important accountability and communication factor. As the WHR2000 argued, improved responsiveness is a legitimate endeavor in its own right, for protecting and enhancing the population’s basic human rights [54]. Therefore, as Askari [3] states ‘there is a growing need to increase the [health system’s responsiveness] as a key element of observance and fulfillment of justice’ (p.1). However, fair financing and equity still have more prominence and traction. For example, Wiysonge et al. reviewed the effects of financial arrangements for health systems specific to LMICs and found 7272 directly relevant items [180].

However, we also confirm that there are still major questions about every aspect of health system responsiveness: its framing (there are many), and theorization (there are few), resulting measurement (varied) and implementation practice (diverse). Without greater specificity, there is a risk that responsiveness remains a descriptive ideal, something mentioned in the introductory or conclusion sections of policies and articles – and the vital real-world application and effect remaining intangible.

Conceptual and definitional issues have received little attention, despite this being a standard pre-requisite for empirical research and intervention. Some of this ambiguity emerges as a result of the diversity of the field – and future work in this area should continue to consider context-specificity. However, researchers might also ‘check’ their framing against three initial questions: 1) what constitutes a response?; 2) at what level is response anticipated (provider or systemic)?; and 3) who is the response for (individual or public)? We do not see it as a task of this evidence mapping review to provide a ‘new’ definition for health system responsiveness. Instead, we would advocate for a broader and collective project of theoretical development, that emerges from context-specific realities, and that builds a dialectical bridge across the multiple interests and ideas described earlier.

The lack of coherent framing is important, as this means there is no main coherent idea or theory to test and develop further. This review shows that while authors might use the same term (responsiveness), there are vastly different interpretations lying under this use, drawing from varied applications/sources, rather than iteratively building on clustered ideas, or linking and learning from similar applications in different contexts. The varied clusters of work on health system responsiveness remains largely siloed from each other and often based on individual interests. This has had an impact on the theoretical development (lacking an iterative dialectic approach), as well as the empirical evidence-base – resulting in wildly diverse conceptualizations of what responsiveness is and how it should be measured, as well as conclusions about how to improve ‘it’.

Despite the order artificially imposed in this review, the evidential landscape remains largely ‘chaotic’ (an important finding in itself). The diversity of framing and focus, reflected in differing application of ideas and measurement approaches, makes it extremely challenging for researchers and practitioners seeking to enter this space. This might be a reason for its lesser traction than the other health system goals. Of course, diversity of ideas can encourage new thinking, and we are not encouraging conceptual ‘capture’ – but at this point, after two decades, this diversity appears to be more disabling than enabling.

Within that project, *all of the ideas* that underpin the compound concept that is ‘health system responsiveness’ would need to be interrogated and operationalised, as further research and implementation depends on achieving better clarity (see Table 6). For example, if the focus is on ‘systemic response to citizens’ legitimate expectations of the non-health enhancing aspects of services?’, then who is a citizen, who decides what a legitimate expectation is, and what is included/excluded as a non-health enhancing aspect requires interrogation. Furthermore, there are still significant questions about what a ‘response’ actually is – and how a ‘reaction’ might different from an ‘intentional response’, or how routinized responses might differ from, say, a public health emergency response.

**Table 6:** Theoretical questions for further engagement

- What is the main ideas underpinning ‘health system responsiveness’ not covered by other goals or indicators?	
- How is health system responsiveness related to and supported by the broader and universal principles of human rights and patient-centred care?	
- What are ‘legitimate expectations’? (who decides?)	
- Who (precisely) are the citizens (population/ individuals /patients) the system is being responsive too?	
- Are marginalized groups considered to be citizens with legitimate need? (e.g. migrants, those with mental health challenges, gender diverse individuals?)	
- Is the focus on service improvement, or systems strengthening?	
- What are ‘non-health/clinical aspects’?	
- What is systems receptivity and how do you measure it?	
- What are the variations of systemic ‘response’? (what is a response/reaction?) - What are the differences between ‘health services’ and ‘health systems’ responsiveness?	
- What would a ‘whole systems’ approach to improving responsiveness look like? (not necessarily national, but inclusive of different services, across building blocks etc)	

### Making a distinction between system and service responsiveness

As part of this call for theoretical development, we would also suggest it would be useful to develop a greater theoretical distinction between ‘health *system* responsiveness’ and ‘health *service* responsiveness’ (see Table 7). This review has shown that the majority of current items, might use the term ‘system’, but in fact, are primarily focused on the interaction between individual user/patient and the health *service* [2, 14, 181]. This explains why satisfaction surveys and complaints systems currently dominate the terrain. For example, the dominant depiction across all 621 items is of responsiveness as a specific service feedback loop, in which feedback (usually gathered at point of care) about a particular service is shared with that service, and it is about individual (micro)-level expectations, receptivity and feedback to that patient, and service-level reactions. However, this has been shown to be limited – and instead is strongly influenced by complex factors such as attitudes, societal values, and power dynamics among diverse actors [23, 24, 182]. We must question whether a more limited ‘services’ framing adequately captures the core ideas and systems thinking suggested of ‘health system responsiveness’ [3, 73, 77]. Few of the items reviewed here approach responsiveness from a ‘whole-of-systems’ perspective – a broader view of responsiveness, that takes into consideration the expectations of broader actors in the system (populations, not just users). Such a perspective is in line with the current trajectory within systems thinking and within HPSR [14, 183] – but would then presumably prioritize the assessment of responsiveness across multiple building blocks and focus on the interactions between blocks (instead of the single-block focus of much of the current empirical examples). Taking a systems view, receptivity might then be considered at a systemic level (e.g. organizational cultural orientation towards taking on feedback and adaptations, rather than individual decision-maker receptivity); feedback would more likely be understood as multiple streams of feedback from varied sources, via varied formal and informal channels; and reaction might be understood as a sustainable systems-wide reaction/response. In our view, part of the missing evidence map, is work on health system responsiveness that applies a systems-thinking approach, and acknowledges the complexity, multifaceted and interconnected relationships among the components in the health system [183]. This lens would assume health system responsiveness to be inclusive of ‘health service responsiveness’, but would extend more broadly, and require different framing and measurement approaches. For example, it would not be adequate to equate a survey of patient satisfaction at a particular point of care, with an assessment of *system* responsiveness.

**Table 7:** Conceptualising health system responsiveness as distinct from health service responsiveness

	‘Health service responsiveness’	‘Health system responsiveness’
*Focus*	Response of the health service to patient needs (patient-centered, individual)	Responsiveness of the whole system (public/private, all sectors), to all people in the system (people-centered, the public, citizens)
*Goal*	Improved quality of care, satisfaction of patient needs	A system that learns and adapts in response to the (sometimes multiple) needs of its people, towards the achievement of values such as equity and justice
*Reaction*	Can see feedback and immediate response on service	Reaction might to take longer (time-lag on HS change, HS more resistant to change than a specific service)
*Common mechanisms*	Surveys, score/report cards, patient records, patient autopsy, satisfaction/exit surveys, complaint boxes, hotlines, e-grievance systems, patient advocates	Social audits, information systems, clinic committees, intersectoral health forums, community monitoring, policy engagement, social media, social protest, community information systems
*Assessment*	Can be assessed in a linear fashion, considering single influences	Requires consideration of multiple factors and influences, including social and political context – complex and adaptive

Source: authors, derived from [41]

In addition to a project of theoretical development – a related project of assessment and research tool development is needed. This review shows there are few robust tools that comprehensively assess health system responsiveness as it is (variously) framed. Tools for assessing health system responsiveness, that encompass a system thinking approach, would still need to be developed. For example, the national scale of the survey tool that emerged from the WHR2000 does not necessarily enable researchers to assess the complex systemic aspects suggested in the framings and categorizations described above. To be fair, the WHR2000 tools were intended to produce quantifiable indicators to allow for easier comparison across countries [72]; but it is widely acknowledged that measuring the performance of a complex health system is not so easily done, and a single tool is unlikely to adequately assess a multi-dimensional compound measure such as responsiveness – or allow for fair comparison across vastly different health systems contexts. It was widely noted that the approach was too limited to encompass the broader complex ideas about responsiveness put forward in the WHR2000 [69]. Robone et al. noted that while this approached allowed you to see variations in reported levels of responsiveness across countries, the literature is sparse on the determinants of responsiveness, particularly of system-wide characteristics [184].

This review indicated other gaps relating to a systems perspective of responsiveness. For example, it is widely argued that systems functioning and change needs to be considered over time, suggesting that once-off surveys (such as the 2001 national assessments, or once-off service surveys focusing on a particular interaction) would not adequately assess whether systems are becoming more/less responsive over time, how systems are adapting to the changing needs of citizens, or how responsiveness relates to systems resilience (building positive adjustments to systems shocks over time). There were few assessments in this review that showed any type of cross-sectional assessment over time. Siloed and once-off service assessments do not show the fluidity of health systems, that change over time. Nor do they enable an understanding of varied levels of responsiveness within systems (or systems within systems), such as the variation between public and private sectors within the same national health system. For example, a for-profit health service might be *highly* responsive to the needs of a wealthy patient group, but would not necessarily contribute to a responsive national health *system* (where equity might require being *less* responsive to certain individual patient needs, [30]. The ‘systems side’ of health system responsiveness is seriously neglected and is the major theoretical gap – and development in this area would enable better bridging across the materials clustered in the three categories.

The case for health system responsiveness is also difficult to make because of missing empirical evidence (Table 8). For example, it is easy to see the geographic gaps, as HIC European systems tend to dominate. There are also several contexts in which responsiveness is an unknown – such as fragile and conflict affected states, where responsiveness might arguably be most essential. In building the case for responsiveness there would be value in mining the existing clusters for insights useful to other contexts – a research activity that has not been thoroughly accomplished. For example, the fact that certain approaches were developed for use in HICs, does not mean they would not bring valuable insight in LMIC settings. There are also opportunities for considering evidence across relatable contexts, or regionally. For example, it would be useful to mine the materials relating to particular mechanisms, exploring enable/disabling factors for successful implementation and mechanism functioning in comparable contexts.

**Table 8:** Empirical evidence gaps

- Development of more complex indicators, theoretical models and measurement tool	
- Empirically test existing frameworks to suit specific health system priorities	
- More context-specific work on systems responsiveness, in particular geographic gaps such as fragile and conflict affected states	
- More mining of existing clusters for useful evidence that can be theoretically generalised to relatable contexts (e.g. between LMIC and HIC contexts)	
- More work on health system responsiveness in fragile and conflict affected states (all aspects)	
- More work on health system responsiveness relating to minorities and vulnerable groups, equity	
- Empirical work on how responsiveness relates to health system strengthening (sustainable change over time)	
- Empirical work tracking ‘systems receptivity’ and ‘systems reactions’ to feedback	
- Empirical work on multiple forms and flows of feedback within a particular systems context	
- More empirical work on the longer-term systems response (not just on shorter-term reaction, or stopping at point of gathering feedback)	
- More outcomes evaluation of effectiveness of mechanisms	
- More cross-sectional work considering responsiveness over time	
- More consideration of informal feedback, and interaction of informal feedback and feedback gathered via formal mechanisms	
- More consideration of wider range of actors in responsiveness – including civil society	
- More empirical research showing application of a ‘systems’ lens	

Beyond geography, another major gap of the current literature is population. Although minorities and vulnerable groups are at the centre of the very idea of responsiveness, this review showed how rarely such groups are addressed - and this is a significant gap. All of these require more exploration, as does the broader connection between responsiveness and equity as it relates to a population as a whole.

In the current evidence-base, many items focus on whether mechanisms are currently present and functioning or not. It also tends to evidence challenges facing mechanism implementation more often than enablers and success stories. There are only a few examples of short-term and quite limited successes – and even fewer examples available of fully functioning mechanisms, implemented and operating as intended, consistently ensuring citizen voice and feedback gets taken up by the system, and resulting in systemic response, over sustained periods of time. There are opportunities to mine and repurpose existing data on mechanisms for new uses. For example, satisfaction surveys are widely applied in multiple countries, usually at a national scale, but there are few examples of such being leveraged to support work on systems responsiveness (it might not tell the whole story, but might provide an important piece of the puzzle). There are opportunities for comparing differences in mechanism performance in different contexts, and for integration of information about multiple mechanisms in the same system, to gain a more complex map of feedback.

Researchers (especially those in C3) have sought to take broader forms of feedback into consideration – for example applying rights-based approaches, taking broader ‘users’ into account. There is a large body of work on the types of feedback and empirical evaluations that demonstrate that feedback loops contribute quality improvement or systems changes. However, there is limited published literature that synthesizes the ‘how’ or the factors that hinder and enable feedback loops to facilitate a systems response. Further, of the included (621) studies tend to focus on the ‘gathering feedback’ and fewer on responsiveness as ‘the way the system responds or reacts to that feedback’. While there is evidence of feedback loops being in place and functional, what is not as clear is whether/how such feedback engenders response to citizen expectations. There is also as yet no robust explanations provided for how feedback leads towards a more responsive health system. Responsiveness is rarely framed as the actual (systems strengthening) changes made in the health system to address/respond to issues identified.

While there is merit to further work determining the effectiveness of mechanisms, there has been a call to move towards exploring the more nuanced aspects of their functioning in context, and in consideration of accountability relationships [185]. Further, better approaches for considering multiple actors influencing these mechanism(s) are needed. That is, the evidence indicates that the varied composition of different actors (state, health providers and staff, civil society or groups of individuals from communities) shape these mechanisms. (Civil society actors in particular are poorly evidenced/represented in the current research). What is less apparent is how varied actors facilitate mechanism processes at different levels of the system. Within the implementation of mechanisms, power and positionality are thought to be fundamental aspects, specifically to influence legitimacy and promote voice, as people hold various levels of power to act and make decisions and as a result of power imbalances may become more pronounced in certain mechanisms.

Little is known about how informal feedback relates to formal mechanisms, or how either/both influence decision-making, or leverage the system to respond. The framing of responsiveness as accountability (more common in C3), lends itself more easily to take informal feedback into account – and generally relates more easily to a systems perspective. For example, pushing beyond the user-provider interaction and includes the public and other actors in the system to hold each other accountable. Another gap is further consideration of ‘multi-level governance’ as it relates to responsiveness – for example, generating perspectives of mechanisms and interactions inclusive of individual, collective and government actions and decisions [186], allowing for a detailed exploration and analysis interactions of influences, arrangements and configurations within and between mechanisms. However, in general, the current literature is imbalanced towards particular actors (mainly users and service providers), and towards individual formal mechanisms (rather than multiple mechanisms, and varied forms of feedback) – and suggests a bias towards understanding feedback gained via formally instituted mechanisms [185]. It is our perspective that a campaign started via social media, or a community that burns down a clinic in a desperate LMIC setting, might also be considered a form of feedback relevant to system responsiveness – and hypothesize further that those without voice might provide feedback more frequently via informal channels [176, 178].

## Conclusion

The substantive relevance of having responsive health systems has been convincingly argued – but the evidencing of this claims is not yet fully developed. This leaves health system responsiveness as a ‘nice to have’ or an ideal – rather than a concrete performance goal requiring routine monitoring, attention and resourcing. Although health system responsiveness is understood to be important in many ways – for example, ensuring the social rights of citizens, drawing attention to minority groups, supporting social cohesion, improving population health, improving systems functioning, and ultimately having a health system strengthening effect - at this time, these ideas remain untested hypotheses. There is very little literature providing evidence for these claims or showing how a more responsive health system is a stronger health system.

This is one example of why there is still significant work to be done on health system responsiveness. In comparison with the other goals, there appears to have been a lack of prioritization and resourcing of work on responsiveness in the research, policy, and research/intervention arenas [58, 187, 188]. Currently, there are no distinct research interest or ‘sub-field’ teams working within the health system responsiveness terrain; no specific international networks or platforms focusing on it either (in comparison with other goals or topics). Further research agenda-setting work is required, as is resource mobilization to support it. There is an urgent need for synthesis of existing ideas, development of new ideas, and ultimately of ‘bridging work’ across existing evidence. As this review shows, such initiatives would not need to start from scratch.

There is major work to be done, for researchers and practitioners. For researchers, improved theoretical development needs to lead to improved (more complex, and more suited to purpose) measures and tools – which need to be tested and extended in real world health systems. Better measurement tools (adequate for assessing this complex concept) should result in measurable improvements that can be pragmatically (and routinely) pursued by practitioners. For practitioners, if responsiveness is to move from being a ‘nice to have’ ideal, to a systems performance goal, then it needs to be taken more seriously, and more routinely monitored and considered. Ultimately, the question that remains is: whose responsibility is it, to ensure our health systems become more responsive? The answer might be as simple and as complex as ‘everyone’.

## Supplementary Information

**Additional file 1.** Search terms for review.

**Additional file 2.** Data extraction table for the 621 publications included in the review and analysis.

## Data Availability

All data generated or analysed during this study are included in this published article [and its supplementary files listed above].

## Abbreviations

CB: Community-based
HFC: Health facility committee
HIC: High income country
HPSR: Health policy and systems research
HS: Health system
LMIC: Low to middle income country
PEPFAR: The President’s Emergency Plan for AIDS Relief
PROMS: Patient-reported outcomes measures
QoC: Quality of care
UN: The United Nations
WHO: World Health Organization
WHR: World Health Report
WHR2000: World Health Report of 2000
