# Supplementary material for: Health system responsiveness: a systematic evidence mapping review of the global literature
Source: Int J Equity Health. 2021 May 1;20:112. doi: 10.1186/s12939-021-01447-w (PMC8088654; doi:10.1186/s12939-021-01447-w)
Supplement: Supplementary file 1 — Additional file 1. Search terms for review. [file 12939_2021_1447_MOESM1_ESM.docx]

**Final search term and variations**

| **Main term** | **Variations of the term searched** |
| --- | --- |
| Responsiveness | accountability; patient feedback; feedback mechanism; complaints; feedback; health facility committees; incident reporting; scorecard; community monitoring; protests; social media; media; public participation; and citizen participation. |
| Health system | public health; primary health; healthcare; health service, hospital, clinic |
