# Supplementary material for: Health system responsiveness: a systematic evidence mapping review of the global literature
Source: Int J Equity Health. 2021 May 1;20:112. doi: 10.1186/s12939-021-01447-w (PMC8088654; doi:10.1186/s12939-021-01447-w)
Supplement: Supplementary file 2 — Additional file 2. Data extraction table for the 621 publications included in the review and analysis. [file 12939_2021_1447_MOESM2_ESM.docx]

| **Data extraction table of the 621 publications included in the review** | | | | | | |
| --- | --- | --- | --- | --- | --- | --- |
| **Paper reference** | **Year** | **Country** | **Region** | **Income status of**  **country** | **Contribution towards responsiveness literature** | **Ideas about responsiveness** |
| Abbott (2012) | 2012 | Canada | America | HIC | Conceptual | Call for improved internal accountability -describes the role of the Health Council of Canada |
| Abelson et al. (2007) | 2012 | Canada | America | HIC | Empirical | Empirical assessment of public participation method 5 Canadian states (between 2001 & 2004) |
| Ackerman (2005) | 2005 | N/A | N/A | N/A | Conceptual | A conceptual discussion about social accountability (specifically on decentralisation and public sector reform) |
| Adams, Maben, & Robert (2018) | 2018 | England | Europe | HIC | Empirical | Empirical assessment on how providers make sense of patient complaints |
| Adeleye & Ofili (2010) | 2010 | N/A | N/A | N/A | Conceptual | Conceptual argument for need for intersectoral collaboration to improve the responsiveness and performance of PHC in developing countries |
| Adesanya et al. (2012) | 2012 | Nigeria | Africa | LMIC | Empirical | Measured responsiveness (WHO) in private and public sectors and comparing the two Nigerian hospitals |
| Ahmadi Kashkoli, Zarei, & Daneshkohan (2017) | 2017 | Iran | Asia | LMIC | Empirical | Investigated the effect of overall hospital responsiveness (WHO eight dimensions) and patient satisfaction |
| Aiken et al. (2012) | 2012 | Multi-Country | Multiple Regions | HIC | Empirical | Measured patient satisfaction along with patient safety and quality of hospital care in 12 countries in Europe and the United States |
| Al Dweik, Stacey, Kohen, & Yaya ( 2017) | 2017 | Multi-Country | Multiple Regions | Not clear | Conceptual | To determine the influencing factors (motives and barriers) of consumers reporting of adverse drug reactions |
| Alavi, Forouzan, et al. (2018) | 2018 | Iran | Asia | LMIC | Empirical | Assessed the overall responsiveness of rehabilitation centres for people with physical disabilities Tehran |
| Alavi, Khodaie Ardakani, et al. (2018) | 2018 | Iran | Asia | LMIC | Empirical | Investigated the sociodemographic inequality in responsiveness (WHO) in rehabilitation centres |
| Alavi et al. (2019) | 2019 | Iran | Asia | LMIC | Empirical | Investigated predictors of poor responsiveness in rehabilitation centres |
| Alhafaji, Frederiks, & Legemaate (2011) | 2011 | Netherlands | Europe | HIC | Empirical & conceptual | An analysis of the concurrence of complaints handling procedures in Dutch health system |
| Ali, Nikoloski, & Reka (2015) | 2013 | N/A | N/A | N/A | Empirical | Measured patient satisfaction with and overall responsiveness (WHO) of services in Qatar |
| Anderson (2019) | 2019 | Multi-Country | Multiple Regions | BOTH | Conceptual | A theoretical paper on the role of communication processes in complaints management |
| Anderson, Blenkinsopp, & Armstrong (2004) | 2004 | Multi-Country | Multiple Regions | BOTH | Empirical & conceptual | Reviewed literature on feedback from users on their perceptions and experiences of community pharmacy services |
| Anderson, Shepherd, & Salisbury (2006) | 2006 | Multi-Country | Europe | HIC | Empirical | Explored the process of public involvement in planning primary health care in the UK |
| Andersson, Matthis, Paredes, & Ngxowa (2004) | 2004 | South Africa | Africa | LMIC | Empirical | Presents the findings of a social audit (formal accountability mechanism) conducted in South Africa |
| Andrews, Sánchez, Carrillo, Allen-Ananins, & Cruz (2014) | 2014 | New Mexico | America | LMIC | Empirical | Described a participatory evaluation of an online data collection and monitoring system for New Mexico’s Community Health Councils |
| Antheunis, Tates, & Nieboer (2013) | 2013 | Netherlands | Europe | HIC | Empirical & conceptual | Reviewed literature on patients’ and health professionals’ use of social media in health |
| Askari et al. (2016) | 2016 | N/A | N/A | N/A | Empirical | Validated and adapted the WHO responsiveness instrument for Iran |
| Asprey et al. (2013) | 2013 | Multi-Country | Europe | HIC | Empirical | Investigated providers’ response to feedback on patients' experiences in the UK |
| Aston, Meagher-Stewart, Edwards, & Young (2009) | 2009 | Canada | America | HIC | Empirical | Explored nurses' perceptions of their role in fostering citizen participation in an eastern Canada |
| Atela, Bakibinga, Ettarh, Kyobutungi, & Cohn (2015) | 2015 | Kenya | Africa | LMIC | Empirical | Explored community members’ experiences of implementing a health facility charter in Kericho District Kenya |
| Atela (2013) | 2013 | Kenya | Africa | LMIC | Empirical | Analysed structures, process and outcomes of accountability in primary health care in rural Kenya |
| Atherton, Fleming, Williams, & Powell (2019) | 2019 | Multi-Country | Europe | HIC | Empirical | Explored providers experiences and self-reported behaviours towards online patient/ carer feedback |
| Awoke et al., (2017) | 2017 | N/A | N/A | N/A | Empirical | Examined how perceived health system responsiveness is associated with utilisation of public and private healthcare services |
| {Azhdari, 2020 #23159} | 2020 | Iran | Asia | LMIC | Empirical | Assessed the rate of responsiveness (WHO) across educational and non-educational health centers in Iran |
| Babu et al. (2019) | 2019 | India | Asia | LMIC | Empirical | Examined overall responsiveness (WHO) of state faculties toward poor internal migrant users in 13 Indian cities |
| Baharvand (2019) | 2019 | N/A | N/A | N/A | Empirical | Measured overall responsiveness (WHO) of hospitals towards in-patients in Khorramabad |
| Baim-Lance, Tietz, Lever, Swart, & Agins (2019) | 2019 | USA | America | HIC | Empirical | Explored patient participation in the delivery of healthcare services |
| Baines & Regan de Bere (2018) | 2018 | N/A | N/A | NOT CLEAR | Empirical & conceptual | Discussed patient and public involvement and desirable principles |
| Baldie, Guthrie, Entwistle, & Kroll (2018) | 2018 | N/A | N/A | NOT CLEAR | Empirical & conceptual | To identify factors that influence the utilisation and impact of patient’ feedback |
| Balestra, Dasgupta, Sandhya, & Mannell (2018) | 2018 | India | Asia | LMIC | Empirical | Document how community-based monitoring can foster political capabilities |
| Banfield et al. (2013) | 2013 | Australia | Oceana | HIC | Empirical | Explored information continuity in Australian primary care to assist decision makers in developing effective policy for coordination of care |
| Banka et al. (2015) | 2015 | USA | America | HIC | Empirical | Evaluated an intervention to improve patient satisfaction (i.e. a response to patient feedback) |
| Barg et al. (2017) | 2017 | Canada | America | HIC | Empirical | Examined public preferences for involvement in health policy decisions, across the contexts of medical research and healthcare. |
| Barker & Klopper (2007) | 2007 | South Africa | Africa | LMIC | Empirical | Describe the level of community participation in primary health care projects |
| Barry, Campbell, Asprey, & Richards (2016) | 2016 | England | Europe | HIC | Empirical | Examined how providers collect patient feedback data and utilize it for service improvement it to inform service provision. |
| Basu, Andrews, Kishore, Panjabi, & Stuckler (2012) | 2012 | N/A | N/A | N/A | Conceptual | Comparison of health system performance of private and public sector delivery in low- and middle-income countries. |
| Bauhoff, Hotchkiss, & Smith (2011) | 2011 | Georgia | America | LMIC | Empirical | Examine overall provider responsiveness (WHO) and satisfaction |
| Bauhoff, Tkacheva, Rabinovich, & Bogdan (2016) | 2016 | Tajikistan | Asia | LMIC | Empirical | Described the development of an accountability mechanism (citizen report card) |
| Bazemore, Phillips, & Miyoshi (2010) | 2010 | USA | America | HIC | Empirical | Examined stakeholders' perceptions of the use of a Geographic Information System (GIS) to improve primary health care |
| Bazzaz, Taghvaee, Salehi, Bakhtiari, & Shaye (2015) | 2015 | N/A | N/A | N/A | Empirical | Assess the health system’s responsiveness in academic and non-academic hospitals |
| Béhague, Kanhonou, Filippi, Lègonou, & Ronsmans (2008) | 2008 | Nigeria | Africa | LMIC | Empirical | Explored the factors (social and institutional processes) that influence obstetric patients to evaluate quality of healthcare |
| Behdjat, Rifkin, Tarin, & Sheikh (2009) | 2009 | Iran | Asia | LMIC | Empirical | Participatory (intervention) project on the involvement of Women Health Volunteers to improve services |
| Belela‐Anacleto & Pedreira (2016) | 2016 | N/A | N/A | N/A | Conceptual | A conceptual argument for improving accountability for patient safety |
| Bell et al. (2011) | 2011 | N/A | N/A | N/A | Conceptual | Conceptual discussion to state the case for 'collective accountability' to reduce medical error |
| Benn et al. (2009) | 2009 | Multi-Country | Europe | HIC | Empirical & conceptual | Identified forms of effective feedback through the incident reporting to improve the safety of front-line clinical work systems |
| Bennetts, Cross, & Bloomer (2011) | 2017 | Australia | Oceana | HIC | Empirical | Exploration of manager’s perspective understandings of consumer participation for mental health care |
| Berkin et al., (2016) | 2016 | USA | America | HIC | Empirical | Evaluation of scorecard implementation for postpartum patients |
| Berlan & Shiffman (2012) | 2012 | Multi-Country | Multiple Regions | LMIC | Empirical & conceptual | A review on factors that shape health provider accountability to consumers and identifying promising interventions to enhance responsiveness |
| Berner et al. (2014) | 2014 | USA | America | HIC | Empirical | Collected post care feedback following acute care visit (using an automated system, Interactive voice response systems (IVRS) |
| Berta, Laporte, & Wodchis (2014) | 2014 | Canada | America | HIC | Empirical | Discussion paper on approaches to accountability for long-term care homes |
| Biebel, Nicholson, Williams, & Hinden (2004) | 2004 | USA | America | HIC | Empirical | Examined factors associated with the responsiveness of state mental health authorities to parents with mental illness |
| Biehl, Socal, & Amon, 2016) | 2016 | Brazil | America | LMIC | Empirical | An analysis of medico-legal cases relating to access of care specifically the cost of medication |
| Biondo, King, Minhas, Fassbender, & Simon (2019) | 2019 | Canada | America | HIC | Empirical | Explored community group perspectives on public participation for advance care planning |
| Birkeland (2019) | 2019 | N/A | N/A | N/A | Conceptual | A viewpoint that complaints and adverse events is a form of user involvement and safety improvement |
| Birks, Aspinal, & Bloor (2018) | 2018 | Multi-Country | Europe | HIC | Empirical | A synthesis of evidence on drivers of litigation |
| Bjorkman & Svensson (2009) | 2009 | Uganda | Africa | LMIC | Empirical | Examined the long-run impact of community-based monitoring (accountability mechanism) intervention |
| Björkman Nyqvist, de Walque, & Svensson (2017) | 2017 | Uganda | Africa | LMIC | Empirical | Presented the findings of community-based monitoring (accountability mechanism) intervention (RCT) |
| Blake et al. (2016) | 2016 | Ghana | Africa | LMIC | Empirical | Implementation of scorecard (accountability mechanism) to improve for maternal and child health in Ghana |
| Blanchard, Godinot, Laureau, & Wodon (2007) | 2007 | Madagascar | Africa | LMIC | Conceptual | Discussion paper on participatory approaches for child health |
| Bleich, Ozaltin, & Murray (2009) | 2009 | Multi-Country | Europe | HIC | Empirical | Examined the association between factors that shape patient experiences and patient satisfaction |
| Blendon, Kim, & Benson (2001) | 2001 | N/A | N/A | N/A | Empirical | Comparison of the WHO performance assessments (including responsiveness) of 17 countries |
| Blendon, Schoen, DesRoches, Osborn, & Zapert (2003) | 2003 | Multi-Country | Multiple Regions | HIC | Empirical | Comparative survey of satisfaction of sicker adults in Australia, Canada, New Zealand, the United Kingdom, and the United States |
| Blignault, Aspinall, Reay, & Hyman (2017) | 2017 | Australia | Oceana | HIC | Empirical | Described the implementation of a joint consumer engagement strategy |
| Boiko et al. (2015) | 2015 | England | Europe | HIC | Empirical | Explored providers' views on the use patient experience surveys |
| Boivin, Dumez, Fancott, & L'Esperance (2018) | 2018 | Canada | America | HIC | Conceptual | Synthesis of literature that proposes an exosystemic approach to patient and citizen engagement in health |
| Bolam (2005) | 2005 | Multi-Country | Multiple Regions | NOT CLEAR | Conceptual | Argument for public participation into reduce inequalities in health |
| Bonino & Warner (2014) | 2014 | Multi-Country | Multiple Regions | FRAGILE/HUMANITARIAN | Conceptual | Review on humanitarian feedback mechanisms |
| Boogaard et al. (2018) | 2018 | Netherlands | Europe | HIC | Empirical | An evaluation (RCT) of the effects of two feedback interventions for end-of life outcomes for people with dementia |
| Boothroyd et al. (2017) | 2017 | USA | America | HIC | Conceptual | Describe the key functions, implementation and scaling functions of active involved community partnerships (AICP) |
| Bottacini, Scalia, & Goss (2017) | 2017 | Italy | Europe | HIC | Conceptual | Review on the state of patient and public participation in healthcare in Italy |
| Boucaud & Dorschner (2016) | 2016 | Canada | America | HIC | Empirical | Patient safety incident reporting in Canada |
| Bowyer et al. (2019) | 2019 | Multi-Country | Europe | HIC | Empirical | Described results of the AFTER study (Ascertaining Feedback To iWantGreatCarE questionnaire) in the development of a patient feedback questionnaire. |
| Boydell, McMullen, Cordero, Steyn, & Kiare (2019) | 2019 | N/A | N/A | N/A | Conceptual | Methodological aspects of researching social accountability in health system strengthening |
| Bradshaw (2008) | 2018 | England | Europe | HIC | Conceptual | Discussion on policies that seek to extend the role of health service users in England. |
| Bramesfeld et al. (2016) | 2016 | Multi-Country | Multiple Regions | BOTH | Empirical | Tested the applicability of WHO responsiveness instrument for mental health care users in Hannover, Germany |
| Bramesfeld, Wedegartner, Elgeti, & Bisson (2007) | 2007 | N/A | N/A | N/A | Empirical | Examined the performance and responsiveness of mental health care for inpatient and outpatient care in Germany |
| Bramesfeld & Stegbauer (2016) | 2016 | N/A | N/A | N/A | Conceptual | Describe the national mandatory Quality Improvement System (QIS) in Europe in 2014 includes WHO responsiveness indicators |
| Bramesfeld, Klippel, Seidel, Schwartz, & Dierks (2007) | 2007 | N/A | N/A | N/A | Empirical | Discussion on the assessment of performance of mental health service facilities and health service responsiveness |
| Bridges, Pope, & Braithwaite (2019) | 2019 | N/A | N/A | N/A | Conceptual | Argued for the importance of health system responsiveness to older people living with complex health needs. |
| Brinkerhoff (2003) | 2003 | N/A | N/A | N/A | Conceptual | Described three accountability-enhancing strategies: reducing abuse, assuring compliance with procedures and standards, and improving performance/learning. |
| Brinkerhoff (2004) | 2004 | N/A | N/A | N/A | Conceptual | Described three purposes for enhancing accountability: reducing abuse, assuring compliance with procedures and standards, and improving performance/learning |
| Brookes & Baker (2017) | 2017 | England | Europe | HIC | Empirical | Feedback analysis i.e. Key themes of positive and negative feedback in patients’ online feedback on NHS (National Health Service) for services in England. |
| Brown, Ford, Deighton, & Wolpert (2014) | 2014 | Multi-Country | Europe | HIC | Methodological | Developed a scoring protocol for converting users’ feedback into measurement in the UK |
| Brown et al. (2016) | 2016 | Rhode Island | America | HIC | Conceptual | Describe youth patient engagement initiatives for healthcare in Rhode Island |
| Bryant, Saxton, Madden, Bath, & Robinson (2008) | 2008 | Australia | Oceana | HIC | Empirical | Outlined the consumer participation arrangements in the planning and delivering drug treatment services |
| Bunton (2008) | 2008 | N/A | N/A | N/A | Conceptual | Viewpoint on public involvement in the planning and delivery of drug treatment services |
| Busse (2013) | 2013 | Multi-Country | Europe | HIC | Empirical | Conceptual: Explained the role of health system responsiveness in improving patient satisfaction |
| Busse, Valentine, S, Prasad, & Van Ginneken (2012) | 2012 | Multi-Country | Europe | HIC | Empirical | Conceptual discussion and argument on distinction between responsiveness, satisfaction and patient experience |
| Byrkjeflot, Neby, & Vrangbæk (2012) | 2012 | Multi-Country | Europe | HIC | Empirical | Analysis of the changes in accountability relationships in Norway and Denmark hospital systems in the context of 15 years of reform |
| Byskov et al., (2014) | 2014 | Multi-Country | Africa | LMIC | Empirical | Measured WHO responsiveness -results from key informants’ survey in 35 countries (WHO seminal research) |
|  |  |  |  |  |  |  |
| Callaghan & Wistow (2006) | 2006 | N/A | N/A | N/A | Conceptual | Theoretical discussion on decision making and power in the involvement of patients and public |
| Campbell, Narayanan, Burford, & Greco (2010) | 2010 | Multi-Country | Europe | HIC | Empirical | Development of a multi-source feedback (feedback on performance from colleagues and patients) CFEP360 tool in primary care in UK |
| {Canfield Hurd, 2015 #23150} | 2015 | Multi-Country | Multiple Regions | LMIC | Empirical | Reviews key global and regional programs or processes and describe intervention models that are currently utilized in India, Nigeria, and Uganda at national and sub-national levels |
| Cervia (2018) | 2018 | Italy | Europe | HIC | Empirical | Citizen engagement and the challenges of democratizing health |
| Chand Chauhan, Jacob Purty, & Singh (2015) | 2015 | India | Asia | LMIC | Empirical | Successful linkage for referred patients with TB and the improvement of incident reporting |
| Chao et al. (2017) | 2017 | N/A | N/A | N/A | Empirical | Assessed the healthcare system responsiveness and the factors that influence responsiveness, Jiangsu Province |
| Charles, Cross, & Griffiths (2017) | 2017 | Australia | Oceana | HIC | Empirical | Examined clinicians understanding of deaths reportable to the Coroner |
| Checkland, Marshall, & Harrison (2004) | 2004 | Not Clear | Europe | HIC | Conceptual | Argued for accountability, trust and confidence in medical practice and quality of care |
| Cheng, McGrath, Bridges, & Yiu (2015) | 2015 | Hong Kong | Asia | LMIC | Empirical | Adapted patient feedback questionnaire to improve provider-user communication |
| Cheraghi-Sohi & Bower (2008) | 2008 | N/A | N/A | NOT CLEAR | Empirical & conceptual | Measured the effect of patient feedback and provider training or a combination on the improvement of provider' interpersonal skills |
| Chew‐Graham (2016) | 2016 | N/A | N/A | N/A | Conceptual | Patient and public involvement and engagement and biases of reporting these engagements |
| Chimbindi, Barnighausen, & Newell (2014) | 2014 | South Africa | Africa | LMIC | Empirical | Measured patient satisfaction with HIV and TB treatment in a public programme |
| Chretien & Kind (2013) | 2013 | N/A | N/A | N/A | Conceptual | Discussed the role of social media as tool for clinical care |
| Christiaans-Dingelhoff et al. (2011) | 2011 | Netherlands | Europe | HIC | Empirical | Examined the concurrence of hospital adverse events reporting systems and patient records |
| Chuengsatiansup, Tengrang, Posayanonda, & Sihapark (2019) | 2019 | Thailand | Asia | LMIC | Empirical | Describe the citizen jury process and public participation in long-term care |
| Cleary, Molyneux, & Gilson (2013) | 2013 | Multi-Country | Multiple Regions | LMIC | Empirical & conceptual | Examined factors that influence the functioning of accountability mechanisms and relationships within the district health system |
| Cleary et al. (2014) | 2014 | South Africa | Africa | LMIC | Empirical | Described community participation at the district level, provides lesson learned |
| Cleopas et al. (2006) | 2006 | Switzerland | Europe | HIC | Empirical | Explored staff responsiveness and the influence on patients’ perceptions of a hypothetical medical error |
| Coates (2016) | 2016 | Australia | Oceana | HIC | Empirical | Evaluation of Youth Mental Health (YMH) service using perspective of discharged clients and their parents. |
| Colombo & Mosconi (2016) | 2016 | N/A | N/A | N/A | Conceptual | Citizens’ juries in the development of a screening policy |
| Condon (2017) | 2017 | Australia | Oceana | HIC | Conceptual | Argues for the necessity of obtaining feedback from service users |
| Contandriopoulos (2004) | 2004 | Canada | America | HIC | Empirical | Sociological perspective on public participation in health care |
| Contandriopoulos, Denis, & Langley (2004) | 2004 | Canada | America | HIC | Empirical | Conceptual discussion on public participation in health care |
| Cordery, Baskerville, & Porter (2010) | 2010 | New Zealand | Oceana | HIC | Empirical | Analysis of accountability relationships in primary health care since the introduction of reforms requiring non-profit primary health organisations (PHOs) to discharge holistic accountability. |
| Cordery (2008) | 2008 | New Zealand | Oceana | HIC | Empirical | Analysis of dimensions of accountability within New Zealand primary health care |
| Cornwall, Cordeiro, & Delgado (2006) | 2006 | Brazil | America | LMIC | Empirical | Description of initiatives by citizens to exact accountability from their municipal government north-eastern Brazil |
| Cornwall, Lucas, & Pasteur (2000) | 2000 | N/A | N/A | N/A | Conceptual | Described models for developing workable partnership models in the health sector |
| Coulter & Jenkinson (2005) | 2005 | N/A | N/A | N/A | Empirical | European patients’ views on health systems and healthcare providers' responsiveness |
| Covell et al. (2006) | 2006 | USA | America | HIC | Empirical | Measured the effect of two ways of obtaining user feedback on mental health services |
| Crawford et al. (2003) | 2003 | London | Europe | HIC | Empirical | Identified methods for involving service users in the planning and delivery of psychiatric services as well as enabling factors and challenges (Greater London, UK). |
| Crawford et al. (2002) | 2002 | N/A | N/A | N/A | Empirical & conceptual | Synthesis of literature on the involvement of patients in the planning and development of health care |
| Crofts et al. (2014) | 2014 | Zimbabwe | Africa | LMIC | Empirical | Adaptation and implementation of local maternity dashboards for clinical improvement |
| Cullinan (2013) | 2013 | South Africa | Africa | LMIC | Empirical | Described the Ourhealth civic journalism project to demonstrate media as an accountability and feedback mechanism |
| Danhoundo, Nasiri, & Wiktorowicz (2018) | 2018 | Multi-Country | Africa | LMIC | Empirical & conceptual | Identify the conditions that improve social accountability in sub-Saharan Africa |
| Danovitch & Kan (2017) | 2017 | USA | America | HIC | Empirical | The implementation of the Addiction Benefits Scorecard to promote accountability and user engagement in California, USA |
| Darby, Valentine, Murray, & de Silva (2000) | 2000 | Multi-Country | Multiple Regions | BOTH | Methodological | The report described the WHO strategy for measuring responsiveness (seminal work) |
| Dasgupt, Sandhya, Lobis, Verma, & Schaaf (2015) | 2015 | India | Asia | LMIC | Empirical | Described implementation of human rights-based project that uses technology to monitor online data regarding informal payments for maternal health care in two districts |
| Davidson, Perry, & Bell (2015) | 2015 | Multi-Country | Europe | HIC | Empirical & conceptual | A review of continuous patient feedback to improve psychological therapy in the United Kingdom. |
| Davies, Wright, Drake, & Bunting (2009) | 2009 |  | Europe | HIC | Empirical | Develop a service-user feedback system for adopted and foster children in mental health care |
| De Brun, Heavey, Waring, Dawson, & Scott (2017) | 2017 | Multi-Country | Europe | HIC | Empirical | Explored patient perspectives on providing feedback on safety experiences four hospitals in the UK. |
| de Freitas & Martin (2015) | 2015 | Netherlands | Europe | HIC | Empirical | A case study of public participation using the Participation Chain Model (PCM) conceptual framework in Dutch mental health advocacy project with Cape Verdeans migrants |
| de Kok (2019) | 2019 | Malawi | Africa | LMIC | Empirical | Explored loss in childbearing (miscarriages, perinatal deaths and maternal mortality) as a lens to understand accountability and health system functioning |
| de Lara & Guareschi (2016) | 2016 | Brazil | America | LMIC | Empirical | Discussed the involvement of the Forum for Defence of the Sistema Único de Saúde in the fight against health care privatization |
| de Silva & Valentine (2000) | 2000 | Multi-Country | Multiple Regions | BOTH | Conceptual | Presented the WHO framework for measuring responsiveness (discussion paper -seminal work) |
| de Silva (2000) | 2000 | Multi-Country | Multiple Regions | BOTH | Conceptual | Present the results of a key informants’ survey on health system responsiveness (WHO) in 35 countries |
| de Vos, Hamming, Cchua-Hendriks, & Marang-van de Mheen (2019) | 2019 | Netherlands | Europe | HIC | Empirical | Examined the relationships between adverse events and complaints admissions that co-occurred |
| De Vos et al. (2009) | 2009 | Multi-Country | Multiple Regions | LMIC | Conceptual | Presents chronological progression of academic discussions on participation, empowerment, and human rights as separate yet coordinated approaches to health. |
| DeCourcy, West, & Barron (2012) | 2012 | England | Europe | HIC | Empirical & conceptual | Determined the how feedback i.e. Patients’ evaluation of care (National Adult Inpatient Survey between 2002 to 2009) was used in the National Health Service. |
| Delgado-Gallego & Vazquez (2012) | 2012 | Colombia | America | LMIC | Empirical | Explored the changes in awareness and utilization of social accountability mechanisms of the Colombian health care system for a 10-year period (2000 and 2010) |
| Denis (2014) | 2014 | Canada | America | HIC | Conceptual | Conceptual discussion on accountability in healthcare organizations and systems commenting on the relationships of accountability |
| Desai, Ndukwu, & Mitchell (2015) | 2015 |  | America | HIC | Empirical | Explored the utilization of social media in the health care field i.e. User-provider communication |
| Dewi, Sudjana, & Oesman (2011) | 2011 | N/A | N/A | N/A | Empirical | Investigated the influence of quality of dental health care service on empathy and responsiveness in Government Hospital, Indonesia |
| Dickson, Riddell, Gilmour, & McCormack (2017) | 2017 |  | Europe | HIC | Empirical & conceptual | Realist synthesis on effective feedback mechanisms for older people in the context of acute care |
| Douangvichit & Liabsuetrakul (2012) | 2012 | Thailand | Asia | LMIC | Empirical | Assessed obstetric care health system responsiveness for hospital-based delivery care in Lao People’s Democratic Republic (Lao PDR) |
| Douglas et al. (2015) | 2015 | Not Clear | Not Clear | NOT CLEAR | Empirical | Explored the how feedback (i.e. Problem alerts) reported by youth clients and their caregivers impact clinician-reported session content |
| Dullie, Meland, Mildestvedt, Hetlevik, & Gjesdal (2018) | 2018 | Malawi | Africa | LMIC | Empirical | Investigated factors associated with patients’ experience of primary health care |
| Dyer (2004) | 2004 | Multi-Country | Europe | HIC | Empirical | Argument for public participation in health using a case study of public participation on Local Research Ethics Committees (LRECs’). |
| Ebrahim (2003) | 2003 | N/A | N/A | N/A | Conceptual | Explored the practices and processes of accountability in nongovernmental organizations (NGOs) including reports and disclosure statements, performance assessments and evaluations, participation, self-regulation, and social audits. |
| Ebrahimipour et al. (2013) | 2013 | N/A | N/A | N/A | Empirical | Examined the responsiveness (WHO) of general public and private hospitals in Mashhad, Iran |
| Edward et al. (2011) | 2011 | Afghanistan | Asia | FRAGILE/HUMANITARIAN | Empirical | Evaluation of health system performance using scorecard over 5-year period in Afghanistan |
| Edward et al. (2015) | 2015 | Afghanistan | Asia | FRAGILE/HUMANITARIAN | Empirical | Assessed feasibility of community scorecards (accountability mechanism) in fragile context (Afghanistan) |
| Eijkholt, Jankowski, & Fisher (2017) | 2017 |  | America | HIC | Conceptual | A discourse analysis of negative patient feedback in social media and blog forums |
| Ellins & Glasby (2016) | 2016 |  | America | HIC | Empirical | Participatory research to project which explored the hospital and discharge experiences of older people from minority ethnic communities in England |
| Emslie, Andrew, Entwistle, & Walker (2005) | 2005 | England | Europe | HIC | Empirical | Comparison of feedback (perspectives and method of feedback) between population-based sample and community-based public forum in Scotland |
| Entwistle et al. (2003) | 2003 | Scotland | Europe | HIC | Empirical | Exploration of public views on feedback methods relating to health services and National Health Service (NHS) |
| Enwere, Keating, & Weber (2014) | 2014 | N/A | N/A | N/A | Conceptual | Described the design and implementation of balance scorecard for a pharmacy department |
| Eriksson, Raharjo, & Gustavsson (2018) | 2018 | Sweden | Europe | HIC | Empirical | Gendered analysis of complaints at Swedish hospitals |
| Evans (2018) | 2018 | Zambia | Africa | LMIC | Empirical | A multi-level ethnographic study to benchmark maternal indicators at district and national levels (for increased accountability) in Zambia |
| Eyre & Gauld (2003) | 2003 | New Zealand | Oceana | HIC | Empirical | Described a case study of participation in a rural community health trust in Lawrence, New Zealand |
| Fabbri et al. (2019) | 2019 | India | Asia | LMIC | Empirical | Measured effect of report cards intervention on the improvement of maternal and child health (RCT) in Uttar Pradesh, India |
| Falisse, Meessen, Ndayishimiye, & Bossuyt (2012) | 2012 | Burundi | Africa | FRAGILE/HUMANITARIAN | Empirical | Analysis of two accountability mechanisms (elected health committees and community-based organizations) in a performance-based funding in Burundi |
| Farmakas et al. (2017) | 2017 | Cyprus | Europe | HIC | Empirical | Public engagement exercise for setting healthcare priorities i.e. Ranking public preferences of priorities in Cyprus |
| Farmer et al. (2018) | 2018 | Australia | Oceana | HIC | Empirical | Describe partnering activities (health services partnering with consumers), identified how health services recruit consumers, and the extent to which minority groups were included, in Australia |
| Farmer, Currie, Kenny, & Munoz (2015) | 2015 | Scotland | Europe | HIC | Empirical | Described the longer-term impacts of community participation in the design of rural health services in three Scottish communities. |
| Farrington, Burt, Boiko, Campbell, & Roland (2017) | 2017 | England | Europe | HIC | Empirical | Examined doctors’ attitudes towards patient feedback (experience of care survey data) in primary and secondary care, across two regions of England |
| {Fathi, 2019 #23157} | 2019 | N/A | N/A | LMIC | Empirical & conceptual | Argues for the measurement of responsiveness of rehabilitation centres for people with disabilities. |
| Fatima et al. (2017) | 2017 | Pakistan | Asia | LMIC | Empirical | Collected patient feedback (appraisal of quality of service) of tertiary care teaching hospitals in Pakistan |
| Fazaeli, Ahmadi, Rashidian, & Sadoughi (2014) | 2014 | N/A | N/A | N/A | Empirical | Designed a framework (HS-RAIS) to assess the responsiveness of information systems in health system for Iran |
| Fazaeli et al. (2016) | 2016 | N/A | N/A | N/A | Empirical | Measured importance of client orientation responsiveness domains (prompt attention, choice, quality of amenities, social support) in high- and low-income districts of Mashhad, Iran |
| Fekri, Macarayan, & Klazinga (2018) | 2018 | N/A | N/A | N/A | Empirical | Determined the use of health system performance assessment framework (WHO), specific focus on which domains and indicators that have been used by European region member states to measure performance. |
| Feruglio & Nisbett (2018) | 2018 | India | Asia | LMIC | Empirical | Challenges of institutionalization of community-level social accountability mechanisms in three districts of state of Odisha, India |
| Fiorentini, Ragazzi, & Robone (2015) | 2015 | Italy | Europe | HIC | Empirical | Examined the influence of patients’ characteristics (health status and pain) on their appraisal of health system’s responsiveness Emilia-Romagna, Italy |
| Fiorentini, Robone, & Verzulli (2018) | 2018 | Italy | Europe | HIC | Empirical | Examined hospital‐specialty characteristics on the influence on health system responsiveness in Emilia-Romagna, Italy |
| Fisher & Corrigan (2014) | 2014 | N/A | N/A | N/A | Conceptual | Viewpoint on enhancing accountability arrangements for community participation |
| Flores & Hernandez (2018) | 2018 | Guatemala | America | LMIC | Conceptual | A case study of indigenous citizen-led accountability initiatives in Guatemala that demonstrate intersectoral collaboration to dismantle power imbalances |
| Flores & Samuel (2019) | 2019 | N/A | N/A | N/A | Conceptual | Described the role of grassroot organisations (as social accountability) to reduce inequalities within countries. |
| Florin & Dixon (2004) | 2004 | Multi-Country | Europe | HIC | Conceptual | Described strategies for public involvement in health care, focus on NHS |
| Foran, Millar, & Dorstyn (2016) | 2016 | Australia | Oceana | HIC | Methodological | Developed and tested a patient satisfaction instrument for neuropsychological assessment services, in Australia |
| Forbat, Hubbard, & Kearney (2009) | 2009 | Scotland | Europe | HIC | Empirical | Describe models of patient involvement, drawing from a cross-sectional study of involvement (i.e. Five health boards) across Scotland |
| Forouzan (2015) | 2015 | Iran | Asia | LMIC | Empirical | Assessed applicability of WHO responsiveness instrument for mental healthcare users in Teheran, Iran |
| Forouzan, Ghazinour, Dejman, Rafeiey, & San Sebastian (2011) | 2011 | Iran | Asia | LMIC | Empirical | Adapted a Mental Health System Responsiveness Questionnaire (MHSRQ) based on the WHO instrument for Iran. |
| Forouzan et al. (2014) | 2014 | Iran | Asia | LMIC | Empirical | Assessed the psychometric properties of a Mental Health System Responsiveness Questionnaire in Tehran |
| Forouzan et al. (2016) | 2016 | Iran | Asia | LMIC | Empirical | Measured the responsiveness of mental health-care system of Tehran, using a newly developed Mental Health System Responsiveness Questionnaire |
| Foster, Piggott, Riley, & Beech (2016) | 2016 | Not Clear | Not Clear | NOT CLEAR | Empirical | Obtained feedback from youth (satisfaction survey) on England's ambulance service |
| Foster & Maillardet (2010) | 2010 | England | Europe | HIC | Empirical | Participatory research (primary care clinicians and patients) to introduce strategies for increasing referrals for pulmonary rehabilitation |
| Fox (2015) | 2015 | N/A | N/A | N/A | Conceptual | Described the evaluation of social accountability initiatives (synthesis of empirical work) |
| (Frambes et al. (2017) | 2017 | USA | America | HIC | Empirical | Evaluated the implementation of a scorecard to improve intervention fidelity (adhering to protocol) for a Caregiver-delivered Symptom Management Intervention |
| Fredriksson, Eriksson, & Tritter (2017) | 2017 | England | Europe | HIC | Empirical | Measured the willingness to be involved in individual treatment decisions and decisions about the organization and provision of local health and social care services, in Sweden and England |
| Fredriksson, Eriksson, & Tritter (2018) | 2018 | Sweden | Europe | HIC | Empirical | Perspectives of the impact of health participation activities in the Swedish population |
| Fredriksson & Tritter (2017) | 2017 | N/A | N/A | N/A | Conceptual | Argument for the distinction between patient and public involvement in healthcare decisions |
| Freedman & Schaaf (2013) | 2013 | N/A | N/A | N/A | Conceptual | Argument for accountability approaches that dismantles imbalanced power dynamics at the frontlines |
| Friele, Reitsma, & de Jong (2015) | 2015 | Netherlands | Europe | HIC | Empirical | Assessed the congruence of expectations of public and physicians regarding complaint handling processes in health care in the Netherlands |
| Frisancho (2013) | 2013 | Multi-Country | Multiple Regions | BOTH | Conceptual | Community monitoring case study of Peru using international Human Rights principles to strengthen the quality of care provided in health care services. |
| Frith, Young, & Woolfall (2014) | 2014 | N/A | N/A | N/A | Conceptual | A call for improved patient and public participation in health care |
| Frosch et al. (2011) | 2011 | USA | America | HIC | Conceptual | Discussed the role of shared decision making in policy and implementation activity on multiple fronts (e.g. State legislative activity, implementation in clinical practice, research funding) in United States |
| Gagnon (2002) | 2002 | Canada | Oceana | HIC | Empirical | Described how the Canadian health care system respond to the needs of newcomers (migrants) including access to health services |
| Rakhal Gaitonde, Muraleedharan, San Sebastian, & Hurtig (2019) | 2019 | India | Asia | LMIC | Empirical | Explored key stakeholders’ perceptions of accountability during the implementation of a flagship rural health strategy (National Rural Health Mission) in Tamil Nadu, India |
| Gaitonde, San Sebastian, Muraleedharan, & Hurtig (2017) | 2017 | India | Asia | LMIC | Empirical | Document the community action and participatory process of a flagship rural health strategy (National Rural Health Mission) in India |
| Gakidou, Murray, & Frenk (2000) | 2000 | Multi-Country | Multiple Regions | BOTH | Empirical | Measured the Member States' preferences of health system performance assessment indicators (WHO, development of performance framework). |
| Gal & Doron (2007) | 2007 | Israel | Asia | LMIC | Empirical | Determined the prevalence of informal complaints on health services among clients of Health Maintenance Organizations, in Israel |
| Gallagher & Kupas (2012) | 2012 | USA | America | HIC | Empirical | Analysis of state-wide incident reporting system to identify patterns and prevent future incidents in Pennsylvania, United States |
| Garg & Laskar (2010) | 2010 | India | Asia | LMIC | Empirical | Discussion on community-based monitoring as an important accountability mechanism and to improves success of health programs |
| Garrard & Narayan (2013) | 2013 |  | Europe | HIC | Empirical | Adapted a SERVQUAL (quality of services questionnaire) for antenatal clinic services in the UK. |
| Garza (2015) | 2015 | Mexico | America | LMIC | Conceptual | Analysis of Mexican public health services using the World Development (2004) accountability framework to propose three policies to improve voice and power |
| Gauld (2010) | 2010 | New Zealand | Oceana | HIC | Empirical | Examined whether elected health boards are effective mechanisms for public participation in health service in New Zealand |
| Gauld et al. (2011) | 2011 | New Zealand | Oceana | HIC | Empirical | Developed a national scorecard to assess New Zealand's health system performance, using routine data |
| Gaunt & Pawlikowska (2018) | 2018 | N/A | N/A | N/A | Conceptual | Viewpoint on physicians deliberately obtain feedback post-care |
| Geldsetzer, Haakenstad, James, & Atun (2018) | 2018 | Multi-Country | Multiple Regions | LMIC | Empirical | Assessed health system responsiveness (WHO) and quality of care in 5 LMIC countries China, Ghana, India, Mexico, Russia and South Africa |
| Geletta (2018) | 2018 | USA | America | HIC | Empirical | Explored the use social media data to measure patient satisfaction with medical care services. |
| Genovese et al. (2017) | 2017 | N/A | N/A | N/A | Conceptual | Argued for improved the quality of the system through enhanced individual and collective accountability |
| George (2003) | 2003 | N/A | N/A | N/A | Conceptual | Presented cases participatory processes for improving sexual and reproductive health service delivery |
| George (2009) | 2009 | India | Asia | LMIC | Empirical | Described managers views on two management functions (supervision and disciplinary action) that enhances internal accountability. |
| George, Subha Sri, & Ved (2016) | 2016 | India | Asia | LMIC | Empirical | Developed a framework that highlights mutually reinforcing dimensions of accountability in health systems along three counterbalancing axes |
| George, Erchick, Zubairu, Barau, & Wonodi (2016) | 2016 | Nigeria | Africa | LMIC | Empirical | Reviewed literature on community participation in health systems interventions in low- and middle-income countries (LMICs). |
| George, Scott, Mehra, & Sriram (2016) | 2016 | N/A | N/A | LMIC | Empirical & conceptual | Described community action initiatives to improve awareness, access, and accountability and identified elements to support governance and maternal health outcomes |
| Gigler & Bailur (2014) | 2014 | Multi-Country | Multiple Regions | LMIC& FRAGILE | Conceptual | A collection of approaches to utilize technology in enhancing accountability and closing the 'feedback loop' |
| Gil (2019) | 2019 | Portugal | Europe | HIC | Empirical | Analysis of quality procedures and complaints of mistreatment in nursing homes in Portugal |
| Giles, Reynolds, Heyhoe, & Armitage (2017) | 2017 | Not Clear | Europe | HIC | Empirical | Explored the feasibility of the development of a patient-led quality and safety feedback system within an existing electronic health record (EHR), known as Renal patient view (RPV). |
| Gill, Redden-Hoare, Dunning, Hughes, & Dolley (2015) | 2015 | Australia | Oceana | HIC | Empirical | Explored patient and staff opinions regarding point of service feedback |
| Gillespie & Reader (2018) | 2018 | England | Europe | HIC | Empirical | Analysis of health care complaints (across England) to improve quality and safety using the Healthcare Complaints Analysis Tool (NHS) |
| Ginter (2000) | 2000 | Slovakia | Europe | HIC | Empirical | Described the performance (including responsiveness) of the Slovak Republic using the World Health Report 2000 data |
| Glattstein-Young (2010) | 2010 | South Africa | Africa | LMIC | Empirical | Examined the relationship between participation (specifically health committees) and the right to health, offer best practice lessons from Western Cape |
| Golding (2014) | 2014 | N/A | N/A | N/A | Conceptual | Documented the role community health workers and Mhealth in strengthening community health systems and building capacities |
| Gooberman-Hill, Horwood, & Calnan (2008) | 2008 | England | Europe | HIC | Empirical | Examined the challenges and benefits of citizens’ juries in planning research priorities (including process, public engagement and outcome) |
| Goodman, Opwora, Kabare, & Molyneux (2011) | 2011 | Kenya | Africa | LMIC | Empirical | Explored the nature and depth of managerial engagement of Health facility committees and the influence on community accountability |
| Gostin, Hodge, Valentine, & Nygren-Krug (2003) | 2003 | Multi-Country | Multiple Regions | BOTH | Conceptual | Discussion on the human rights context for the recognition of WHO responsiveness domains in the provision of health services to the public |
| Grandvoinnet, Ghazia, & Shomikho (2015) | 2015 | Multi-Country | Multiple Regions | BOTH; FRAGILE | Conceptual | Argument for citizen engagement and describes drivers for social accountability |
| Grant, Sears, & Born (2008) | 2008 | Canada | America | HIC | Empirical | Examined the impact of the citizens’ assembly model of public engagement on health system planning and management |
| Green & Soyoola (2008) | 2008 | Nigeria | Africa | LMIC | Conceptual | Provided a review of cases that demonstrate voice and accountability initiative in the health sector in Nigeria, stems from the Partnerships for Transforming Health Systems Programme (PATHS). |
| Green & Kreuter (2002) | 2002 | N/A | N/A | N/A | Conceptual | Documented community coalitions against substance abuse process and their use of best practices |
| Groenewegen, Kerssens, Sixma, van der Eijk, & Boerma (2005) | 2005 | Multi-Country | Europe | HIC | Empirical | Description and explanation of international differences in health care users’ perspectives on the perceived importance of different aspects of health care (including provider communication, medication supply) |
| Gromulska, Supranowicz, & Wysocki (2014) | 2014 | Poland | Europe | HIC | Empirical | Measured responsiveness to hospital patients, among former patients, in Poland |
| Gulland (2006) | 2006 | England | Europe | HIC | Conceptual | Analysis of recent policy changes and proposals to suggest second-tier reviews of complaints |
| Gullo, Galavotti, & Altman (2016) | 2016 | Malawi | Africa | LMIC | Empirical | Review of evidence on the implementation of the Cooperative for Assistance and Relief Everywhere (CARE) Community Score Card |
| Gullo et al. (2017) | 2017 | Malawi | Africa | LMIC | Empirical | Evaluated the effects of a social accountability approach, Cooperative for Assistance and Relief Everywhere (CARE) Community Score Card, on reproductive health-related outcomes in Malawi |
| Gurung, Derrett, Gauld, & Hill (2017) | 2017 | Nepal | Asia | LMIC | Empirical | Analysis of complaint systems in primary health care (PHC) settings in Nepal. |
| Gurung, Derrett, Hill, & Gauld (2016) | 2016 | Nepal | Asia | LMIC | Conceptual | Described the governance challenges in the Nepalese primary health care system, arguing for expanded community engagement and a power shift towards local communities to overcome these challenges. |
| Gurung, Derrett, Hill, & Gauld (2018) | 2018 | Nepal | Asia | LMIC | Empirical | Described community representation in Nepal’s Health Facility Operation and Management Committees (HFOMCs) and the degree of influence of community representatives in the HFMC decision-making processes |
| Gurung, Gauld, Hill, & Derrett (2018) | 2018 | Nepal | Asia | LMIC | Empirical | Citizen’s Charter in a primary health- care setting of Nepal: An accountability tool or a “mere wall poster” |
| Gurung & Tuladhar (2013) | 2013 | Nepal | Asia | LMIC | Empirical | A case on good governance at (peripheral public health facilities) fostered through active engagement and capacity building of Health Facility Operation and Management Committees (HFOMCs), in Nepal |
| Ha, Mirzoev, & Morgan (2015) | 2015 | Vietnam | Asia | LMIC | Empirical | Complaint handling processes and the main influences on implementation in public hospitals in Vietnam. |
| Habibullah (2013) | 2013 | Pakistan | Asia | LMIC | Empirical | Measured the responsiveness of the federal health system to the needs of adults with physical disabilities in Islamabad, Pakistan |
| {Hadizadeh tala saz, 2019 #23155} | 2019 | Iran | Asia | LMIC | Empirical | Measure health system responsiveness (WHO) in obstetrics and gynecology departments of teaching hospitals in Mashhad, Iran |
| Hagg, Dahinten, & Currie (2018) | 2018 | N/A | N/A | LMIC | Empirical & conceptual | A scoping review on the use of social media for health-related purposes in low and middle-income countries |
| Hamal, de Cock Buning, et al. (2018) | 2018 | India | Asia | LMIC | Empirical | Analysed how accountability problems in public health system lead to maternal deaths and inequities in India |
| Hamal, Dieleman, De Brouwere, & de Cock Buning (2018) | 2018 | India | Asia | LMIC | Empirical & conceptual | Explored how social accountability mechanisms improve to maternal health, and how the results of these mechanisms are perceived in Gujarat, India |
| Hamal et al. (2019) | 2019 | Nepal | Asia | LMIC | Empirical | Documented existing social accountability in maternal health services in the far-western development region in Nepal |
| Hamid & Begum (2019) | 2019 | Bangladesh | Asia | LMIC | Empirical | Compared the responsiveness of outpatient care of urban primary health care providers in Bangladesh |
| Han, Hudson Scholle, Morton, Bechtel, & Kessler (2013) | 2013 |  | America | HIC | Empirical | Examined the level to which provider engage patients in quality improvement, an online survey in America |
| Haq, Sood, Yansen, Javeed, & Ali (2010) | 2010 | Pakistan | Asia | LMIC | Empirical | A case study using media (TV talk show) as tool to influence district level health policy in Pakistan |
| Harter et al. (2011) | 2011 | Germany | Europe | HIC | Conceptual | Patient participation and shared medical decision making in Germany with a specific focus on systemic influences on patient participation |
| Harvey & Powell (2019) | 2019 | England | Europe | HIC | Empirical | A participatory evaluation of the use of iPad information system for community-based care |
| Hawkins, DeLaO, & Hung (2016) | 2016 | Amsterdam | Europe | HIC | Conceptual | Described the role of social media in improving the patient experienced |
| Hefner et al. (2016) | 2016 | USA | America | HIC | Empirical | Explored how private sector Accountable Care Organizations (ACOs) defined their patient populations, and how they described ACO public engagement activities as part of the Population Health Management approach. |
| {Hernández, 2020 #23148} | 2020 | Guatemala | Asia | LMIC | Empirical | Examined initiatives for health accountability using a Network Analysis |
| Hernández et al. (2019) | 2019 | Guatemala | Asia | LMIC | Empirical | Examined how citizen-led initiatives’ actions strengthen grassroots networks, highlighting accountability achievements for indigenous populations in rural Guatemala |
| Ho, Labrecque, Batonon, Salsi, & Ratnayake (2015) | 2015 | Congo | Africa | FRAGILE/HUMANITARIAN | Empirical | Described the implementation of community scorecards within a community-driven reconstruction project (i.e. Tuungane “Let’s Unite” in Kiswahili) in two provinces of eastern Democratic Republic of Congo. |
| Hogg & Williamson (2001) | 2001 | Britain | Europe | HIC | Conceptual | Argues that the different roles that lay people play need to be explicitly defined for their contributions to be recognized and realized |
| Holeman, Cookson, & Pagliari (2016) | 2016 | N/A | N/A | LMIC | Empirical & conceptual | Mapped and described the existing digital good–governance interventions for strengthening health systems in LMICs |
| Househ, Borycki, & Kushniruk (2014) | 2014 | Multi-Country | Multiple Regions | BOTH | Empirical & conceptual | A review on patient empowerment through social media, focusing on the benefits and challenges |
| Hovey et al. (2010) | 2010 | Amsterdam | Europe | HIC | Empirical | Demonstrated ways in which patient involvement and feedback (through partnership) turn knowledge into action and help inform patients about safety practices and procedures. |
| Howard‐Grabman (2000) | 2000 | Peru | Asia | LMIC | Empirical | Described the experiences of two pilot projects (USAID-funded) that aimed to increase accountability through community mobilisation approaches to improve health in rural areas of Latin America. |
| Hsu, Chen, Hu, Yip, & Shu (2006) | 2006 | Taiwan | Asia | HIC | Empirical | Assessed the applicability of the responsiveness domains (dignity, autonomy, confidentiality, prompt attention, social support, basic amenities, and choices of providers) for the Taiwanese health system. |
| Human Rights Watch (2011) | 2011 | South Africa | Africa | LMIC | Empirical | Examined accountability frameworks and oversight mechanisms for maternal health care in facilities in the Eastern Cape, South Africa |
| Hussein & Okonofua (2012) | 2012 | N/A | N/A | N/A | Conceptual | Discusses confidential enquiries into maternal deaths in Nigeria to improve accountability and quality of care |
| Hussin, Ali, Reka, & Gjebrea (2015) | 2015 | Qatar | Asia | LMIC | Empirical | Examined patient and provider characteristics associated with health system performance measures (i.e. Access, utilization and health system responsiveness), compared these measures between Qataris and non-Qataris, and whether these measures changed over time |
| Ibe et al. (2017) | 2017 | Nigeria | Africa | LMIC | Empirical | Explored how purchasers in two financing mechanisms: The Formal Sector Social Health Insurance Programme (FSSHIP) operating under the Nigerian National Health Insurance Scheme (NHIS), and the tax-funded health system perform their roles in light of their responsibilities to the populations |
| Jackman (2010) | 2010 | Canada | Oceana | LMIC | Conceptual | Examined charter review (legal cases relating to access to health) as a health care accountability mechanism in Canada, |
| James, Villacis Calderon, & Cook (2017) | 2017 | USA | America | HIC | Empirical | An analysis of unstructured patient feedback (i.e. text comments from patients) on quality of healthcare service employing a text mining methodology |
| Janse van Rensburg et al. (2018) | 2018 | South Africa | Africa | LMIC | Empirical | Explored the extent and nature of state and non-state mental health service collaboration in the Mangaung Metropolitan District, Free State, South Africa |
| Jing et al. (2016) | 2016 | China | Asia | LMIC | Empirical | Compared levels of expectation towards the healthcare service (using domains of health system responsiveness) among HIV and non-HIV patients in China |
| Joarder (2015) | 2015 | Bangladesh | Asia | LMIC | Empirical | Examined Human Resources for Health responsiveness in rural Bangladesh, develop a scale to measure HRH responsiveness, and finally to demonstrate the application of the instrument |
| Joarder, George, Ahmed, Rashid, & Sarker (2017) | 2017 | Bangladesh | Asia | LMIC | Empirical | Explored the perceptions of system actors (users and providers) on what constitutes physician responsiveness in rural Bangladesh |
| Joarder, George, Sarker, Ahmed, & Peters (2017) | 2017 | Bangladesh | Asia | LMIC | Empirical | Comparison of responsiveness between public and private sector physicians in rural Bangladesh |
| Joarder, Mahmud, Sarker, George, & Rao (2017) | 2017 | Bangladesh | Asia | LMIC | Empirical | Developed a tool to measure physician responsiveness in rural Bangladesh |
| Johnston et al. (2019) | 2019 | Canada | America | HIC | Empirical | Explored citizens' perspectives on the use of publicly reported primary care performance information, results drawn from citizen‐patient deliberative dialogues held in three Canadian provinces. |
| Jolles & Wells (2017) | 2017 | USA | America | HIC | Empirical | Comparison of health service use between child Participatory Decision Making (PDM) service practices and regular services. The data was drawn from National Survey of Child Adolescent Well-being (NSCAW), United States, America |
| Jones, Rice, Robone, & Dias (2011) | 2011 | Multi-Country | Europe | HIC | Empirical | Examined inequality and polarisation in responsiveness across 25 European countries (using World Health Survey data) |
| (Kalter et al. (2011) | 2011 | India | Asia | LMIC | Empirical | Explored the impact and implications of the contextual factors on maternal death inquiry and response processes in India; they propose a model for introducing and scaling up a community based maternal death inquiry and response process |
| Kamal et al. (2018) | 2018 | N/A | N/A | N/A | Conceptual | Described a learning health system approach to codesigning and implementing a palliative care registry (a combination of patient and family inputs and clinical data to support person-centred care, quality improvement, accountability, transparency, and scientific research), United States. |
| Kanthor, Seligman, Dereje, & Tarantino (2014) | 2014 | N/A | N/A | N/A | Conceptual | A practical guide for governments/ministries of health and donors for with engaging civil society in health finance and governance, with a specific focus on social accountability tools. |
| Kapiriri, Norheim, & Heggenhougen (2003) | 2003 | Uganda | Africa | LMIC | Empirical | Explored system actors (i.e. Public and leaders e.g. Health planners at the national, district and community levels) experiences of participatory planning and priority setting processes in health, in a decentralized district in Uganda. |
| {Kapologwe, 2020 #23153} | 2020 | Tanzania | Africa | LMIC | Empirical | Assessed the health system responsiveness of primary health facilities in Tanzania prior to introduction of the Direct Health Facility Financing (DHFF) program |
| Karami-Tanha, Moradi-Lakeh, Fallah-Abadi, & Nojomi (2014) | 2014 | Iran | Asia | LMIC | Empirical | Measured the health system responsiveness for chronic heart failure patients in one of the main heart centres in Tehran (Iran) during 2012 – 2013. |
| Karuga et al. (2019) | 2019 | Kenya | Africa | LMIC | Empirical | A case study that explored the structure of two Community Health Committees networks (one rural and one urban), and an analysis of how health-related information flowed within these networks in Kenya. |
| Kashkoli, Zarei, Daneshkohan, & Khodakarim (2017) | 2017 | Iran | Asia | LMIC | Empirical | Investigated the relationship between hospital responsiveness (eight dimensions) and overall patient satisfaction in public and private hospitals in Tehran, Iran. |
| Katahoire et al. (2015) | 2015 | Uganda | Africa | LMIC | Empirical | Described early implementation experiences (first 2 years) of the Community and District Empowerment for Scale-up (CODES) project which used Citizen Report Cards and U-Reports for community monitoring in five pilot districts in Uganda |
| Kaufman, Liu, & Fang (2012) | 2012 | China | Asia | LMIC | Empirical | Present findings of a research on gender and health equity in rural China from 2002 to 2008 and offer recommendations for improving reproductive health through participatory planning |
| Kennedy, Green, & Payne-James (2017) | 2017 | United Kingdom | Europe | HIC | Empirical | Explored the frequency and nature of complaints made against health-care professionals (hcps) working in England, Wales and Northern Ireland. As well as an exploration into how these complaints are investigated and processed. |
| Kerrison & Pollock (2001) | 2001 | United Kingdom | Europe | HIC | Conceptual | A discussion on complaints management procedures as a mechanism for enhancing accountability to service users, using the NHS and Health Care in the United Kingdom as an example |
| Kerssens, Groenewegen, Sixma, Boerma, & van der Eijk (2004) | 2004 | Multi-Country | Europe | HIC | Empirical | Comparison of patient evaluations of health care quality in relation to WHO measures of achievement (including overall responsiveness) in 12 European countries |
| Khanna (2013) | 2013 | India | Asia | LMIC | Conceptual | Discussed the ethical issues in community-based monitoring and planning of health programmes within the National Rural Health Mission in India |
| Khunte & Walimbe (2011) | 2011 | India | Asia | LMIC | Conceptual | Documented the experiences and ideas of stakeholders involved in the implementation of Community Based Monitoring and Planning of Health Services in Maharashtra India |
| Kilewo & Frumence (2015) | 2015 | Tanzania | Africa | LMIC | Empirical | Explored factors that hinder community participation in developing and implementing Comprehensive Council Health Plan (CCHP) in Manyoni District, Tanzania |
| King et al. (2011) | 2011 | Glasgow | Europe | HIC | Empirical | Explored public perspectives (service users and lay representatives) on barriers and facilitators to e-health implementation and the priorities for future using citizen juries |
| Kite, Foley, Grunseit, & Freeman (2016) | 2016 | N/A | N/A | N/A | Empirical & conceptual | Identified specific features of Facebook posts that were associated with higher user engagement on Australian public health organisations’ Facebook pages |
| Kolasa, Dohnalik, Borek, Siemiątkowski, & Ścibiorski (2014) | 2014 | Poland | Europe | HIC | Empirical & conceptual | Explored Polish citizens perceptions regarding their participation in decision making processes in the healthcare sector |
| Kowal, Naidoo, Williams, & Chatterji (2011) | 2011 | Multi-Country | Asia | LMIC | Empirical | Comparison of health system responsiveness (for inpatient and outpatient) between China and other Asian countries, using World Health Survey data. |
| Kramer, Solomon, & Dingman (2009) | 2009 | Canada | Oceana | HIC | Conceptual | Described the approach employed by Cancer Care Ontario (CCO) to achieve accountability for the Wait Time Information System (WTIS) project, Canada |
| Kroneman, van Erp, & Groenewegen (2019) | 2019 | Netherlands | Europe | HIC | Empirical | Explored community members' willingness to participate in the organization, governance and policy making of primary care facilities in the Netherlands |
| Kruk, 2010) {Kruk, 2010 #23145} | 2010 | Multi-Country | Multiple Regions | LMIC | Empirical | Described and assessed the impact of primary care initiatives on a range of health system goals (improved health outcomes, responsiveness to patients and communities, equity and efficiency) in low- and middle-income countries. |
| Kypri & Maclennan (2014) | 2014 | New Zealand | Oceana | HIC | Empirical | Assessed public participation in local liquor licensing and related regulation in New Zealand, study was conducted in a period predating the new laws to examine change in participation patterns in the future. |
| {Lahariya, 2020 #23154} | 2020 | India | Asia | LMIC | Empirical & conceptual | The author states the case for improving health system responsiveness to advance universal health coverage |
| Lambert et al. (2016) | 2016 | USA | America | LMIC | Empirical | Examined whether there was an association between the use of a communication and resolution approach to patient harm and changes in medical liability processes and outcomes, in the United States. |
| Lapsley (2004) | 2004 | N/A | N/A | N/A | Conceptual | Argues for public involvement in health care which moves beyond the patient voice and is part of the health policy development |
| Lawton et al. (2017) | 2017 | United Kingdom | Europe | HIC | Empirical | Evaluated the efficacy of the Patient Reporting and Action for a Safe Environment (PRASE) intervention (which included a patient incident reporting tool) to improve the ward-level harm-free care (HFC) scores, in five hospitals in the UK. |
| Lechat et al. (2019) | 2019 | Burkina Faso | Africa | LMIC | Empirical | Presented the results of an evaluation of the Toll-Free Call Service Using an Interactive Voice Server (TF-IVS intervention) to strengthen health system governance and responsiveness, in Burkina Faso. |
| Lecoanet et al. (2014) | 2014 | France | Europe | HIC | Empirical | Analysed the functioning of an Experience Feedback Committee (EFC) and a Security Management Tool for the analysis of incident reporting in a medical department. |
| Lee (2012) | 2012 | USA | America | HIC | Empirical | Described the history, purpose, challenges, strategies, and progress of California’s Mental Health Oversight and Accountability Commission (MHSOAC) |
| Legare et al. (2011) | 2011 | Multi-Country | Multiple Regions | BOTH | Empirical & conceptual | Identified key components of patient and public involvement programs (PPIPs) used to develop and implement clinical practice guidelines (CPGs), a synthesis of existing programs |
| Leibert (2010) | 2010 | USA | America | HIC | Empirical | Examined the performance of state medical boards and identified factors impacting the performance in the United States. |
| Leskela, Viitanen, & Piirainen (2005) | 2005 | Multi-Country | Europe | HIC | Empirical | A comparison of patients’ perceptions and satisfaction of the physiotherapy instruction in a private outpatient facility and in a public physiotherapy facility. |
| Li, Chongsuvivatwong, Assanangkornchai, McNeil, & Cai (2018) | 2018 | China | Asia | LMIC | Empirical | A comparison of health system responsiveness (WHO) between HIV and non-HIV patients at infectious disease clinics in Yunnan, China |
| Liabsuetrakul, Petmanee, Sanguanchua, & Oumudee (2012) | 2012 | Thailand | Asia | LMIC | Empirical | Assessed the perceived responsiveness (WHO, 8 domains) and satisfaction amongst woman who gave birth at public facilities in Songkhla province Thailand |
| Lin, Yang, Chiang, & Lee (2019) | 2019 | Taiwan | Asia | LMIC | Empirical | Tested the psychometric properties (construct and concurrent validity and reliability) of the Incident‐Reporting Attitude Scale Used for Staff in Long‐Term Care Facilities (IRALTC) in Taiwan. |
| Lindsay, Sandall, & Humphrey (2012) | 2012 | London | Europe | HIC | Empirical | An ethnographic study to highlight the social nature of, and social processes (e.g. Group deliberation) around safety incident reporting in maternity services of a large inner-city National Health Service (NHS) hospital Trust, UK. |
| Listening Project (2011) | 2011 | N/A | N/A | N/A | Conceptual | A synthesis of literature and empirical data (interviews with staff of international assistance agencies and researchers) on the use of recipient/primary stakeholder feedback mechanisms in international aid efforts. |
| Litva et al. (2002) | 2002 | United Kingdom | Europe | HIC | Empirical | Explored members of the public perception regarding public involvement within the health care decision-making process at different levels of health care |
| Lodenstein, Dieleman, Gerretsen, & Broerse (2016) | 2016 | Multi-Country | Multiple Regions | LMIC | Empirical & conceptual | A realist review of cases of collective citizen action and advocacy, to identify key mechanisms of provider responsiveness. |
| Lodenstein, Ingemann, Molenaar, Dieleman, & Broerse (2018) | 2018 | Malawi | Africa | LMIC | Empirical | A narrative inquiry on the functioning of daily social accountability relations among maternal health care workers in rural Malawi. |
| Lodenstein et al. (2017) | 2017 | Multi-Country | Africa | LMIC | Empirical | Explored the social accountability practices that are facilitated by health facility committees in Benin, Guinea and the Democratic Republic of Congo. |
| Lodenstein et al. (2019) | 2019 | Malawi | Africa | LMIC | Empirical | Explored how health facility committees (HFCs) monitor the quality of health services and how they hold health workers accountable for their performance in Northern Malawi. |
| Lucas et al. (2019) | 2019 | United Kingdom | Europe | HIC | Empirical | A qualitative exploration of the disproportionate number of complaints against paramedics in the UK. |
| Lucock et al. (2015) | 2015 | United Kingdom | Europe | HIC | Empirical | Investigated the barriers and facilitators of effective implementation of a patient monitoring and enhanced feedback system in a UK National Health Service (NHS) psychological therapy service. |
| Lunevicius & Rahman (2012) | 2012 | Lithuania | Europe | HIC | Empirical | Described and assessed the performance (including financing, human resources and responsiveness) of Lithuanian trauma sector. |
| Luo, Wang, Lu, & Liu (2013) | 2013 | China | Asia | LMIC | Empirical | Evaluated the overall levels and distributions of community health services (CHS) responsiveness in urban China. |
| MacDonald et al. (2015) | 2015 | United Kingdom | Europe | HIC | Empirical | Explored the experience of service user governors (in mental health Foundation Trusts) and their capacity to hold boards accountable in the UK. |
| Maddalena (2006) | 2006 | Canada | America | HIC | Conceptual | An argument that the key to citizen participation in decision-making rests in the development of a clearly articulated accountability framework that delineates roles and responsibilities, within the Canadian context. |
| Madon & Krishna (2017) | 2017 | India | Asia | LMIC | Empirical | Described the challenges of accountability in invited spaces, the case of Village Health Sanitation and Nutrition Committees in Karnataka (India) |
| Mafuta et al. (2017) | 2017 | Congo | Africa | LMIC | Empirical | Described the development of a social accountability intervention that aimed to improve health services responsiveness in two health zones in the Democratic Republic of the Congo. |
| Mafuta et al. (2015) | 2015 | Congo | Africa | FRAGILE/HUMANITARIAN | Empirical | A situation analysis of existing social accountability mechanisms for maternal health services in two health zones in the Democratic Republic of Congo (Muanda and Bolenge) |
| Mafuta et al. (2016) | 2016 | Congo | Africa | FRAGILE/HUMANITARIAN | Empirical | Identified contextual factors influencing the implementation of social accountability initiatives for maternal health services in two health zones in the Democratic Republic of the Congo (Muanda and Bolenge). |
| Magruder, Fields, & Xu (2019) | 2019 | USA | America | HIC | Empirical | Examined of long-term care ombudsman complaint data with a focus on complaints of elder abuse, neglect and exploitation (ANE) and the nature of these complaints |
| Malfait, Van Hecke, De Bodt, Palsterman, & Eeckloo (2018) | 2018 | Belgium | Europe | HIC | Empirical | Examined patient and public involvement (PPI) in decision-making for the development of policy in six Flemish hospitals |
| Malhotra & Do (2013) | 2013 | India | Asia | LMIC | Empirical | Assessed the magnitude of socio-economic disparities in health system responsiveness in India, data drawn from the Study on Global Ageing and Adult Health (SAGE 2007–2008) |
| Malhotra & Do (2017) | 2017 | Multi-Country | Asia | LMIC | Empirical | Examined the association between the proportion of public health expenditure over total health expenditure (PPHE) with responsiveness for high- and low-income individuals, using World Health Survey data from 63 countries. |
| {Marathe, 2020 #23160} | 2020 | India | Asia | LMIC | Empirical & conceptual | Presented the discussions of a workshop aimed at ensuring accountability and responsiveness of the private health sector in India |
| Marston et al. (2016) | 2016 | N/A | N/A | N/A | Conceptual | Conceptual discussion on concepts of community participation and co-production (health services and communities working together) to achieve health goals. |
| Martinez & Kohler (2016) | 2016 | Brazil | America | LMIC | Empirical | A descriptive account of the challenges faced by civil society within Brazil’s Health Councils |
| Mathews, Ryan, & Bulman (2015) | 2015 | Canada | America | HIC | Empirical | Explored factors that shape patients’ satisfaction with overall wait times (i.e. from symptom to treatment). |
| McCoy, Hall, & Ridge (2011) | 2011 | Multi-Country | Multiple Regions | LMIC | Empirical & conceptual | A review of literature on the effectiveness of health facility committees (HFCs) and factors that influence the performance and effectiveness of HFCs |
| McCreaddie, Benwell, & Gritti (2018) | 2018 | United Kingdom | Europe | HIC | Empirical | A discourse analysis of patient complaint journey i.e. process and rhetoric of how patients formulate written complaints in UK |
| McEvoy & MacFarlane (2013) | 2013 | Ireland | Europe | HIC | Conceptual | Described community participation in primary care and the development of a ‘Joint Initiative on Community Participation in Primary Health Care in Ireland |
| McNamara (2006) | 2006 | Multi-Country | Multiple Regions | BOTH | Empirical & conceptual | Discussed aspects of the design and implementation of report cards including challenges of designing report cards and implementation decisions points. |
| McNatt et al. (2014) | 2014 | Ethiopia | Africa | LMIC | Empirical | Described the functioning of governing boards, explored the association between governing board functioning and hospital performance in Ethiopia. |
| McNeil & Mumvuma (2006) | 2006 | Multi-Country | Africa | LMIC | Conceptual | A synthesis of civil society-initiated social accountability practices in the public budgetary process in 10 Anglophone African countries—Ethiopia, Ghana, Kenya, Malawi, Namibia, South Africa, Tanzania, Uganda, Zambia and Zimbabwe. |
| Megbelayin, Babalola, Kurawa, Opubiri, & Okonkwo (2014) | 2014 | Nigeria | Africa | LMIC | Empirical | Determined satisfaction of ophthalmic patients with services at the outpatient eye clinic in University of Calabar Teaching Hospital, Nigeria  clinic |
| Mehata et al. (2017) | 2017 | Nepal | Asia | LMIC | Empirical | Identified the determinants of client satisfaction with maternity care in Nepal, using data from a nationally representative health facility survey |
| Mello, Studdert, Kachalia, & Brennan (2006) | 2006 | Multi-Country | Multiple Regions | BOTH | Conceptual | Described the proposals for the design of a health court system and highlighting the system’s advantages for improving patient safety and cultivation of a transparency regarding medical errors and injuries. |
| Melo, Martins, Jesus, Samico, & Santo (2017) | 2017 | Brazil | America | LMIC | Empirical | Investigated the responsiveness of services towards older adults at a reference unit of the Brazilian Unified Health System Brazilian Unified Health System |
| Miatello et al. (2018) | 2018 | Canada | America | HIC | Empirical | Examined the effectiveness of my experience MYEXP (suite of smartphone and web apps to gather care experiences of youth, family members, and service providers) as part of an experience-based codesign (EBCD) study in Ontario involving youth with mental disorders |
| Miller, Patton, Dobrow, Marshall, & Berta (2018) | 2018 | Multi-Country | Multiple Regions | HIC | Empirical | Explored organisational actors and stewardship functions that enable, support and delimit public involvement activity in two jurisdictions (England, United Kingdom, and Alberta, Canada) |
| Miller et al. (2014) | 2014 | Tanzania | Africa | LMIC | Empirical | Examined health system responsiveness of private clinics serving HIV patients in Dar es Salaam, Tanzania |
| Mills, 2017) | 2017 | N/A | N/A | N/A | Conceptual | Resilient and responsive health systems in a changing world |
| Mirzoev et al. (2017) | 2017 | N/A | N/A | N/A | Empirical & conceptual | A review of available literature on health systems responsiveness, summarised existing frameworks for and proposed an alternative framework to understand health systems responsiveness |
| Mirzoev & Kane (2018) | 2018 | Multi-Country | Multiple Regions | BOTH | Conceptual | A synthesis of literature on key strategies to improve patient complaint management systems/ procedures within health facilities |
| Mishima, Campos, Matumoto, & Fortuna (2016) | 2016 | Brazil | America | LMIC | Empirical | A qualitative exploration of the responsiveness (WHO, 8 domains) of a Family Health Unit (FHU) of a municipality in the interior of São Paulo, Brazil. |
| Mitton, Smith, Peacock, Evoy, & Abelson (2009) | 2009 | N/A | N/A | N/A | Empirical & conceptual | A scoping review on public engagement in priority setting and resource allocation (included health and non-health literature) |
| Mockford, Staniszewska, Griffiths, & Herron-Marx (2012) | 2012 | N/A | N/A | N/A | Empirical & conceptual | Examined how patient and public involvement (PPI) is being defined, theorized and conceptualized, and how the impact of PPI is captured or measured. Also, determined the impact of PPI on UK National Health Service (NHS) healthcare services and to identify the economic cost. |
| (Moeller, Rasmussen, & Nielsen (2016) | 2016 | Denmark | Europe | HIC | Empirical | Investigated how feedback mechanisms and learning processes (i.e. Patient safety incident reporting systems) were implemented at four Danish hospital units all located in one Danish region. |
| Mohammed, Bermejo, Souares, Sauerborn, & Dong (2013) | 2013 | Nigeria | Africa | LMIC | Empirical | Assessed the responsiveness (WHO) of health care services within a health insurance scheme in Nigeria |
| Molyneux, Atela, Angwenyi, & Goodman (2012) | 2012 | Multi-Country | Multiple Regions | LMIC | Empirical & conceptual | A review of literature on community accountability mechanisms in low or middle-income countries and proposed a conceptual framework. |
| Molyneux et al. (2016) | 2016 | Multi-Country | Africa | LMIC | Conceptual | Described experiences with community engagement research involving men who have sex with men, the paper presents discussions and outcomes of international meeting held at the Kenya Medical Research Institute-Wellcome Trust in Kilifi, Kenya. |
| Morone & Kilbreth (2003) | 2003 | USA | America | HIC | Conceptual | Discusses citizen participation in health policy, firstly by mapping the different types of participation and then contrasts the health sector (i.e. Relatively more top-down approaches) with education and crime policies (that more employ bottom-up approaches) |
| Morrison & Dearden (2013) | 2013 | United Kingdom | Europe | HIC | Empirical | A case study that demonstrated the use of representational artefacts (emotion maps, stories, and tracing paper) to enable meaningful public participation for service improvement for outpatient services for older people (which is known as Better Outpatient Services for Older People, BOSOP) in the UK. |
| Mossalam et al. (2013) | 2013 | Egypt | Africa | LMIC | Empirical | Compared the level of health system responsiveness (WHO) of the Health Insurance Organization (HIO) and private healthcare systems in Alexandria |
| Mosconi, Colombo, Satolli, & Liberati (2007) | 2007 | Italy | Europe | HIC | Empirical | Described the development and processes of partecipasalute i.e. An Italian partnership among lay people, patients' associations and the scientific/medical community |
| Mubyazi, Mushi, Kamugisha, et al. (2007) | 2007 | Tanzania | Africa | LMIC | Empirical | Described community perceptions on Health Sector Reform (HSR) and their participation in setting health priorities, in Lushoto and Muheza districts, Tanzania |
| Mubyazi, Mushi, Shayo, et al. (2007) | 2007 | Tanzania | Africa | LMIC | Empirical | Explored the views of villagers regarding the existence and functioning of local primary healthcare (PHC) committees and village health workers (VHWs) in Mkuranga District, Tanzania |
| Mukinda, Van Belle, George, & Schneider (2019) | 2019 | South Africa | Africa | LMIC | Empirical & conceptual | A case study of local accountability mechanisms for maternal, new-born and child health (MNCH) in South Africa (involving a review of national policies, programme reports, and other literature related to MNCH, and in-depth research in one district) |
| Mullen, Hughes, & Vincent-Jones (2011) | 2011 | England | Europe | HIC | Conceptual | A discussion on the democratic function of different public participation arrangements in healthcare governance in England |
| Mulumba, London, Nantaba, & Ngwena (2018) | 2018 | Multi-Country | Africa | LMIC | Empirical | Developed and tested models of good practice for health committees in South Africa and Uganda |
| Mulvale, Chodos, Bartram, MacKinnon, & Abud (2014) | 2014 | Canada | America | HIC | Empirical | Examined the role of civil society engagement in the development of the first Mental Health Strategy for Canada and developed a framework of specific goals to guide the development of the Strategy, and ultimately, the creation of the Strategy itself. |
| Munro & Duckett (2016) | 2016 | China | Asia | LMIC | Empirical | Identified factors associated with health-care system satisfaction in China |
| Murante, Seghieri, Vainieri, & Schafer (2017) | 2017 | Multi-Country | Europe | HIC | Empirical | Investigated the relationship between the responsiveness of primary care, domestic health expenditure and remuneration of primary care doctors across Europe. |
| Murante, Vainieri, Rojas, & Nuti (2014) | 2014 | Italy | Europe | HIC | Empirical | Examined the association between patient feedback (and health-professional awareness of patient survey) and the improvement in provider communication with patients in Italy |
| Murray & Frenk (2001) | 2001 | N/A | N/A | N/A | Conceptual | A discussion on the World Health Report 2000 and foregrounding evidence-based health policy |
| Murray & Evans (2003) | 2003 | N/A | N/A | N/A | Empirical & conceptual | The world health report on measuring health systems performance methods |
| Murray & Frenk, 1999) | 1999 | N/A | N/A | N/A | Conceptual | A WHO framework for health system performance assessment |
| Murray & Frenk (2000) | 2000 | N/A | N/A | N/A | Conceptual | Proposes the WHO framework to assess health system performance. |
| Murthy & Klugman (2004) | 2004 | Multi-Country | Asia | NOT CLEAR | Conceptual | Examined community participation and accountability in World Bank-supported health sector reforms in Asia and the implications for sexual and reproductive health services |
| Musgrove (2003) | 2003 | N/A | N/A | N/A | Conceptual | A critique of the methods of the World Health Report 2000 |
| Nâdâșan & Abrám (2016) | 2005 | Romania | Europe | LMIC | Empirical | Assessed the compliance of the Romanian health websites to the credibility criteria for health-related websites. |
| Nagy, Chiarella, Bennett, Walton, & Carney (2018) | 2018 | Australia | Oceana | HIC | Empirical | Described the adaptation of the “patient journey” technique for the comparison of two complaints management systems |
| Najafi, Karami-Matin, Rezaei, Rajabi-Gilan, & Soofi (2016) | 2016 | Iran | Asia | LMIC | Empirical | Examined health system responsiveness (WHO) after Health Sector Evolution Plan in Kermanshah, Western Iran. |
| Nakajima, Kurata, & Takeda (2005) | 2005 | Japan | Asia | HIC | Empirical | A web-based incident reporting system and multidisciplinary collaborative projects for patient safety in a Japanese hospital |
| Nambisan, Gustafson, Hawkins, & Pingree (2016) | 2016 | USA | America | HIC | Empirical | An evaluation of the impact of social support and responsiveness that patients experience in a health-care organizations online community on patients’ perceptions regarding the health-care organization service quality |
| Narayanan & Greco (2014) | 2014 | Australia | Oceana | HIC | Empirical | Described the development and use of a dental patient questionnaire (DPQ) to collect patient feedback data. |
| Nathan, Johnston, & Braithwaite (2011) | 2011 | Australia | Oceana | HIC | Empirical | Examined staff views about legitimacy of different roles for community representatives (on health service committees) as part of a formal Community Participation Program (CPP) in an Area Health Service (AHS) in Australia. |
| Navarro (2001) | 2001 | N/A | N/A | N/A | Conceptual | This article analysed the concepts and methods used in WHR 2000 (Health Systems: Improving Performance) |
| {Nepal, 2019 #23161} | 2019 | Nepal | Asia | LMIC | Empirical | An exploration of social accountability interventions for improved maternal health services in Nepal |
| Njeru, Blystad, Nyamongo, & Fylkesnes (2009) | 2009 | Kenya | Africa | LMIC | Empirical | Assessed the applicability of the responsiveness tool developed by WHO for voluntary HIV counselling and testing services |
| Nowotny, Loh, Lorenz, & Wallace (2019) | 2019 | Australia | Oceana | HIC | Conceptual | Summarized the patient complaint and medico-legal landscape in the public hospital system of Australia and specifically Victoria |
| Nswilla et al. (2019) | 2019 | Tanzania | Africa | LMIC | Empirical | Evaluated the effects of Direct Health Facility Financing on health system performance in Tanzania. |
| Nunes, Brandao, & Rego (2011) | 2011 | N/A | N/A | N/A | Conceptual | Determined the importance of the principle of public accountability in healthcare regulation, stressing the fact that Sunshine Regulation—as a direct and transparent control over health activities—is vital for an effective regulatory activity |
| Nunes, Rego, & Brandao (2009) | 2009 | N/A | N/A | N/A | Conceptual | Determine whether an independent healthcare regulation was an important tool for public accountability (and fair processes) for setting limits to healthcare. Regulation as a tool for public accountability |
| Nxumalo et al. (2018) | 2018 | Kenya & South Africa | Africa | LMIC | Empirical | Experiences of front-line managers at subnational level in Kenya and South Africa demonstrate the value of interpersonal or relational interactions to the appropriate functioning of many accountability mechanisms |
| Nxumalo (2013) | 2013 | South Africa | Africa | LMIC | Empirical | Examine the implementation of community health worker-provided services and a comparison of three case studies to identify enabling and constraining factors |
| O’Meara, Tsofa, Molyneux, Goodman, & McKenzie, 2011) | 2011 | Kenya | Africa | LMIC | Empirical | Described challenges in the implementation of national planning guidelines (Annual Operations Plan Four or AOP4 2008-2009), how these influenced final plans and budgets in Kilifi, Kenya |
| J. Ocloo & Matthews, 2016) | 2016 | N/A | N/A | N/A | Empirical & conceptual | A narrative review on patient and public involvement in healthcare improvement |
| Ocloo & Fulop, 2012) | 2012 | United Kingdom | Europe | HIC | Conceptual | Identified key underlying drivers for patient and public involvement (PPI) in health and social care; provide suggestions on developing the PPI agenda in patient safety. |
| O'Connell et al., 2018) | 2018 | Not Clear | Not Clear | NOT CLEAR | Empirical | Described and measure reported medical errors related to process variance in 17 emergency departments in the Paediatric Emergency Care Applied Research Network from 2007 to 2008. |
| Oguntunde et al., 2018) | 2018 | Nigeria | Africa | LMIC | Empirical | An evaluation of facility health committees and its association with community mobilization, utilization and quality of maternal and child health services in northern Nigeria |
| Ogwang, Najjemba, Tumwesigye, & Orach, 2012) | 2012 | Uganda | Africa | LMIC | Empirical | Identified types of community involvement and examined factors influencing the level of community involvement in the management of obstetric emergencies |
| O'Hara et al., 2017) | 2017 | England | Europe | HIC | Empirical | An exploratory pilot testing three mechanisms (face-to-face interviewing by researchers, a paper-based form and a ‘patient safety hotline’) for collecting data on safety concerns from patients during their hospital stay. |
| O'Hara et al., 2016) | 2016 | England | Europe | HIC | Empirical | Developed and tested an intervention (PRASE) for receiving and acting upon patient feedback about safety in an hospital in England |
| (Okafor et al., 2015) | 2015 | USA | America | HIC | Empirical | The design and implementation of a web-based emergency department -specific incident reporting system |
| Okeyo, Lehmann, & Schneider (2020) | 2020 | South Africa | Africa | LMIC | Empirical & conceptual | The impact of differing frames on early stages of intersectoral collaboration: the case of the First 1000 Days Initiative in the Western Cape Province |
| Olayo, Wafula, Aseyo, Loum, & Kaseje (2014) | 2014 | Kenya | Africa | LMIC | Empirical | A quasi-experimental assessment of the effectiveness of the Community Health Strategy on health outcomes in Kenya |
| Oliver, Armes, & Gyte (2009) | 2009 | United Kingdom | Europe | HIC | Empirical | Described the inputs and influences of public involvement in setting the agenda for the National Health Service Health Technology Assessment (HTA) program |
| Ortiz et al. (2003) | 2005 | Multi-Country | Multiple Regions | BOTH | Empirical | Presented the overall level of responsiveness and responsiveness inequality scores for 16 OECD countries |
| O'Shea, Boaz, & Chambers (2019) | 2019 | England | Europe | HIC | Empirical | A qualitative study of patient and public involvement in a clinical commissioning group (CCG) in the UK -focusing on activities that contained elements of PPI this involved researchers conducting observations of meetings, |
| Padarath & Friedman (2008) | 2008 | South Africa | Africa | LMIC | Empirical | Assessed the functioning and effectiveness of clinic committees in primary health settings in South Africa |
| Pagatpatan & Ward (2017) | 2017 | N/A | N/A | LMIC | Empirical & conceptual | Realist synthesis of enabling factors for public participation in health policy and planning |
| Paina, Saracino, Bishai, & Sarriot (2019) | 2019 | N/A | N/A | N/A | Empirical & conceptual | A review on the monitoring and evaluation social accountability efforts in health |
| Panda, Zodpey, & Thakur (2016) | 2016 | India | Asia | LMIC | Empirical | Aimed to assess the perception of Rogi Kalyan Samitis (RKS) members about their roles, involvement and practices with respect to local decision making and management of decision-making health units (DMHUs); |
| Park, Kim, You, Lee, & Park (2014) | 2014 | Korea | Asia | LMIC | Empirical | Identified the patterns of participation in both the public and private sectors using a policy-network analysis at the community level |
| Paschke, Dimancesco, Vian, Kohler, & Forte (2018) | 2018 | N/A | N/A | N/A | Conceptual | Provides a conceptual discussion of how transparency enhances accountability for better access to medication in national pharmaceutical system |
| Patterson & Pace (2016) | 2016 | USA | America | HIC | Empirical | Determine the extent to which organisational factors (punitive work climate, inadequate error feedback to staff, or insufficient preventative procedures) are associated with decreased frequency of near-miss error reporting among hospital pharmacists |
| Pearce (2016) | 2016 | Pacific Islands Countries and Territories (Picts) | Oceana | HIC | Empirical | Presented three studies to examine eye care in Pacific Islands Countries and Territories (PICTs) through a health systems-strengthening lens. The second study qualitatively explored communities' perceived responsiveness (WHO) |
| Peltzer (2009) | 2009 | South Africa | Africa | LMIC | Empirical | Evaluated the degree of perceived responsiveness with outpatient and inpatient healthcare in South Africa (data from the Global Study on Ageing and Adult Health (SAGE) survey) |
| Peltzer & Phaswana-Mafuya (2012) | 2012 | South Africa | Africa | LMIC | Empirical | Evaluated the perceived responsiveness with outpatient and inpatient healthcare in South Africa among older adults (data from the Global Study on Ageing and Adult Health (SAGE) survey) |
| Penney (2004) | 2004 | Canada | America | HIC | Empirical & conceptual | Conceptual clarification on the concept of accountability within the health field and examined the issues related to improving health system accountability in Canada |
| Penno & Gauld (2017) | 2017 | New Zealand | Oceana | HIC | Empirical | Examined 20 New Zealand’s District Health Boards (DHB) spending patterns on for external consultancies. |
| Perera, Seneviratne, & Fernando (2011) | 2011 | Sri Lanka | Asia | LMIC | Empirical | The development and validation of a Health System Responsiveness Assessment Questionnaire (HESRAQ) in relation to family planning (FP) services in Sri Lanka |
| Perera, Mwanri, de A Seneviratne, & Fernando (2012) | 2012 | Sri Lanka | Asia | LMIC | Empirical | Assessed health systems responsiveness and its correlates of family planning (FP) services in Colombo district, Sri Lanka. |
| Perrott (2013) | 2013 | Australia | Oceana | HIC | Empirical | A research-based case discussion on codesign in the context of health service delivery. |
| Peters et al. (2007) | 2007 | Afghanistan | Asia | FRAGILE/HUMANITARIAN | Empirical | The development and implementation of balanced scorecard (BSC) for to monitor the progress of basic health services in Afghanistan |
| Phaswana-Mafuya, Peltzer, Hoosain, & Maseko (2017) | 2017 | South Africa | Africa | LMIC | Empirical | Assessed the psychometric properties of a patient satisfaction survey tool developed by the Health Systems Trust (2004) for South Africa |
| Phiri, Fylkesnes, Ruano, & Moland (2014) | 2014 | Zambia | Africa | LMIC | Empirical | Explored how users and providers perception on the low utilization of health facilities during childbirth |
| Pichert et al. (2013) | 2013 | USA | America | HIC | Empirical | The development of a peer messenger process to address “high-risk” physicians identified through analysis of unsolicited patient complaints, a proxy for risk of lawsuits |
| Pieterse (2019) | 2019 | Sierra Leone | Africa | FRAGILE/HUMANITARIAN | Empirical | Explored the utility of social accountability interventions in the primary healthcare sector in a fragile state i.e. Sierra Leone |
| Polancich, Poe, Hackney, & Williamson (2017) | 2017 | Not Clear | Not Clear | NOT CLEAR | Empirical | A case study demonstrating the use of technology to develop a chief nursing officer accountability scorecard |
| Poles et al. (2014) | 2014 | Tanzania | Africa | LMIC | Empirical | Investigated the relationship between health system responsiveness, patient factors and visit non-adherence in 16 PEPFAR-supported HIV/ AIDS clinics in Dar es Salaam |
| Pongsupap & Van Lerberghe (2006a) | 2006 | Thailand | Asia | LMIC | Empirical | Compared the responsiveness, degree of patient-centeredness, adequacy of therapeutic decisions between 37 self-styled family practices and 37 conventional types of services (i.e. Public hospital outpatient departments (OPDs), private clinics and private hospital OPDs) in 16 provinces in Thailand |
| Pongsupap & Van Lerberghe (2006b) | 2006 | Thailand | Asia | LMIC | Empirical | To measure responsiveness (basic amenities, prompt attention, communication, dignity and trust) of 37 self-styled family practices and 37 conventional types of services (i.e. Public hospital outpatient departments (OPDs), private clinics and private hospital OPDs) in 16 provinces in Thailand |
| Post, Sanjay Agarwal, & Venugopa (2014) | 2014 | Multi-Country | Multiple Regions | BOTH | Conceptual | A guideline on the development and implementation of community scorecards in improving service delivery |
| Potts & Hunt (2008) | 2008 | N/A | N/A | N/A | Conceptual | A monograph on the role of accountability and the notion of right to health and highest standard of health |
| Prakash & Singh (2013) | 2013 | India | Asia | LMIC | Empirical | Investigate whether the New Public Management reforms have any consequences on the changing the relationship of the state with the citizen, particularly regarding responsiveness |
| Pratt & Hyder (2015) | 2015 | Multi-Country | Multiple Regions | LMIC | Conceptual | Proposes how the ethical concept of responsiveness might be understood and applied in the context of externally funded HSR in LMICs. |
| Quinn & Otteson (2019) | 2019 | use | America | HIC | Empirical | A participatory research approach that involved community members with mental health issues and their support systems in the development and adoption of a mental health identification card |
| Radford, Sheps, Pink, & Ricketts (2007) | 2007 | USA | America | HIC | Empirical | The design and implementation of a comparative performance scorecard for federally funded community health centres in North Carolina |
| Rafiei et al. (2016) | 2016 | Iran | Asia | LMIC | Empirical | Evaluate the responsiveness of a sample of hospitals in Yazd province (Central Iran); perspectives of hospital managers. |
| Rahman, Singh, & Madhavan (2019) | 2019 | Multi-Country | Multiple Regions | LMIC | Empirical | Examines the disability-based disparity in outpatient health system responsiveness among the older adults in five low to upper middle-income countries (data drawn from Study on Global Ageing and Adult Health) |
| Rajamani, Bieringer, Sowunmi, & Muscoplat (2017) | 2017 | USA | America | HIC | Empirical | Obtained stakeholder feedback on the access and utilization of clinical decision support for immunizations (CDSI) from the Minnesota Immunization Information Connection (MIIC |
| Ramiro et al. (2001) | 2001 | Philippines | Asia | LMIC | Empirical | Analysed the role of local health boards in enhancing community participation and empowerment under a decentralized system in the Philippines |
| Rashidian et al. (2011) | 2011 | Iran | Asia | LMIC | Empirical | Measured the health system responsiveness and the factors affecting responsiveness in Iran health system. |
| Rawson et al. (2018) | 2018 | United Kingdom | Europe | HIC | Empirical | The development and testing of a practical tool to capture citizens priorities for infection research. |
| Reeleder, Goel, Singer, & Martin (2006) | 2006 | Canada | America | HIC | Empirical | A case analysis of priority setting in a state health care context in Ontario, Canada. The analysis specifically focused on how the implementation of hospital accountability arrangement meets the conditions of accountability for reasonableness’’ (A4R) i.e. An international ethical framework for priority setting |
| Reeve et al. (2015) | 2015 | Australia | Oceana | HIC | Empirical | Described a community-initiated health service partnership between a community-controlled Aboriginal health organisation, a government hospital and a population health unit as an attempt to improve primary health-care service in a remote region of Australia |
| Reid et al. (2017) | 2017 | New Zealand | Oceana | HIC | Conceptual | This viewpoint describes an equity-based approach to responsiveness to Māori and highlight four key areas that require careful consideration, namely: (1) relevance to Māori; (2) Māori as participants; (3) promoting the Māori voice, and; (4) human tissue. |
| Reinders et al. (2011) | 2011 | N/A | N/A | N/A | Empirical & conceptual | A literature review on the effect of patient feedback interventions to improving physicians’ consultation skills including communication and interpersonal |
| Renedo & Marston (2015) | 2015 | United Kingdom | Europe | HIC | Empirical | Ethnographic study on how participatory spaces and citizenship were co-constituted in participatory healthcare improvement efforts in the UK (NHS) |
| Rezaei et al. (2016) | 2016 | Iran | Asia | LMIC | Empirical | Assessed the patients' perceived quality of the service of educational hospitals affiliated with Kermanshah University of Medical Sciences in 2015 |
| Rice, Robone, & Smith (2011) | 2011 | Multi-Country | Multiple Regions | BOTH | Methodological | Analysed of the validity of the vignette approach to correct for heterogeneity in reporting health system responsiveness |
| Rice, Robone, & Smith (2008) | 2008 | Multi-Country | Multiple Regions | LMIC | Empirical | The measurement and comparison of health system responsiveness using the method of anchoring vignettes to adjust survey reports of responsiveness for reporting heterogeneity (methodology for measuring responsiveness for international comparison) |
| Rice, Robone, & Smith (2010) | 2010 | Multi-Country | Europe | HIC | Empirical | Comparison of public sector performance of 17 European Union countries and corrected systematic reporting behaviour through the use of anchoring vignettes |
| Rice, Robone, & Smith (2010) | 2012 | Multi-Country | Multiple Regions | BOTH | Methodological | Explores the use of anchoring vignettes to adjust survey reports of health system performance for differential reporting behaviour by using data contained within the World Health Survey |
| Rifkin (2014) | 2014 | N/A | N/A | N/A | Empirical & conceptual | A review of literature on the associations between community participation and health outcomes |
| Ringold, Holla, Koziol, & Srinivasan (2012) | 2012 | Multi | Multiple Regions | BOTH | Empirical & conceptual | Book documents a diverse set of cases and initiatives of social accountability approaches in human development |
| Rise, Eriksen, Grimstad, & Steinsbekk (2012) | 2012 | Norway | Europe | HIC | Empirical | Investigated the effect of two brief feedback scales in mental health out-patient treatment on patient satisfaction six weeks after starting treatment |
| Rise, Eriksen, Grimstad, & Steinsbekk (2016) | 2016 | Norway | Europe | HIC | Empirical | Investigated the long-term effectiveness of using the Partners for Change Outcome Management System (PCOMS) feedback scales in out-patient mental health consultations on mental health symptoms and patient activation. |
| Rise et al. (2013) | 2013 | Norway | Europe | HIC | Empirical | Investigated and compared service users and providers definitions of patient and public involvement. |
| Ristea, Stegaroiu, Ioan-Franc, & Dinu (2009) | 2009 | Romania | Europe | HIC | Empirical | Presented ways of assessing the indicators that comprise health system responsiveness according to the WHO |
| Robinson et al. (2003) | 2003 | Canada | America | HIC | Empirical | Development of a balanced scorecard for public health and the use of modified nominal group technique to reach consensus on the components of the scorecard |
| Robone, Rice, & Smith (2010) | 2010 | Multi-Country | Multiple Regions | BOTH | Empirical | Simultaneously analysed plausible country-level characteristics as potential determinants of health system responsiveness |
| Robone, Rice, & Smith (2011) | 2011 | Multi-Country | Multiple Regions | BOTH | Empirical | Investigated the country-level drivers of health system responsiveness (data drawn from World Health Survey) |
| Rosenberg & Rosen (2012) | 2012 | Multi-Country | Oceana | HIC | Conceptual | A review of the evidence from both Australia and aboard whether mental health commissions can drive reform through better resourcing, services, accountability and stakeholder engagement |
| Rossouw & Smith (2017) | 2017 | South Africa | Africa | LMIC | Empirical | Explored the association between education levels and reporting behaviour in terms of health-system responsiveness South Africa (data drawn from WHO SAGE) |
| Rottger et al. (2015) | 2015 | Russia | Europe | LMIC | Empirical | Assess the level of perceived health system responsiveness to patients with chronic diseases in ambulatory care in Germany and to analyse the determinants of health system responsiveness and its distribution across different population groups. |
| Rottger, Blumel, Fuchs, & Busse (2014) | 2014 | Russia | Europe | LMIC | Empirical | Test the applicability of the World Health Organization’s (WHO’s) responsiveness concept for chronic disease care in Germany |
| Röttger, Blümel, Köppen, & Busse (2016) | 2016 | Russia | Europe | LMIC | Empirical | Assessed the level of forgone care among the chronically ill in Germany and subsequently analyse which factors are associated with forgone care. |
| Röttger, Blümel, Linder, & Busse (2017) | 2017 | Russia | Europe | LMIC | Empirical | Analysed the differences in the perceived health system responsiveness (WHO) between disease management programs participants and non-participants |
| Roussos & Fawcett (2000) | 2000 | N/A | N/A | N/A | Conceptual | Reviewed collaborative partnership as strategy improving community health |
| Rowe & Shepherd (2002) | 2002 | Britain | Europe | LMIC | Empirical | An analysis of a current Labour (New public management NPM) policy towards public participation and reports on the response of primary care groups (PCGs). |
| Roy (2008) | 2008 | India | Asia | LMIC | Empirical | A discussion on the good governance-civil society interface |
| Rozenblum, Greaves, & Bates (2017) | 2017 | No | N/A | N/A | Conceptual | A conceptual discussion on the role of social media around patient experience and engagement |
| Rubrichi, Battistotti, & Quaglini (2014) | 2014 | Italy | Europe | HIC | Empirical | Describe a system for the automatic evaluation of users’ perception of the quality of SMScup, a reminder system for outpatient visits based on short message service (SMS).The final purpose was the creation of a closed-loop control system for the outpatient service, where patients’ complaints and comments represent a feedback that can be used for a better implementation of the service itself |
| Saadat, Panah, Noroozi, & Alamdari (2017) | 2017 | Iran | Asia | LMIC | Empirical | Investigated the relationship between the Implementation of Healthcare Reform and the patient’s satisfaction at Yasuj hospitals |
| Sajjadi, Moradi-Lakeh, Nojomi, Baradaran, & Azizi (2015) | 2015 | Iran | Asia | LMIC | Empirical | Assessed the responsiveness (WHO) of the health system for patients with diabetes in Tehran, Iran. |
| Schaad, Bourquin, Panese, & Stiefel (2019) | 2019 | Switzerland | Europe | HIC | Empirical | Explored physicians' experience of hospital users’ complaints and the associated mediation process. |
| {Schaaf, 2020 #23147} | 2020 | N/A | N/A | N/A | Conceptual | Authors identified key cross-cutting considerations for fostering accountability for SRHR, including: macro-level politics and ruling ideologies, health system responsiveness, community voices, and the complexity of health systems. |
| Schaaf et al. (2018) | 2018 | Guatemala & India | Multiple Regions | LMIC | Empirical | A multiple case study on the implementation of ICT (Information and Communication Technology) and health accountability projects, described the operational value of ICT tools. |
| Schaaf & Dasgupta (2019) | 2019 | India | Asia | LMIC | Empirical | A descriptive case study of a social accountability project undertaken by a nongovernmental organization (SAHAYOG) in Uttar Pradesh, India. |
| Marta Schaaf et al. (2018) | 2018 | N/A | N/A | N/A | Conceptual | Offer several propositions for further conceptual development and research relating to the role of community health workers and accountability |
| Schaaf, Topp, & Ngulube (2017) | 2017 | Zambia | Africa | LMIC | Empirical | A theory-driven qualitative study of the context, mechanisms and outcomes of Citizen Voice and Action (CVA, social accountability program), implemented by World Vision (WV) in Zambia. |
| Schnitzer, Kuhlmey, Adolph, Holzhausen, & Schenk (2012) | 2012 | Germany | Europe | HIC | Empirical | An analysis of the topics of complaint raised most frequently and profiles of groups most affected between 2004 and 2007 in Germany. |
| Schwartz, Price, Deber, Manson, & Scott (2014) | 2014 | Canada | America | HIC | Empirical | We examined how well the challenges of holding local boards of health accountable were addressed in Ontario, Canada during the early stages of implementation of a new accountability policy |
| Sciamanna, Novak, Houston, Gramling, & Marcus (2004) | 2004 | Rhode Island | America | HIC | Empirical | Examined the potential effects of a stand-alone computer application had on primary care patients’ satisfaction with care |
| Scott & Grant (2018) | 2018 | N/A | N/A | N/A | Empirical & conceptual | Synthesized experiences of complaint procedures from patients and health professionals’ perspectives to identify enablers and challenges |
| Scott & Danel (2016) | 2016 | N/A | N/A | N/A | Conceptual | Described the progress made toward improving accountability for maternal and new-born health since 2011 (the United Nations Commission on Information and Accountability for Women's and Children's made specific recommendations) and identifies challenges. |
| Scott, Heavey, Waring, Jones, & Dawson (2016) | 2016 | Not Clear | Not Clear | NOT CLEAR | Empirical | Develop and validated a mechanism for patient feedback on safety experiences following a care transfer between organisations. |
| Scott, Jessani, Qiu, & Bennett (2018) | 2018 | N/A | N/A | N/A | Empirical & conceptual | Described a participatory multi-stage research priority setting exercise to identify priority research questions relevant to improving accountability within health systems |
| Scuffham, Whitty, Taylor, & Saxby (2010) | 2010 | Multi-Country | Multiple Regions | HIC | Empirical | A pilot study to test a discrete-choice instrument designed to elicit preference weights surrounding health system attributes including level of health, equity, responsiveness and healthcare financing |
| Serapioni & Duxbury (2014) | 2014 | Italy | Europe | HIC | Empirical | An evaluation of Mixed Advisory Committees in Italian health districts and hospitals |
| Serapioni & Matos (2014) | 2014 | Multi-Country | Europe | HIC | Conceptual | Describe experiences of the designed and implementation citizen participation initiatives in the three countries, including the key features, main critical issues and contradictions that emerged over time. |
| Serrate, Lausanne, Jean-Claude, Espinosa, & Gonzalez (2007) | 2007 | Cuba | America | LMIC | Conceptual | A case study of intersector practices in health in Cuba; arguing for a social construction lens for health |
| Shaikh, Miraldo, & Renner (2018) | 2018 | India | Asia | LMIC | Empirical | Examined the relationship between social class i.e. Caste of an individual and waiting time at health facilities—one dimension of the WHO responsiveness concept. |
| Sharma & Grumbach (2017) | 2017 | Multi-Country | Multiple Regions | HIC | Conceptual | Historical and policy context analysis for the emerging interest in patient engagement at the primary care practice level in the USA and UK- a review of reviews. |
| Shaw, Rohde, & Stice (2016) | 2016 | USA | America | HIC | Empirical | Examined the effect of qualitative participant feedback between three intervention modalities i.e. Clinician-delivered groups, peer-delivered groups, and internet based for eating disorder prevention program from a controlled trial. |
| Sheard et al. (2017) | 2017 | England | Europe | HIC | Empirical | Developed the Patient Feedback Response Framework as a way to understand why UK hospital staff find it difficult to make improvements based on patient feedback. |
| Shepherd, Sanders, Doyle, & Shaw (2015) | 2015 | Not Clear | Not Clear | NOT CLEAR | Empirical | A case study that assessed the role of Twitter as possible source of feedback to mental health service providers |
| Shiyanbola, Smith, Mansukhani, & Huang (2016) | 2016 | USA | America | HIC | Empirical | Explored patient feedback on five newly designed prescription warning labels (PWLs) |
| Shrivastava, Shrivastava, & Ramasamy (2013) | 2013 | N/A | N/A | N/A | Conceptual | Description of community monitoring approaches |
| Shukla & Sinha (2014) | 2014 | India | Asia | LMIC | Conceptual | Described the implementation, successes and challenges of community-based monitoring approach to planning health in Maharashtra, India |
| Simpson & House (2002) | 2002 | N/A | N/A | N/A | Empirical & conceptual | Reviewed the effects of user involvement in the delivery and evaluation of mental health services. |
| Siriwardena & Gillam (2014) | 2014 | N/A | Europe | HIC | Conceptual | Described approaches and techniques used to involve users and public in improving healthcare |
| Sirven, Santos-Eggimann, & Spagnoli (2008) | 2008 | Multi-Country | Europe | HIC | Empirical | Methodological approach to measure and corrects the incomparability of the SHARE survey on health care responsiveness (2006-07) in ten European countries. |
| Skeen et al. (2010) | 2010 | South Africa | Africa | LMIC | Empirical | Assessed South Africa’s progress in intersectoral collaboration for mental health, and provide recommendations for intersectoral collaboration, to generate lessons for other low- and middle-income countries |
| Slowiak & Huitema (2015) | 2015 | USA | America | HIC | Empirical | Evaluate the effects of 2 different interventions (i.e. Feedback regarding customer satisfaction with wait time and combined feedback and goal setting) on wait time in a hospital outpatient pharmacy |
| Slutsky et al. (2016) | 2016 | Multi-Country | Multiple Regions | BOTH | Empirical | Analysed national and cross-national patterns of public participation practices in 12 countries, linkages of differences to institutional features of the countries |
| Smailhodzic, Hooijsma, Boonstra, & Langley (2016) | 2016 | N/A | N/A | N/A | Empirical & conceptual | Overviewed literature on the role of social media use on the patients-healthcare professional relationship |
| Smith (2017) | 2017 | USA | America | N/A | Conceptual | Presented a community engagement framework (CEF) for assessment and planning in South Carolina; conceptualized through the experiences of public health practice and academic partners . |
| Solbjor, Rise, Westerlund, & Steinsbekk (2013) | 2013 | Norway | Europe | HIC | Empirical | Explored mental health service users’ and providers’ views on patient participation during episodes of mental illness. |
| Solon et al. (2009) | 2009 | United Kingdom | Europe | HIC | Empirical | Described the development of Q* (performance measurement tool) implemented in hospitals in the Philippines and how it compares with standard performance measures |
| Sorensen & Iedema (2008) | 2008 | Australia | Oceana | HIC | Empirical | Assessed medical decision-making processes, specifically how managers negotiate patient care decisions within high-pressure, high-cost, high-technology environments |
| Souliotis (2015) | 2015 | N/A | N/A | N/A | Conceptual | Explained the role of patient and public involvement in quality of care |
| Souliotis (2016) | 2016 | N/A | N/A | N/A | Conceptual | States the case for patient participation and patient-centred care |
| South & Phillips (2014) | 2014 | No | N/A | N/A | Conceptual | Offers a debate on the community engagement in the public health system as complex systems. Argues community participation is as an essential element of the public health system |
| Southwick, Cranley, & Hallisy (2015) | 2015 | USA | America | HIC | Empirical | Described the development of a patient-initiated voluntary online survey for adverse medical events and presented the findings of this nationwide survey of injured patients and families. |
| Sri B, Sarojini, & Khanna (2012) | 2012 | India | Asia | LMIC | Empirical | Described an investigation into high rates of maternal deaths following public protests in a tribal district of Madhya Pradesh; the findings were presented to state health officials as a means of prevention. |
| Srivastava, Gope, et al. (2016) | 2016 | India | Asia | LMIC | Empirical | Explored level and types of linkages between public health sector and NGOs in Uttar Pradesh (UP), an underprivileged state of India |
| Srivastava, Bhattacharyya, Gautham, Schellenberg, & Avan (2016) | 2016 | India | Asia | LMIC | Empirical | Explored village health sanitation and nutrition committees (VHSNCs) as enablers of participatory action for community health in two rural districts in two states of eastern India – West Singhbhum in Jharkhand and Kendujhar, in Odisha |
| Stepurko, Pavlova, & Groot (2016) | 2016 | Multi-Country | Multiple Regions | BOTH | Empirical | Examined the relationship between user satisfaction of services and the quality of and access to health care services. |
| Stevenson & Sinclair (2018) | 2018 | USA | America | HIC | Empirical | Retrospective analysis of hospice complaint trends and describe state investigation practices in United States (between 2005 and 2015) |
| Stewardson et al. (2016) | 2016 | Switzerland | Europe | HIC | Empirical | Assessed the effect of enhanced performance feedback and patient participation on hand hygiene compliance of health-care workers (cluster randomised controlled trial) |
| {Stewart Williams, 2020 #23152} | 2020 | Multi-Country | Multiple Regions | LMIC | Empirical | Assessed the health system responsiveness toward community-dwelling adults aged 50 and over in China, Ghana, India, the Russian Federation and South Africa (using the WHO SAGE instrument) |
| Street, Duszynski, Krawczyk, & Braunack-Mayer (2014) | 2014 | Multi-Country | Multiple Regions | BOTH | Empirical & conceptual | Reviewed various aspects of citizen juries - overall process, recruitment, evidence presentation, documentation and outputs in empirical studies |
| Subhedar & Parry (2010) | 2010 | England | Europe | HIC | Empirical & conceptual | Review on critical incident reporting processes in neonatal practice |
| Tabrizi et al. (2018) | 2018 | Iran | Asia | LMIC | Empirical | Comparison of health care utilization and WHO responsiveness between slum (Akhmaqaya Slum Region) and non-slum regions (Tabriz Representative Sample) in Iran |
| Tancred et al. (2014) | 2014 | Multi-Country | Africa | LMIC | Empirical | Experiences of implementation of the Expanded Quality Management Using Information Power (EQUIP) (quality improvement intervention for improving maternal and new-born health) at the community level |
| Taylor, Wolfe, & Cameron (2004) | 2004 | Australia | Oceana | HIC | Empirical | Analysis and comparison of patients' complaints by demographic subgroups within 67 Victorian hospitals |
| Taylor, Abbott, & Hardy (2012) | 2012 | England | Europe | HIC | Empirical | Evaluation findings of a service user-led initiative (INFORM research project - group of volunteer mental health survivors) |
| Teklehaimanot, Teklehaimanot, Tedella, & Abdella (2016) | 2016 | Ethiopia | Africa | LMIC | Empirical | Development and implementation of a balanced scorecard to measure performance of the Health Extension Program in Ethiopia |
| Teno et al. (2018) | 2018 | USA | America | HIC | Conceptual | Methodological discussion on accountability assessment for community-based programs for the seriously ill, America |
| Thornton et al. (2017) | 2017 | USA | America | HIC | Empirical | Examined patient satisfaction and factors that influence it in primary care settings |
| Thurston, Robinson Vollman, Meadows, & Rutherford (2005) | 2005 | Canada | America | HIC | Empirical | Presents a theoretical framework for understanding public participation in the context of regionalized health governance; five case studies of public participation initiatives in Canada |
| Thurston et al. (2005) | 2005 | Canada | America | HIC | Empirical & conceptual | A case study of the partnership between Calgary Health Region and Salvation Army as a participation mechanism for women's health |
| Tierney, McEvoy, Hannigan, & MacFarlane (2018) | 2018 | Ireland | Europe | HIC | Empirical | Empirical analysis of the implementation of community participation i.e. primary care teams in Ireland |
| Tighe, Woloshynowych, Brown, Wears, & Vincent (2006) | 2006 | United Kingdom | Europe | HIC | Empirical | Evaluation of internal incident reporting processes and availability of information in an accident and emergency department in a teaching hospital in London |
| Tille et al. (2019) | 2019 | Germany | Europe | LMIC | Empirical | Measured overall levels of health system responsiveness and the associations with social determinants for ambulatory health care in Germany |
| Topp, Black, Morrow, Chipukuma, & Van Damme (2015) | 2015 | Zambia | Africa | LMIC | Empirical | Examined the impact of HIV scale-up on mechanisms of accountability in Zambian primary health facilities |
| Topp, Sharma, et al. (2018) | 2018 | Australia | Oceana | HIC | Empirical | Mapped Aboriginal and Torres Strait Islander Health Workers (AHWs) experiences of accountability in the Australian health system |
| Topp, Edelman, & Taylor (2018) | 2018 | Zambia | Africa | LMIC | Empirical & conceptual | Evaluated the impact of eight Prison Health committees 18 months after programme initiation. |
| Torabipour, Gharacheh, Lorestani, & Salehi (2017) | 2017 | Iran | Asia | LMIC | Empirical | Compared the overall responsiveness level of public and private physiography clinics |
| Tremblay, Roberge, & Berbiche (2015) | 2015 | Canada | America | HIC | Empirical | Measured patients’ perceptions of responsiveness (WHO) of cancer services |
| Trevena et al. (2017) | 2017 | Australia | Oceana | HIC | Conceptual | Described shared decision-making policies and implementation process and approaches in Australia |
| Tripathy, Aggarwal, Patro, & Verma (2015) | 2015 | India | Asia | LMIC | Empirical | Process evaluation of a community monitoring implementation focusing on processes and inputs, and challenges |
| Tritter (2011) | 2011 | Multi-Country | Europe | HIC | Conceptual | Described public and patient participation in health care and health policy in the United Kingdom |
| Turney & Reynard (2014) | 2014 | England | Europe | HIC | Empirical | Evaluated a novel electronic patient feedback method in an outpatient lithotripsy service setting in United Kingdom |
| Tursunbayeva, Franco, & Pagliari (2017) | 2017 | Multi-Country | Multiple Regions | BOTH | Empirical & conceptual | Synthesise literature on how public health sector organizations use social media for e-governance |
| Twiddy et al. (2018) | 2018 | England | Europe | HIC | Empirical | Explored patients’ experience (feedback) of Outpatient Parenteral Antimicrobial Therapy (OPAT) services in Northern England. |
| Ughasoro, Okanya, Uzochukwu, & Onwujekwe (2017) | 2017 | Nigeria | Africa | LMIC | Empirical | Assessed the level of clients’ perceived responsiveness of tertiary hospitals in the provision of specialist health-care services in Nigeria |
| Ugurluoglu & Celik (2006) | 2006 | Turkey | Asia | LMIC | Empirical | Measured the responsiveness level (WHO) of Turkish health care system |
| Unknown (2003) | 2003 | N/A | N/A | N/A | Methodological | Document the technical consultation to develop concepts and methods for measuring the responsiveness of health systems |
| USAID (2001) | 2001 | N/A | N/A | N/A | Conceptual | A practical guideline for developing participatory monitoring and evaluation systems, a focus on feedback mechanisms. |
| Ustun et al. (2003) | 2003 | Multi-Country | Multiple Regions | BOTH | Methodological | Presents the WHO Multi-Country Survey Study on Health and Responsiveness 2000–2001 including health system responsiveness, household health care expenditures and health state valuations. |
| Uzochukwu et al. (2018) | 2018 | Nigeria | Africa | LMIC | Empirical | Explored the governance and accountability readiness of the different layers of implementation of the Basic Healthcare Provision Fund, (BHCPF) how it influences the generation of policy implementation guidelines around governance and accountability for the Fund. |
| Uzochukwu (2011) | 2011 | Nigeria | Africa | LMIC | Empirical | Explored process and functioning factors of Health Facility Committees as well as to trace the effects and impacts of Committees on health service delivery. |
| Uzwiak & Curran (2016) | 2016 | Belize | America | HIC | Empirical | Explored the multisectoral collaboration and its impact on PHC providers and the impact of health reform on (public) participation |
| Valentine, Darby, & Bonsel (2008) | 2008 | Multi-Country | Multiple Regions | BOTH | Empirical | Presents variations of importance of responsiveness domains by country level variables (country of residence, human development, health system expenditure, and ‘‘geographic zones’’) and by subpopulations defined by sex, age, education, health status, and utilization. |
| Valentine, de Silva, & Murray (2000) | 2000 | Multi-Country | Multiple Regions | BOTH | Empirical & conceptual | Methodology and results on measuring health system responsiveness (overall and distribution) for 191 Countries |
| Valentine, Verdes-Tennant, & Bonsel (2015) | 2015 | Multi-Country | Multiple Regions | BOTH | Empirical | Described an approach to study individual-level factors influencing respondents' reporting behaviour for responsiveness using vignettes. |
| Valentine & Bonsel (2016) | 2016 | Multi-Country | Multiple Regions | BOTH | Empirical | Explored associations between health outcomes, health service coverage, health determinants and responsiveness (WHO) |
| Valentine, Bonsel, & Murray (2007) | 2007 | Multi-Country | Multiple Regions | BOTH | Empirical | Psychometric properties health systems responsiveness (WHO) instrument for 70 population (WHO Multi-Country Study) |
| Valentine, Amala de Silva, et al. (2003) | 2003 | N/A | N/A | N/A | Conceptual | Presents the WHO Health System Responsiveness conceptualisation and methodology for measuring the concept |
| Valentine, Lavallée, Bao, Bonsel, & Murray (2003) | 2003 | N/A | N/A | N/A | Methodological | Presents the psychometric properties of health system responsiveness (WHO) data collection instrument (used in the Multi-country Survey Study) |
| Valentine, Juan Pablo Ortiz, et al. (2003) | 2003 | Multi-Country | Europe | BOTH | Empirical & conceptual | Examined health system responsiveness in service settings for outpatient and inpatient in 16 OECD countries (based on multi-country sample survey) |
| Valentine et al. (2003) | 2003 | Multi-Country | Multiple Regions | BOTH | Empirical | Presents weightings of responsiveness domains (based on multi-country sample survey) for 65 countries |
| Van Belle & Mayhew (2016b) | 2016 | N/A | N/A | N/A | Empirical & conceptual | Narrative review of public accountability from non-health disciplines |
| Van Belle & Mayhew (2016a) | 2016 | Ghana | Africa | LMIC | Empirical | Assessed the governance arrangements and the accountability practices of key health actors at district level in Ghana |
| van der Kooy et al. (2017) | 2017 | Netherlands | Europe | HIC | Empirical | Measured responsiveness of perinatal services using a newly developed instrument -Responsiveness in Perinatal and Obstetric Health Care Questionnaire (ReproQ) |
| van der Kooy et al. (2014) | 2014 | Netherlands | Europe | HIC | Empirical | Assessed the psychometric properties of the Responsiveness in Perinatal and Obstetric Health Care Questionnaire (ReproQ)- an adaptation of WHO responsiveness instrument in for Dutch women. |
| Van der Stuyft & Unger (2000) | 2000 | N/A | N/A | N/A | Conceptual | Discussion of the World Health Report methodology and ideological discourses |
| van Oenen et al. (2016) | 2016 | Amsterdam | Europe | HIC | Empirical | Measured the efficacy of an immediate feedback instrument in emergency psychiatry treatment |
| van Velthoven, Atherton, & Powell (2018) | 2018 |  | Europe | HIC | Empirical | Identified the self-reported behaviour of the public in reading and writing online feedback in relation to health services. |
| {Vandan, 2020 #23151} | 2020 | Hong Kong | Asia | HIC | Empirical | A comparison of health system responsiveness toward South Asian ethnic minority people and with local Chinese people in Hong Kong |
| Vanzetta et al. (2014) | 2014 | Italy | Europe | HIC | Empirical | Ascertained how many local health authorities (Aziende Sanitarie Locali, ASL) and public hospitals have a presence on the most widely used social media websites and how public hospitals engage with the general public |
| Veillard et al. (2010) | 2010 | Canada | America | HIC | Empirical | Examined a stewardship and management intervention (a health system strategy map and a strategy-based scorecard) and associations with accountability agreements |
| Velonis et al. (2018) | 2018 | Canada | America | HIC | Empirical | Presents the findings of a rapid needs’ assessment, using concept mapping to engage communities. |
| Vincent (2004) | 2004 | London | Europe | HIC | Conceptual | Describe methodological techniques for analysing clinical incidents i.e. prospective and retrospective approaches |
| Vrbnjak, Denieffe, O’Gorman, & Pajnkihar (2016) | 2016 | N/A | N/A | N/A | Empirical & conceptual | A review on barriers to reporting medication errors and near misses among burses in hospital settings |
| Wada et al. (2011) | 2011 | Japan | Asia | HIC | Empirical | Examined associations between sociodemographic characteristics and preferences for health system goals (including overall responsiveness and responsiveness inequality) |
| Wagner et al. (2016) | 2016 | Amsterdam | Europe | HIC | Empirical | Explored typology and causes of patient safety incidents in hospital units in Netherlands |
| Wahedi, Flores, Beiersmann, Bozorgmehr, & Jahn (2018) | 2018 | Guatemala | America | LMIC | Empirical | Evaluated an online platform to engage citizens in the reporting of health care deficiencies in Guatemala |
| Waldman, Theobald, & Morgan (2018) | 2018 | N/A | N/A | LMIC | Conceptual | Examined accountability and gender in health systems in LMICs focusing on power and inequities; intersectionality and empowerment |
| Wallack (2000) | 2000 | Amsterdam | Europe | HIC | Empirical & conceptual | Assessed the governance arrangements and accountability practices of key health actors at the level of a Ghanaian health district with the aim to understand how far public accountability is achieved. |
| Wallcraft (2012) | 2012 | N/A | N/A | N/A | Conceptual | Conceptual discussion on collaborations between health professionals, patients and users, their families and friends for psychiatric and mental health services |
| Ward, Pinkney, & Fry (2016) | 2016 | England | Europe | HIC | Empirical | Developed a framework for collecting and using user feedback to improve integrated health and social care (England). |
| Weale (2016) | 2016 | N/A | N/A | N/A | Conceptual | A theoretical paper that discusses the consensual and the agonistic theories of public participation and priority setting |
| Weir, d'Entremont, Stalker, Kurji, & Robinson (2009) | 2009 | Canada | Oceana | HIC | Conceptual | Implementation processes (deliberations and decisions) of balanced scorecard to local public health performance measurement |
| Whitney, Easter, & Tchanturia (2008) | 2008 | England | Europe | HIC | Empirical | Examined service users’ experiences of participating in Cognitive Remediation Therapy. |
| Williamson (2014) | 2014 | N/A | N/A | N/A | Conceptual | Conceptual argument for improved ethical support for patient and citizen participation in health |
| Willig et al. (2013) | 2013 | Birmingham | America | HIC | Empirical | Developed a patient follow-up feedback system (automated interactive voice response system IVRS) in conjunction with use of pre-existing electronic medical records (EMRs) |
| Wilson, Yepuri, & Moses (2016) | 2016 | N/A | N/A | N/A | Conceptual | Conceptual paper on benefits and challenges of measuring patient experience in outpatient clinical care |
| Witvliet, Stronks, Kunst, Mahapatra, & Arah (2015) | 2015 | Multi-Country | Multiple Regions | BOTH | Empirical | Explored the link between political factors and patients’ reports of health system responsiveness, multilevel analysis from 44 countries |
| Woollard et al. (2016) | 2016 | N/A | N/A | N/A | Conceptual | A conceptual paper on social accountability at the meso level (broader community and geographic context) |
| World Bank (2011) | 2011 | South Africa | Africa | LMIC | Empirical | Report on accountability in public services (including health) in South Africa |
| World Health Organization (2000b) | 2000 | N/A | N/A | N/A | Empirical | World health report - delineating the measurement of health systems performance and presents the goals of a system (good health, responsiveness and fairness of financial contribution). |
| World Health Organization (2000a) | 2000 | Multi-Country | Multiple Regions | BOTH | Empirical | Report described how overall health system achievement (including responsiveness) was measured for the 191 member countries. Data is drawn from the Key Informant Opinion Survey. |
| World Health Organization (2003) | 2003 | Multi-Country | Multiple Regions | BOTH | Empirical & conceptual | Present findings from key informants' surveys on health system responsiveness |
| World Health Organization (2005) | 2005 | N/A | N/A | N/A | Methodological | The WHO responsiveness analytical guidelines for surveys in the multi-country survey study |
| World Health Organization (2011) | 2011 | N/A | N/A | N/A | Conceptual | Intersectoral action guideline for policymakers to implement effective and sustainable action |
| World Health Organization (2015) | 2015 | Multi-Country | Multiple Regions | BOTH | Empirical | Report on accountability initiatives to increase women and child health indicators |
| World Health Organization (2016) | 2016 | N/A | N/A | N/A | Conceptual | WHO framework for integrated people-centred health services for equal access to quality health services that are co-produced |
| Wouters, Heunis, van Rensburg, & Meulemans (2008) | 2008 | South Africa | Africa | LMIC | Empirical | Determine the levels of patient satisfaction with services at antiretroviral treatment & association between human resource shortages |
| Wright et al. (2017) | 2017 | England | Europe | HIC | Empirical | Investigated the feasibility and acceptability of real-time feedback in UK general practice. |
| Wu, Li, Xu, Wu, & Zhang (2019) | 2019 | China | Asia | LMIC | Empirical | Developed and implemented a balanced scorecard for Chinese integrated care organization (ICO) in a West China Hospital |
| Yakob & Ncama (2016a) | 2016 | Ethiopia | Africa | LMIC | Empirical | Investigated the level and correlates of client satisfaction with HIV care |
| Yakob & Ncama (2016b) | 2016 | Amsterdam | Europe | HIC | Empirical | Investigate factors associated with perceived access to HIV/AIDS Treatment and care services in Wolaita Zone, responsiveness was one of the indicators |
| Yakob & Ncama (2017) | 2017 | Ethiopia | Africa | LMIC | Empirical | Measured the responsiveness performance (WHO) and correlates of HIV/AIDS treatment and care services in the Wolaita Zone of Ethiopia. |
| Yamin & Lander (2015) | 2015 | N/A | N/A | N/A | Conceptual | Proposed a framework for judiciaries and other actors to enforce health-related rights to improve equity and accountability within health systems |
| Yasobant et al. (2016) | 2016 | India | Asia | LMIC | Empirical | Documented government initiatives for diabetes control and advocacy activities at different level of the health system |
| Yassoub, Hashimi, Awada, & El‐Jardali (2014) | 2014 | Lebanon | Africa | LMIC | Empirical | Assessed the responsiveness (WHO) of primary health care centres (PHCs) to NCD |
| Zakkar (2019) | 2019 | N/A | N/A | N/A | Conceptual | Conceptual classification of patient experience |
| Zalmanovitch & Vashdi (2015) | 2015 | Israel | Asia | HIC | Empirical | Examined the association between individual level factors (i.e. Socio-economic status) and disparities in the public’s experiences with their health-care providers. |
| Ziewitz (2017) | 2017 |  | Europe | HIC | Empirical | Offers conceptual and methodological contributions to obtaining web-based patient feedback |

References

Aiken, L. H., Sermeus, W., Van den Heede, K., Sloane, D. M., Busse, R., McKee, M., . . . Moreno-Casbas, M. T. (2012). Patient safety, satisfaction, and quality of hospital care: cross sectional surveys of nurses and patients in 12 countries in Europe and the United States. *BMJ, 344*, e1717.

Al Dweik, R., Stacey, D., Kohen, D., & Yaya, S. (2017). Factors affecting patient reporting of adverse drug reactions: a systematic review. *Br J Clin Pharmacol, 83*(4), 875-883. doi:10.1111/bcp.13159

Alavi, M., Forouzan, A. S., Moradi-Lakeh, M., Ardakani, M. R. K., Shati, M., Noroozi, M., & Sajjadi, H. (2018). Inequality in responsiveness: A study of comprehensive physical rehabilitation centers in capital of Iran. *Health Services Research And Managerial Epidemiology, 5*, 2333392818789026-2333392818789026. doi:10.1177/2333392818789026

Alavi, M., Khodaie Ardakani, M. R., Moradi-Lakeh, M., Sajjadi, H., Shati, M., Noroozi, M., & Forouzan, A. S. (2018). Responsiveness of physical rehabilitation centers in capital of Iran: Disparities and related determinants in public and private sectors. *Frontiers In Public Health, 6*, 317-317. doi:10.3389/fpubh.2018.00317

Alavi, M., Moradi-Lakeh, M., Setareh Forouzan, A., Sajjadi, H., Shati, M., & Khodaie Ardakani, M. R. (2019). Predictors of poor responsiveness in physical rehabilitation centers in Tehran. *Medical Journal Of The Islamic Republic Of Iran, 33*, 18-18. doi:10.34171/mjiri.33.18

Alhafaji, F. Y., Frederiks, B. J. M., & Legemaate, J. (2011). Concurrence between complaints procedures in the Dutch healthcare system. *European Journal of Health Law, 18*(2), 127-148. doi:10.1163/157180911X564598

Ali, F. M. H., Nikoloski, Z., & Reka, H. (2015). Satisfaction and responsiveness with health-care services in Qatar -evidence from a survey. *Health Policy, 119*(11), 1499-1505. doi:10.1016/j.healthpol.2015.09.012

Anderson, B. (2019). Reflecting on the communication process in health care. Part 2: the management of complaints. *British Journal of Nursing, 28*(14), 927-929. doi:10.12968/bjon.2019.28.14.927

Anderson, C., Blenkinsopp, A., & Armstrong, M. (2004). Feedback from community pharmacy users on the contribution of community pharmacy to improving the public's health: A systematic review of the peer reviewed and non-peer reviewed literature 1990-2002. *Health Expectations: An International Journal of Public Participation in Health Care & Health Policy, 7*(3), 191-202. doi:10.1111/j.1369-7625.2004.00274.x

Anderson, E., Shepherd, M., & Salisbury, C. (2006). 'Taking off the suit': Engaging the community in primary health care decision-making. *Health Expectations: An International Journal of Public Participation in Health Care & Health Policy, 9*(1), 70-80. doi:10.1111/j.1369-7625.2006.00364.x

Andersson, N., Matthis, J., Paredes, S., & Ngxowa, N. (2004). Social audit of provincial health services: building the community voice into planning in South Africa. *J Interprof Care, 18*(4), 381-390.

Andrews, M. L., Sánchez, V., Carrillo, C., Allen-Ananins, B., & Cruz, Y. B. (2014). Using a participatory evaluation design to create an online data collection and monitoring system for New Mexico's Community Health Councils. *Evaluation and Program Planning, 42*, 32-42. doi:10.1016/j.evalprogplan.2013.09.003

Antheunis, M. L., Tates, K., & Nieboer, T. E. (2013). Patients’ and health professionals’ use of social media in health care: Motives, barriers and expectations. *Patient education and counseling, 92*(3), 426-431. doi:10.1016/j.pec.2013.06.020

Askari, R., Arab, M., Rashidian, A., Akbari-Sari, A., Hosseini, S. M., & Gharaee, H. (2016). Designing Iranian model to assess the level of health system responsiveness. *Iranian Red Crescent Medical Journal, 18*(3), e24527-e24527. doi:10.5812/ircmj.24527

Asprey, A., Campbell, J. L., Newbould, J., Cohn, S., Carter, M., Davey, A., & Roland, M. (2013). Challenges to the credibility of patient feedback in primary healthcare settings: a qualitative study. *Br J Gen Pract, 63*(608), e200-208. doi:10.3399/bjgp13X664252

Aston, M., Meagher-Stewart, D., Edwards, N., & Young, L. M. (2009). Public health nurses' primary health care practice: Strategies for fostering citizen participation. *Journal of Community Health Nursing, 26*(1), 24-34. doi:10.1080/07370010802605762

Atela, M., Bakibinga, P., Ettarh, R., Kyobutungi, C., & Cohn, S. (2015). Strengthening health system governance using health facility service charters: A mixed methods assessment of community experiences and perceptions in a district in Kenya. *BMC Health Serv Res, 15*, 539. doi:10.1186/s12913-015-1204-6

Atela, M. H. (2013). *Health system accountability and primary health care delivery in rural Kenya. An analysis of the structures, PROCESS, and outcomes.* University of Cambridge,

Atherton, H., Fleming, J., Williams, V., & Powell, J. (2019). Online patient feedback: a cross-sectional survey of the attitudes and experiences of United Kingdom health care professionals. *Journal of health services research & policy, 24*(4), 235-244. doi:10.1177/1355819619844540

Awoke, M. A., Negin, J., Moller, J., Farell, P., Yawson, A. E., Biritwum, R. B., & Kowal, P. (2017). Predictors of public and private healthcare utilization and associated health system responsiveness among older adults in Ghana. *Glob Health Action, 10*(1), 1301723-1301723. doi:10.1080/16549716.2017.1301723

Babu, B., Sharma, Y., Kusuma, Y., Sivakami, M., Lal, D., Marimuthu, P., . . . Sudhakar, G. (2019). Patient experiences and health system responsiveness among internal migrants: A nationwide study in 13 Indian cities. *Journal Of Healthcare Quality Research, 34*(4), 167-175.

Baharvand, P. (2019). Responsiveness of the health system towards patients admitted to west of Iran hospitals. *Electronic Journal of General Medicine, 16*(2), 1-7. doi:10.29333/ejgm/93481

Baim-Lance, A., Tietz, D., Lever, H., Swart, M., & Agins, B. (2019). Everyday and unavoidable coproduction: exploring patient participation in the delivery of healthcare services. *Sociol Health Illn, 41*(1), 128-142. doi:10.1111/1467-9566.12801

Baines, R. L., & Regan de Bere, S. (2018). Optimizing patient and public involvement (PPI): Identifying its 'essential' and 'desirable' principles using a systematic review and modified delphi methodology. *Health Expectations: An International Journal of Public Participation in Health Care & Health Policy, 21*(1), 327-335. doi:10.1111/hex.12618

Baldie, D. J., Guthrie, B., Entwistle, V., & Kroll, T. (2018). Exploring the impact and use of patients' feedback about their care experiences in general practice settings-a realist synthesis. *Fam Pract, 35*(1), 13-21. doi:10.1093/fampra/cmx067

Balestra, G. L., Dasgupta, J., Sandhya, Y. K., & Mannell, J. (2018). Developing political capabilities with Community-Based Monitoring for health accountability: The case of the Mahila Swasthya Adhikar Manch. *Global Public Health, 13*(12), 1853-1864. doi:10.1080/17441692.2018.1464586

Banfield, M., Gardner, K., McRae, I., Gillespie, J., Wells, R., & Yen, L. (2013). Unlocking information for coordination of care in Australia: a qualitative study of information continuity in four primary health care models. *BMC Family Practice, 14*(1), 34-44. doi:10.1186/1471-2296-14-34

Banka, G., Edgington, S., Kyulo, N., Padilla, T., Mosley, V., Afsarmanesh, N., . . . Ong, M. K. (2015). Improving patient satisfaction through physician education, feedback, and incentives. *J Hosp Med, 10*(8), 497-502. doi:10.1002/jhm.2373

Barg, C. J., Miller, F. A., Hayeems, R. Z., Bombard, Y., Cressman, C., & Painter-Main, M. (2017). What's involved with wanting to be involved? Comparing expectations for public engagement in health policy across research and care contexts. *Healthc Policy, 13*(2), 40-56.

Barker, M., & Klopper, H. (2007). Community participation in primary health care projects of the Muldersdrift Health and Development Programme. *Curationis, 30*(2), 36-47. doi:10.4102/curationis.v30i2.1070

Barry, H. E., Campbell, J. L., Asprey, A., & Richards, S. H. (2016). The use of patient experience survey data by out-of-hours primary care services: a qualitative interview study. *BMJ Qual Saf, 25*(11), 851-859. doi:10.1136/bmjqs-2015-003963

Basu, S., Andrews, J., Kishore, S., Panjabi, R., & Stuckler, D. (2012). Comparative Performance of Private and Public Healthcare Systems in Low- and Middle-Income Countries: A Systematic Review. *PLoS medicine, 9*(6), 1-14. doi:10.1371/journal.pmed.1001244

Bauhoff, S., Hotchkiss, D. R., & Smith, O. (2011). Responsiveness and satisfaction with providers and carriers in a safety net insurance program: Evidence from Georgia's Medical Insurance for the Poor. *Health Policy, 102*(2-3), 286-294.

Bauhoff, S., Tkacheva, O., Rabinovich, L., & Bogdan, O. (2016). Developing citizen report cards for primary care: Evidence from qualitative research in rural Tajikistan. *Health Policy and Planning, 31*(2), 259-266. doi:10.1093/heapol/czv052

Bazemore, A., Phillips, R. L., & Miyoshi, T. (2010). Harnessing Geographic Information Systems (GIS) to enable community-oriented primary care. *J Am Board Fam Med, 23*(1), 22-31. doi:10.3122/jabfm.2010.01.090097

Bazzaz, M. M., Taghvaee, M. R. E., Salehi, M., Bakhtiari, M., & Shaye, Z. A. (2015). Health system's responsiveness of inpatients: Hospitals of Iran. *Global Journal Of Health Science, 7*(7 Spec No), 106-113. doi:10.5539/gjhs.v7n7p106

Béhague, D. P., Kanhonou, L. G., Filippi, V., Lègonou, S., & Ronsmans, C. (2008). Pierre Bourdieu and transformative agency: A study of how patients in Benin negotiate blame and accountability in the context of severe obstetric events. *Sociology of health & illness, 30*(4), 489-510. doi:10.1111/j.1467-9566.2007.01070.x

Behdjat, H., Rifkin, S. B., Tarin, E., & Sheikh, M. R. (2009). A new role for Women Health Volunteers in urban Islamic Republic of Iran. *East Mediterr Health J, 15*(5), 1164-1173.

Belela‐Anacleto, A. S. C., & Pedreira, M. L. G. (2016). Patient safety era: time to think about accountability. In *Nursing in Critical Care* (Vol. 21, pp. 321-322). Malden, Massachusetts: Wiley-Blackwell.

Bell, S. K., Delbanco, T., Anderson-Shaw, L., McDonald, T. B., Gallagher, T. H., Bell, S. K., . . . Gallagher, T. H. (2011). Accountability for medical error: moving beyond blame to advocacy. *CHEST, 140*(2), 519-526. doi:10.1378/chest.10-2533

Benn, J., Koutantji, M., Wallace, L., Spurgeon, P., Rejman, M., Healey, A., & Vincent, A. (2009). Feedback from incident reporting: Information and action to improve patient safety. *Quality and Safety Health Care, 18*(1), 1121. Retrieved from <http://qshc.bmj.com/content/18/1/11.abstract>

Bennetts, W., Cross, W., & Bloomer, M. (2011). Understanding consumer participation in mental health: Issues of power and change. *Int J Ment Health Nurs, 20*(3), 155-164. doi:10.1111/j.1447-0349.2010.00719.x

Berkin, J. A., Lee, C., Landsberger, E., Chazotte, C., Bernstein, P. S., & Goffman, D. (2016). Scorecard implementation improves identification of postpartum patients at risk for venous thromboembolism. *Journal of Healthcare Risk Management, 36*(1), 8-13. doi:10.1002/jhrm.21229

Berlan, D., & Shiffman, J. (2012). Holding health providers in developing countries accountable to consumers: A synthesis of relevant scholarship. *Health Policy and Planning, 27*(4), 271-280. Retrieved from <http://heapol.oxfordjournals.org/content/27/4/271.full.pdf>

Berner, E. S., Ray, M. N., Panjamapirom, A., Maisiak, R. S., Willig, J. H., English, T. M., . . . Schiff, G. D. (2014). Exploration of an automated approach for receiving patient feedback after outpatient acute care visits. *J Gen Intern Med, 29*(8), 1105-1112. doi:10.1007/s11606-014-2783-3

Berta, W., Laporte, A., & Wodchis, W. P. (2014). Approaches to accountability in long-term care. *Healthcare Policy, 10*(SP), 132.

Biebel, K., Nicholson, J., Williams, V., & Hinden, B. R. (2004). Factors Related to the responsiveness of state mental health authorities to parents with mental illness. *Administration and Policy in Mental Health and Mental Health Services Research, 32*(1), 31-48.

Biehl, J., Socal, M. P., & Amon, J. J. (2016). The Judicialization of Health and the Quest for State Accountability: Evidence from 1,262 Lawsuits for Access to Medicines in Southern Brazil. *Health & Human Rights: An International Journal, 18*(1), 209-220. Retrieved from <http://ezproxy.uct.ac.za/login?url=https://search.ebscohost.com/login.aspx?direct=true&db=sih&AN=116633636&site=ehost-live>

Biondo, P. D., King, S., Minhas, B., Fassbender, K., & Simon, J. E. (2019). How to increase public participation in advance care planning: findings from a World Café to elicit community group perspectives. *BMC Public Health, 19*(1), N.PAG-N.PAG. doi:10.1186/s12889-019-7034-4

Birkeland, S. (2019). Health Care Complaints and Adverse Events as a Means of User Involvement for Quality and Safety Improvement. In *Milbank Quarterly* (Vol. 97, pp. 346-349).

Birks, Y., Aspinal, F., & Bloor, K. (2018). *Understanding the drivers of litigation in health services*. Retrieved from England:

Bjorkman, M., & Svensson, J. (2009). Power to the people: Evidence from a randomized field experiment on community-based monitoring in Uganda. *World Bank Policy Research Working Paper, 124*(2), 735-769.

Björkman Nyqvist, M., de Walque, D., & Svensson, J. (2017). Experimental Evidence on the Long-Run Impact of Community-Based Monitoring. *American Economic Journal: Applied Economics, 9*(1), 33-69. doi:10.1257/app.20150027

Blake, C., Annorbah‐Sarpei, N. A., Bailey, C., Ismaila, Y., Deganus, S., Bosomprah, S., . . . Clark, S. (2016). Scorecards and social accountability for improved maternal and newborn health services: A pilot in the Ashanti and Volta regions of Ghana. *International Journal of Gynecology & Obstetrics, 135*(3), 372-379. doi:10.1016/j.ijgo.2016.10.004

Blanchard, C., Godinot, X., Laureau, C., & Wodon, Q. (2007). *Learning from the extreme poor: Participatory approaches to fostering child health in Madagascar*. Retrieved from Washington:

Bleich, S. N., Ozaltin, E., & Murray, C. J. L. (2009). How does satisfaction with the health-care system relate to patient experience? *Bulletin for the World Health Organization, 87*(4), 271-278. doi:10.2471/blt.07.050401

Blendon, R. J., Kim, M., & Benson, J. M. (2001). The public versus the World Health Organization on health system performance. *Health Affairs (Millwood), 20*(3). doi:10.1377/hlthaff.20.3.10

Blendon, R. J., Schoen, C., DesRoches, C., Osborn, R., & Zapert, K. (2003). Common concerns amid diverse systems: health care experiences in five countries. *Health Affairs, 22*(3), 106-121.

Blignault, I., Aspinall, D., Reay, L., & Hyman, K. (2017). Realisation of a joint consumer engagement strategy in the Nepean Blue Mountains region. *Aust J Prim Health, 23*(6), 531-535. doi:10.1071/py16103

Boiko, O., Campbell, J. L., Elmore, N., Davey, A. F., Roland, M., & Burt, J. (2015). The role of patient experience surveys in quality assurance and improvement: a focus group study in English general practice. *Health Expect, 18*(6), 1982-1994. doi:10.1111/hex.12298

Boivin, A., Dumez, V., Fancott, C., & L'Esperance, A. (2018). Growing a healthy ecosystem for patient and citizen partnerships. *Healthc Q, 21*(Sp), 73-82. doi:10.12927/hcq.2018.25634

Bolam, B. L. (2005). Public participation in tackling health inequalities: implications from recent qualitative research. *Eur J Public Health, 15*(5), 447. doi:10.1093/eurpub/cki174

Bonino, F., & Warner, A. (2014). *What makes humanitarian feedback mechanisms work? Literature review to support an ALNAP–CDA action research into humanitarian feedback mechanisms*. Retrieved from London:

Boogaard, J. A., de Vet, H. C. W., van Soest-Poortvliet, M. C., Anema, J. R., Achterberg, W. P., & van der Steen, J. T. (2018). Effects of two feedback interventions on end-of-life outcomes in nursing home residents with dementia: A cluster-randomized controlled three-armed trial. *Palliat Med, 32*(3), 693-702. doi:10.1177/0269216317750071

Boothroyd, R. I., Flint, A. Y., Lapiz, A. M., Lyons, S., Jarboe, K. L., & Aldridge, W. A. (2017). Active involved community partnerships: Co-creating implementation infrastructure for getting to and sustaining social impact. *Translational Behavioral Medicine, 7*(3), 467-477. doi:10.1007/s13142-017-0503-3

Bottacini, A., Scalia, P., & Goss, C. (2017). Shared decision making in Italy: An updated revision of the current situation. *Z Evid Fortbild Qual Gesundhwes, 123-124*, 61-65. doi:10.1016/j.zefq.2017.05.023

Boucaud, S., & Dorschner, D. (2016). Patient safety incident reporting: current trends and gaps within the Canadian health system. *Healthc Q, 18*(4), 66-71.

Bowyer, A. V., Finlay, I., Baillie, J., Byrne, A., McCarthy, J., Sampson, C., . . . Nelson, A. (2019). Gaining an accurate reflection of the reality of palliative care through the use of free-text feedback in questionnaires: the AFTER study. *BMJ Support Palliat Care, 9*(1), e17. doi:10.1136/bmjspcare-2015-000920

Boydell, V., McMullen, H., Cordero, J., Steyn, P., & Kiare, J. (2019). Studying social accountability in the context of health system strengthening: Innovations and considerations for future work. *Health Research Policy and Systems, 17*(1), 34. doi:10.1186/s12961-019-0438-x

Bradshaw, P. L. (2008). Service user involvement in the NHS in England: genuine user participation or a dogma-driven folly? *J Nurs Manag, 16*(6), 673-681. doi:10.1111/j.1365-2834.2008.00910.x

Bramesfeld, A., Klippel, U., Seidel, G., Schwartz, F. W., & Dierks, M.-L. (2007). How do patients expect the mental health service system to act? Testing the WHO responsiveness concept for its appropriateness in mental health care. *Social Science & Medicine, 65*(5), 880-889. doi:10.1016/j.socscimed.2007.03.056

Bramesfeld, A., & Stegbauer, C. (2016). Assessing the performance of mental health service facilities for meeting patient priorities and health service responsiveness. *Epidemiology and Psychiatric Sciences, 25*(5), 417-421. doi:10.1017/S2045796016000354

Bramesfeld, A., Wedegartner, F., Elgeti, H., & Bisson, S. (2007). How does mental health care perform in respect to service users' expectations? Evaluating inpatient and outpatient care in Germany with the WHO responsiveness concept. *BMC Health Services Research, 7*, 99. doi:10.1186/1472-6963-7-99

Bramesfeld, A., Wensing, M., Bartels, P., Bobzin, H., Grenier, C., Heugren, M., . . . Szecsenyi, J. (2016). Mandatory national quality improvement systems using indicators: An initial assessment in Europe and Israel. *Health Policy, 120*(11), 1256-1269. doi:10.1016/j.healthpol.2016.09.019

Bridges, J., Pope, C., & Braithwaite, J. (2019). Making health care responsive to the needs of older people. *Age And Ageing*. doi:10.1093/ageing/afz085

Brinkerhoff, D. (2003). *Accountability and health systems: Overview,*

*framework, and strategies*. Retrieved from Bethesda, Maryland:

Brinkerhoff, D. W. (2004). Accountability and health systems: Toward conceptual clarity and policy relevance. *Health Policy Plan, 19*(6), 371-379. doi:10.1093/heapol/czh052

Brookes, G., & Baker, P. (2017). What does patient feedback reveal about the NHS? A mixed methods study of comments posted to the NHS Choices online service. *BMJ Open, 7*(4), e013821. doi:10.1136/bmjopen-2016-013821

Brown, A., Ford, T., Deighton, J., & Wolpert, M. (2014). Satisfaction in child and adolescent mental health services: translating users' feedback into measurement. *Adm Policy Ment Health, 41*(4), 434-446. doi:10.1007/s10488-012-0433-9

Brown, J., Rafferty, J., Golding, D., Adewale, V., Chan, L., Pastorello, C., . . . Neukirch, J. (2016). Patient engagement for youth in multiple facets of healthcare in Rhode Island. *Rhode Island Medical Journal, 99*(8), 19-21. Retrieved from <http://ezproxy.uct.ac.za/login?url=https://search.ebscohost.com/login.aspx?direct=true&db=aph&AN=117311855&site=ehost-live>

Bryant, J., Saxton, M., Madden, A., Bath, N., & Robinson, S. (2008). Consumer participation in the planning and delivery of drug treatment services: the current arrangements. *Drug Alcohol Rev, 27*(2), 130-137. doi:10.1080/09595230701829397

Bunton, R. (2008). Public health and public involvement. *Critical Public Health, 18*(2), 131-134. doi:10.1080/09581590802166627

Busse, R. (2013). Understanding satisfaction, responsiveness and experience with the health system. In I. Papanicolas & P. Smith (Eds.), *Health System Performance Comparison: An Agenda for Policy, Information and Research* (pp. 255-279). New York: McGraw-Hill Education.

Busse, R., Valentine, N., S, L., Prasad, A., & Van Ginneken, E. (2012). Being responsive to citizens’ expectations: The role of health services in responsiveness and satisfaction. In J. Figueras & M. McKee (Eds.), *Health systems, health, wealth and societal well-being: assessing the case for investing in health systems* (pp. 175-208). Maidenhead: McGraw-Hill Education.

Byrkjeflot, H., Neby, S., & Vrangbæk, K. (2012). Changing accountability regimes in hospital governance: Denmark and Norway compared. *Scandinavian Journal of Public Administration, 15*(4), 3-23.

Byskov, J., Marchal, B., Maluka, S., Zulu, J. M., Bukachi, S. A., Hurtig, A.-K., . . . Olsen, O. E. (2014). The accountability for reasonableness approach to guide priority setting in health systems within limited resources: findings from action research at district level in Kenya, Tanzania, and Zambia. In (Vol. 12, pp. 49-49).

Callaghan, G. D., & Wistow, G. (2006). Publics, patients, citizens, consumers? Power and decision making in primary health care. *Public Administration, 84*(3), 583-601. doi:10.1111/j.1467-9299.2006.00603.x

Campbell, J., Narayanan, A., Burford, B., & Greco, M. (2010). Validation of a multi-source feedback tool for use in general practice. *Educ Prim Care, 21*(3), 165-179.

Cervia, S. (2018). Citizen engagement and the challenge of democratizing health: an Italian case study. *L'engagement des citoyens et le challenge de la démocratisation de la santé: une étude de cas italien.*(117), 145-165. Retrieved from <http://ezproxy.uct.ac.za/login?url=https://search.ebscohost.com/login.aspx?direct=true&db=sih&AN=133419124&site=ehost-live>

Chand Chauhan, R., Jacob Purty, A., & Singh, Z. (2015). Notified or missed cases? An assessment of successful linkage for referred tuberculosis patients in South India. *Community Acquired Infection, 2*(4), 137-141. doi:10.4103/2225-6482.172652

Chao, J., Lu, B., Zhang, H., Zhu, L., Jin, H., & Liu, P. (2017). Healthcare system responsiveness in Jiangsu Province, China. *BMC Health Services Research, 17*(1), 31-31. doi:10.1186/s12913-017-1980-2

Charles, A., Cross, W., & Griffiths, D. (2017). What do clinicians understand about deaths reportable to the Coroner? *Journal of forensic and legal medicine, 51*, 76-80. Retrieved from <http://ezproxy.uct.ac.za/login?url=https://search.ebscohost.com/login.aspx?direct=true&db=awn&AN=28763711&site=ehost-live>

Checkland, K., Marshall, M., & Harrison, S. (2004). Re-thinking accountability: trust versus confidence in medical practice. *Qual Saf Health Care, 13*(2), 130-135. doi:10.1136/qshc.2003.009720

Cheng, B. S., McGrath, C., Bridges, S. M., & Yiu, C. K. (2015). Development and evaluation of a Dental Patient Feedback on Consultation skills (DPFC) measure to enhance communication. *Community Dent Health, 32*(4), 226-230.

Cheraghi-Sohi, S., & Bower, P. (2008). Can the feedback of patient assessments, brief training, or their combination, improve the interpersonal skills of primary care physicians? A systematic review. *BMC Health Serv Res, 8*, 179. doi:10.1186/1472-6963-8-179

Chew‐Graham, C. (2016). Positive reporting? Is there a bias is reporting of patient and public involvement and engagement? *Health Expectations: An International Journal of Public Participation in Health Care & Health Policy, 19*(3), 499-500. doi:10.1111/hex.12470

Chimbindi, N., Barnighausen, T., & Newell, M.-L. (2014). Patient satisfaction with HIV and TB treatment in a public programme in rural KwaZulu-Natal: Evidence from patient-exit interviews. *BMC Health Services Research, 14*(32), 32. doi:10.1186/1472-6963-14-32

Chretien, K. C., & Kind, T. (2013). Social media and clinical care: ethical, professional, and social implications. *Circulation, 127*(13), 1413-1421. doi:10.1161/circulationaha.112.128017

Christiaans-Dingelhoff, I., Smits, M., Zwaan, L., Lubberding, S., van der Wal, G., & Wagner, C. (2011). To what extent are adverse events found in patient records reported by patients and healthcare professionals via complaints, claims and incident reports? *BMC Health Services Research, 11*(1), 49-58. doi:10.1186/1472-6963-11-49

Chuengsatiansup, K., Tengrang, K., Posayanonda, T., & Sihapark, S. (2019). Citizens' jury and elder care: public participation and deliberation in long-term care policy in Thailand. *Journal of Aging & Social Policy, 31*(4), 378-392. doi:10.1080/08959420.2018.1442110

Cleary, S. M., Molyneux, S., & Gilson, L. (2013). Resources, attitudes and culture: An understanding of the factors that influence the functioning of accountability mechanisms in primary health care settings. *BMC Health Services Research, 13*, 320. doi:10.1186/1472-6963-13-320

Cleary, S. M., Schaay, N., Botes, E., FIglan, N., Lehmann, U., & Gilson, L. (2014). Re-imagining community participation at the district level: Lessons from the DIALHS collaboration. *South African Health Review, 2014/15*, 151-162.

Cleopas, A., Villaveces, A., Charvet, A., Bovier, P., Kolly, V., & Perneger, T. (2006). Patient assessments of a hypothetical medical error: effects of health outcome, disclosure, and staff responsiveness. *BMJ Quality & Safety, 15*(2), 136-141.

Coates, D. (2016). Client and parent feedback on a youth mental health service: The importance of family inclusive practice and working with client preferences. *Int J Ment Health Nurs, 25*(6), 526-535. doi:10.1111/inm.12240

Colombo, C., & Mosconi, P. (2016). Citizens' juries could help to guide screening policy. In *BMJ* (2016/06/30 ed., Vol. 353, pp. i3520).

Condon, L. (2017). Seeking the views of service users: From impossibility to necessity. *Health Expectations: An International Journal of Public Participation in Health Care & Health Policy, 20*(5), 805-806. doi:10.1111/hex.12621

Contandriopoulos, D. (2004). A sociological perspective on public participation in health care. *Social Science & Medicine, 58*(2), 321. doi:10.1016/S0277-9536(03)00164-3

Contandriopoulos, D., Denis, J.-L., & Langley, A. (2004). Defining the `public' in a public healthcare system. *Human Relations, 57*(12), 1573-1596. doi:10.1177/0018726704049990

Cordery, C., Baskerville, R., & Porter, B. (2010). Control or collaboration? Contrasting accountability relationships in the primary health sector. *Accounting, Auditing & Accountability Journal, 23*(6), 793-813.

Cordery, C. J. (2008). *Dimensions of accountability: Voices from New Zealand primary health organisations.* Victoria University of Wellington,

Cornwall, A., Cordeiro, S., & Delgado, N. G. (2006). Rights to health and struggles for accountability in a Brazilian municipal health council. In *Righs, Resources and the Politics and Accountability.* London: Zed books.

Cornwall, A., Lucas, H., & Pasteur, K. (2000). Introduction: Accountability through participation: developing workable partnership models in the health sector. *IDS Bulletin, 31*(1), 1-13.

Coulter, A., & Jenkinson, C. (2005). European patients' views on the responsiveness of health systems and healthcare providers. *Eur J Public Health, 15*(4), 355-360. doi:10.1093/eurpub/cki004

Covell, N. H., Donahue, S. A., Ulaszek, W. R., Dunakin, L., Essock, S. M., & Felton, C. J. (2006). Effectiveness of Two Methods of Obtaining Feedback on Mental Health Services Provided to Anonymous Recipients. *Psychiatric Services, 57*(9), 1324-1327. doi:10.1176/appi.ps.57.9.1324

Crawford, M. J., Aldridge, T., Bhui, K., Rutter, D., Manley, C., Weaver, T., . . . Fulop, N. (2003). User involvement in the planning and delivery of mental health services: A cross-sectional survey of service users and providers. *Acta Psychiatr Scand, 107*(6), 410-414.

Crawford, M. J., Rutter, D., Manley, C., Weaver, T., Bhui, K., Fulop, N., & Tyrer, P. (2002). Systematic review of involving patients in the planning and development of health care. *BMJ, 325*(7375), 1263. doi:10.1136/bmj.325.7375.1263

Crofts, J., Moyo, J., Ndebele, W., Mhlanga, S., Draycott, T., & Sibanda, T. (2014). Adaptation and implementation of local maternity dashboards in a Zimbabwean hospital to drive clinical improvement. *Adaptation et mise en oeuvre de tableaux de bord de maternité locaux dans un hôpital du Zimbabwe pour stimuler les améliorations cliniques., 92*(2), 146-152. doi:10.2471/BLT.13.124347

Cullinan, K. (2013). Citizen reporting on district health services. In A. Padarath & R. English (Eds.), *South African Health Review* (2012/2013 ed., pp. 83-88). Durban: Health Systems Trust.

Danhoundo, G., Nasiri, K., & Wiktorowicz, M. E. (2018). Improving social accountability processes in the health sector in sub-Saharan Africa: A systematic review. *BMC Public Health, 18*(1), 497-497. doi:10.1186/s12889-018-5407-8

Danovitch, I., & Kan, D. (2017). The Addiction Benefits Scorecard: A Framework to Promote Health Insurer Accountability and Support Consumer Engagement. *Journal of Psychoactive Drugs, 49*(2), 122-131. doi:10.1080/02791072.2017.1296210

Darby, C., Valentine, N., Murray, C. J. L., & de Silva, A. (2000). World Health Organization (WHO): Strategy on measuring responsiveness. In *GPE Discussion Paper Series: No.23*. Geneva: World Health Organization.

Dasgupt, J., Sandhya, Y. K., Lobis, S., Verma, P., & Schaaf, M. (2015). Using technology to claim rights to free maternal health care: Lessons about impact from the My Health, My Voice pilot project in India. *Health and Human Rights, 17*(2), E135-147.

Davidson, K., Perry, A., & Bell, L. (2015). Would continuous feedback of patient's clinical outcomes to practitioners improve NHS psychological therapy services? Critical analysis and assessment of quality of existing studies. *Psychology and Psychotherapy: Theory, Research and Practice, 88*(1), 21-37. doi:10.1111/papt.12032

Davies, J., Wright, J., Drake, S., & Bunting, J. (2009). 'By listening hard': Developing a service-user feedback system for adopted and fostered children in receipt of mental health services. *Adoption & Fostering, 33*(4), 19-33. Retrieved from <http://ezproxy.uct.ac.za/login?url=https://search.ebscohost.com/login.aspx?direct=true&db=cin20&AN=105292633&site=ehost-live>

De Brun, A., Heavey, E., Waring, J., Dawson, P., & Scott, J. (2017). PReSaFe: A model of barriers and facilitators to patients providing feedback on experiences of safety. *Health Expect, 20*(4), 771-778. doi:10.1111/hex.12516

de Freitas, C., & Martin, G. (2015). Inclusive public participation in health: Policy, practice and theoretical contributions to promote the involvement of marginalised groups in healthcare. *Social Science & Medicine, 135*, 31-39. doi:10.1016/j.socscimed.2015.04.019

de Kok, B. C. (2019). Between orchestrated and organic: Accountability for loss and the moral landscape of childbearing in Malawi. *Social Science & Medicine, 220*, 441-449. doi:10.1016/j.socscimed.2018.09.036

de Lara, L., & Guareschi, N. M. (2016). The Forum for Defence of the Brazilian Unified Health System (Sistema Unico de Saude) and its role in building community participation in the fight against the privatization of health. *J Health Psychol, 21*(3), 439-447. doi:10.1177/1359105316628745

de Silva, A. (2000). *A framework for measuring responsiveness*. Retrieved from Geneva: <http://www3.who.int/whosis/discussion_papers/pdf/paper32.pdf>

de Silva, A., & Valentine, N. (2000). Measuring responsiveness: Results of a key informants survey in 35 countries. In *GPE Discussion Paper Series: No.21*. Geneva: World Health Organization.

de Vos, M. S., Hamming, J. F., Cchua-Hendriks, J. J., & Marang-van de Mheen, P. J. (2019). Connecting perspectives on quality and safety: patient-level linkage of incident, adverse event and complaint data. *BMJ Quality & Safety, 28*(3), 180-189. doi:10.1136/bmjqs-2017-007457

De Vos, P., De Ceukelaire, W., Malaise, G., Perez, D., Lefevre, P., & Van der Stuyft, P. (2009). Health through people's empowerment: a rights-based approach to participation. *Health Hum Rights, 11*(1), 23-35.

DeCourcy, A., West, E., & Barron, D. (2012). The National Adult Inpatient Survey conducted in the English National Health Service from 2002 to 2009: how have the data been used and what do we know as a result? *BMC Health Serv Res, 12*, 71. doi:10.1186/1472-6963-12-71

Delgado-Gallego, M. E., & Vazquez, M. L. (2012). Changes in awareness and utilization of social participation mechanisms of the Colombian health care system in the last 10 years. *Int J Health Serv, 42*(4), 695-718. doi:10.2190/HS.42.4.g

Denis, J. L. (2014). Accountability in healthcare organizations and systems. *Healthc Policy, 10*(Spec issue), 8-11.

Desai, D. G., Ndukwu, J. O., & Mitchell, J. P. (2015). Social media in health care: how close is too close? *Health Care Manag (Frederick), 34*(3), 225-233. doi:10.1097/hcm.0000000000000072

Dewi, F. D., Sudjana, G., & Oesman, Y. M. (2011). Patient satisfaction analysis on service quality of dental health care based on empathy and responsiveness. *Dental Research Journal, 8*(4), 172-177. doi:10.4103/1735-3327.86032

Dickson, M., Riddell, H., Gilmour, F., & McCormack, B. (2017). Delivering dignified care: A realist synthesis of evidence that promotes effective listening to and learning from older people's feedback in acute care settings. *Journal of Clinical Nursing (John Wiley & Sons, Inc.), 26*(23-24), 4028-4038. doi:10.1111/jocn.13856

Douangvichit, D., & Liabsuetrakul, T. (2012). Obstetric care and health system responsiveness for hospital-based delivery in Lao People's Democratic Republic. *Journal of the Medical Association of Thailand, 95*(9), 1126.

Douglas, S. R., Jonghyuk, B., de Andrade, A. R. V., Tomlinson, M. M., Hargraves, R. P., & Bickman, L. (2015). Feedback mechanisms of change: How problem alerts reported by youth clients and their caregivers impact clinician-reported session content. *Psychotherapy Research, 25*(6), 678-693. doi:10.1080/10503307.2015.1059966

Dullie, L., Meland, E., Mildestvedt, T., Hetlevik, O., & Gjesdal, S. (2018). Quality of primary care from patients' perspective: a cross sectional study of outpatients' experience in public health facilities in rural Malawi. *BMC Health Serv Res, 18*(1), 872. doi:10.1186/s12913-018-3701-x

Dyer, S. (2004). Rationalising public participation in the health service: the case of research ethics committees. *Health & place, 10*(4), 339-348. doi:10.1016/j.healthplace.2004.08.004

Ebrahim, A. (2003). Accountability in practice: Mechanisms for NGOs. *World Development, 31*(5), 813-829. doi:10.1016/s0305-750x(03)00014-7

Ebrahimipour, H., Vafaei Najjar, A., Khani Jahani, A., Pourtaleb, A., Javadi, M., Rezazadeh, A., . . . Shirdel, A. (2013). Health system responsiveness: A case study of general hospitals in Iran. *International Journal of Health Policy and Management, 1*(1), 85-90. doi:10.15171/ijhpm.2013.13

Edward, A., Kumar, B., Kakar, F., Salehi, A. S., Burnham, G., & Peters, D. H. (2011). Configuring balanced scorecards for measuring health system performance: evidence from 5 years' evaluation in Afghanistan. *PLoS Med, 8*(7), e1001066. doi:10.1371/journal.pmed.1001066

Edward, A., Osei-Bonsu, K., Branchini, C., Yarghal, T. S., Arwal, S. H., & Naeem, A. J. (2015). Enhancing governance and health system accountability for people centered healthcare: An exploratory study of community scorecards in Afghanistan. *BMC Health Services Research, 15*(299). doi:10.1186/s12913-015-0946-5

Eijkholt, M., Jankowski, J., & Fisher, M. (2017). Screen shots: when patients and families publish negative health care narratives online. *Narrat Inq Bioeth, 7*(3), 245-254. doi:10.1353/nib.2017.0072

Ellins, J. O., & Glasby, J. O. N. (2016). “You don't know what you are saying ‘Yes’ and what you are saying ‘No’ to”: hospital experiences of older people from minority ethnic communities. *Ageing & Society, 36*(1), 42-63. doi:10.1017/S0144686X14000919

Emslie, M. J., Andrew, J., Entwistle, V., & Walker, K. (2005). Who are your public? A survey comparing the views of a population-based sample with those of a community-based public forum in Scotland. *Health & Social Care in the Community, 13*(2), 164-169. doi:10.1111/j.1365-2524.2005.00544.x

Entwistle, V. A., Andrew, J. E., Emslie, M. J., Walker, K. A., Dorrian, C., Angus, V. C., & Conniff, A. O. (2003). Public opinion on systems for feeding back views to the National Health Service. *Quality and Safety in Health Care, 12*(6), 435-442. Retrieved from <http://ezproxy.uct.ac.za/login?url=https://search.ebscohost.com/login.aspx?direct=true&db=awn&AN=1169618&site=ehost-live>

Enwere, J. E. N., Keating, E. A., & Weber, R. J. (2014). Balanced Scorecards As a Tool for Developing Patient-Centered Pharmacy Services. *Hospital Pharmacy, 49*(6), 579-584. doi:10.1310/hpj4906-579

Eriksson, E. M., Raharjo, H., & Gustavsson, S. (2018). Exploring complaints by female and male patients at Swedish hospitals using a probabilistic graphical model. *Scandinavian Journal of Caring Sciences, 32*(3), 1148-1156. doi:10.1111/scs.12560

Evans, A. (2018). Amplifying accountability by benchmarking results at district and national levels. *Development Policy Review, 36*(2), 221-240. doi:10.1111/dpr.12213

Eyre, R., & Gauld, R. (2003). Community participation in a rural community health trust: the case of Lawrence, New Zealand. *Health Promot Int, 18*(3), 189-197. doi:10.1093/heapro/dag014

Fabbri, C., Dutt, V., Shukla, V., Singh, K., Shah, N., & Powell-Jackson, T. (2019). The effect of report cards on the coverage of maternal and neonatal health care: a factorial, cluster-randomised controlled trial in Uttar Pradesh, India. *The Lancet Global Health, 7*(8), e1097-e1108. doi:10.1016/S2214-109X(19)30254-2

Falisse, J. B., Meessen, B., Ndayishimiye, J., & Bossuyt, M. (2012). Community participation and voice mechanisms under performance-based financing schemes in Burundi. *Trop Med Int Health, 17*(5), 674-682. doi:10.1111/j.1365-3156.2012.02973.x

Farmakas, A., Theodorou, M., Galanis, P., Karayiannis, G., Ghobrial, S., Polyzos, N., . . . Souliotis, K. (2017). Public engagement in setting healthcare priorities: a ranking exercise in Cyprus. *Cost Effectiveness & Resource Allocation, 15*, 1-11. doi:10.1186/s12962-017-0078-3

Farmer, J., Bigby, C., Davis, H., Carlisle, K., Kenny, A., & Huysmans, R. (2018). The state of health services partnering with consumers: evidence from an online survey of Australian health services. *BMC Health Serv Res, 18*(1), 628. doi:10.1186/s12913-018-3433-y

Farmer, J., Currie, M., Kenny, A., & Munoz, S.-A. (2015). An exploration of the longer-term impacts of community participation in rural health services design. *Social Science & Medicine, 141*, 64-71. doi:10.1016/j.socscimed.2015.07.021

Farrington, C., Burt, J., Boiko, O., Campbell, J., & Roland, M. (2017). Doctors' engagements with patient experience surveys in primary and secondary care: a qualitative study. *Health Expect, 20*(3), 385-394. doi:10.1111/hex.12465

Fatima, I., Humayun, A., Anwar, M. I., Iftikhar, A., Aslam, M., & Shafiq, M. (2017). How Do Patients Perceive and Expect Quality of Surgery, Diagnostics, and Emergency Services in Tertiary Care Hospitals? An Evidence of Gap Analysis From Pakistan. *Oman Medical Journal, 32*(4), 297-305. doi:10.5001/omj.2017.58

Fazaeli, S., Ahmadi, M., Rashidian, A., & Sadoughi, F. (2014). A framework of a health system responsiveness assessment information system for Iran. *Iran Red Crescent Medical Journal, 16*(6), e17820. doi:10.5812/ircmj.17820

Fazaeli, S., Yousefi, M., Banikazemi, S. H., Ghazizadeh Hashemi, S. A. H., Vakilzadeh, A. K., & Hoseinzadeh Aval, N. (2016). Importance of health system responsiveness in a high and low income areas in Mashhad, Iran: A household survey.

Fekri, O., Macarayan, E. R., & Klazinga, N. (2018). *Health system performance assessment in the WHO European region: Which domains and indicators have been used by member states for its measurement?* Retrieved from <http://ezproxy.uct.ac.za/login?url=https://search.ebscohost.com/login.aspx?direct=true&db=cmedm&AN=30091869&site=ehost-live>

Feruglio, F., & Nisbett, N. (2018). The challenges of institutionalizing community-level social accountability mechanisms for health and nutrition: A qualitative study in Odisha, India. *BMC Health Services Research, 18*(1), 788. doi:10.1186/s12913-018-3600-1

Fiorentini, G., Ragazzi, G., & Robone, S. (2015). Are bad health and pain making us grumpy? An empirical evaluation of reporting heterogeneity in rating health system responsiveness. *Social Science & Medicine, 144*, 48-58. doi:10.1016/j.socscimed.2015.09.009

Fiorentini, G., Robone, S., & Verzulli, R. (2018). Do hospital-specialty characteristics influence health system responsiveness? an empirical evaluation of in-patient care in the Italian region of Emilia-Romagna. *Health Economics, 27*(2), 266-281. Retrieved from <http://ezproxy.uct.ac.za/login?url=https://search.ebscohost.com/login.aspx?direct=true&db=ecn&AN=1716017&site=ehost-live>

Fisher, E. S., & Corrigan, J. (2014). Accountable health communities: Getting there from here. *JAMA: Journal of the American Medical Association, 312*(20), 2093-2094. doi:10.1001/jama.2014.13815

Flores, W., & Hernandez, A. (2018). Health accountability for indigenous populations: Confronting power through adaptive action cycles. In E. Nelson, G. Bloom, & A. Shankland (Eds.), *Accountability for health equity: Galvanising a movement for universal health coverage*: Institute of Development Studies.

Flores, W., & Samuel, J. (2019). Grassroots organisations and the sustainable development goals: no one left behind? *BMJ, 365*, l2269. doi:10.1136/bmj.l2269

Florin, D., & Dixon, J. (2004). Public involvement in health care. *BMJ, 328*(7432), 159-161. doi:10.1136/bmj.328.7432.159

Foran, A., Millar, E., & Dorstyn, D. (2016). Patient satisfaction with a hospital-based neuropsychology service. *Australian Health Review, 40*(4), 447-452. doi:10.1071/AH15054

Forbat, L., Hubbard, G., & Kearney, N. (2009). Patient and public involvement: models and muddles. *Journal of clinical nursing, 18*(18), 2547-2554. doi:10.1111/j.1365-2702.2008.02519.x

Forouzan, A. S. (2015). *Assessing responsiveness in the mental health care system: the case of Tehran.* Umeå universitet,

Forouzan, A. S., Ghazinour, M., Dejman, M., Rafeiey, H., & San Sebastian, M. (2011). Testing the WHO responsiveness concept in the Iranian mental healthcare system: a qualitative study of service users. *BMC Health Services Research, 11*, 325-325. doi:10.1186/1472-6963-11-325

Forouzan, A. S., Rafiey, H., Padyab, M., Ghazinour, M., Dejman, M., & Sebastian, M. S. (2014). Reliability and validity of a mental health system responsiveness questionnaire in Iran. *Glob Health Action, 7*, 24748-24748. doi:10.3402/gha.v7.24748

Forouzan, S., Padyab, M., Rafiey, H., Ghazinour, M., Dejman, M., & San Sebastian, M. (2016). Measuring the mental health-care system responsiveness: Results of an outpatient survey in Tehran. *Front Public Health, 3*, 285. doi:10.3389/fpubh.2015.00285

Foster, F., Piggott, R., Riley, L., & Beech, R. (2016). Working with primary care clinicians and patients to introduce strategies for increasing referrals for pulmonary rehabilitation. *Prim Health Care Res Dev, 17*(3), 226-237. doi:10.1017/s1463423615000286

Foster, T., & Maillardet, V. (2010). Surveying young patients. *Emerg Med J, 27*(3), 221-223. doi:10.1136/emj.2008.065615

Fox, J. A. (2015). Social accountability: What does the evidence really say? *World Development, 72*, 346-361. doi:10.1016/j.worlddev.2015.03.011

Frambes, D., Lehto, R., Sikorskii, A., Tesnjak, I., Given, B., & Wyatt, G. (2017). Fidelity scorecard: evaluation of a caregiver-delivered symptom management intervention. *Journal of Advanced Nursing (John Wiley & Sons, Inc.), 73*(8), 2012-2021. doi:10.1111/jan.13266

Fredriksson, M., Eriksson, M., & Tritter, J. (2017). Who wants to be involved in health care decisions? Comparing preferences for individual and collective involvement in England and Sweden. *BMC Public Health, 18*(1), 18. doi:10.1186/s12889-017-4534-y

Fredriksson, M., Eriksson, M., & Tritter, J. Q. (2018). Involvement that makes an impact on healthcare: Perceptions of the Swedish public. *Scand J Public Health, 46*(4), 471-477. doi:10.1177/1403494817738692

Fredriksson, M., & Tritter, J. Q. (2017). Disentangling patient and public involvement in healthcare decisions: Why the difference matters. *Sociology of health & illness, 39*(1), 95-111. doi:10.1111/1467-9566.12483

Freedman, L. P., & Schaaf, M. (2013). Act global, but think local: accountability at the frontlines. *Reprod Health Matters, 21*(42), 103-112. doi:10.1016/s0968-8080(13)42744-1

Friele, R. D., Reitsma, P. M., & de Jong, J. D. (2015). Complaint handling in healthcare: expectation gaps between physicians and the public; results of a survey study. *BMC Research Notes, 8*(1), 1-7. doi:10.1186/s13104-015-1479-z

Frisancho, A. (2013). Citizen monitoring to promote the right to healthcare and accountability. In *Maternal mortality, human rights and accountability* (pp. 41-58): Routledge.

Frith, L., Young, B., & Woolfall, K. (2014). Patient and public participation in Health Care: Can We Do It Better? *American Journal of Bioethics, 14*(6), 17-18. doi:10.1080/15265161.2014.903642

Frosch, D. L., Moulton, B. W., Wexler, R. M., Holmes-Rovner, M., Volk, R. J., & Levin, C. A. (2011). Shared decision making in the United States: policy and implementation activity on multiple fronts. *Z Evid Fortbild Qual Gesundhwes, 105*(4), 305-312. doi:10.1016/j.zefq.2011.04.004

Gagnon, A. J. (2002). *Responsiveness of the Canadian health care system towards newcomers* (Vol. 40): Commission on the Future of Health Care in Canada.

Gaitonde, R., Muraleedharan, V. R., San Sebastian, M., & Hurtig, A.-K. (2019). Accountability in the health system of Tamil Nadu, India: Exploring its multiple meanings. *Health Research Policy and Systems, 17*(1), 44. doi:10.1186/s12961-019-0448-8

Gaitonde, R., San Sebastian, M., Muraleedharan, V. R., & Hurtig, A. K. (2017). Community action for health in India’s national rural health mission: one policy, many paths. *Soc Sci Med, 188*, 82-90. doi:10.1016/j.socscimed.2017.06.043

Gakidou, E., Murray, C. J. L., & Frenk, J. (2000). *Measuring preferences on health system performance assessment*. Retrieved from Geneva:

Gal, I., & Doron, I. (2007). Informal complaints on health services: Hidden patterns, hidden potentials. *International Journal for Quality in Health Care, 19*(3), 158-163. doi:10.1093/intqhc/mzm006

Gallagher, J. M., & Kupas, D. F. (2012). Experience with an anonymous web-based state EMS safety incident reporting system. *Prehosp Emerg Care, 16*(1), 36-42. doi:10.3109/10903127.2011.626105

Garg, S., & Laskar, A. R. (2010). Community-based monitoring: Key to success of national health programs. *Indian Journal of Community Medicine, 35*(2), 214. Retrieved from <http://www.ijcm.org.in/article.asp?issn=0970-0218;year=2010;volume=35;issue=2;spage=214;epage=216;aulast=Garg>

Garrard, F., & Narayan, H. (2013). Assessing obstetric patient experience: a SERVQUAL questionnaire. *Int J Health Care Qual Assur, 26*(7), 582-592. doi:10.1108/ijhcqa-08-2011-0049

Garza, B. (2015). Increasing the responsiveness of health services in Mexico's Seguro Popular: Three policy proposals for voice and power. *Health Systems & Reform, 1*(3), 235-245. doi:10.1080/23288604.2015.1059538

Gauld, R. (2010). Are elected health boards an effective mechanism for public participation in health service governance? *Health Expectations: An International Journal of Public Participation in Health Care & Health Policy, 13*(4), 369-378. doi:10.1111/j.1369-7625.2010.00605.x

Gauld, R., Al-Wahaibi, S., Chisholm, J., Crabbe, R., Kwon, B., Oh, T., . . . Sohn, S. (2011). Scorecards for health system performance assessment: The New Zealand example. *Health Policy, 103*(2/3), 200-208. Retrieved from <http://ezproxy.uct.ac.za/login?url=https://search.ebscohost.com/login.aspx?direct=true&db=cin20&AN=104595185&site=ehost-live>

Gaunt, A., & Pawlikowska, T. (2018). Physicians actively engaging in seeking feedback for learning through patient outcomes. *Medical Education, 52*(4), 354-356. doi:10.1111/medu.13525

Geldsetzer, P., Haakenstad, A., James, E. K., & Atun, R. (2018). Non-technical health care quality and health system responsiveness in middle-income countries: a cross-sectional study in China, Ghana, India, Mexico, Russia, and South Africa. *Journal Of Global Health, 8*(2), 020417-020417. doi:10.7189/jogh.08.020417

Geletta, S. (2018). Measuring patient satisfaction with medical services using social media generated data. *Int J Health Care Qual Assur, 31*(2), 96-105. doi:10.1108/ijhcqa-12-2016-0183

Genovese, U., Del Sordo, S., Pravettoni, G., Akulin, I. M., Zoja, R., & Casali, M. (2017). A new paradigm on health care accountability to improve the quality of the system: four parameters to achieve individual and collective accountability. *J Glob Health, 7*(1), 010301. doi:10.7189/jogh.07.010301

George, A. (2003). Using accountability to improve reproductive health care. *Reproductive Health Matters 11*(21), 161-170.

George, A. (2009). 'By papers and pens, you can only do so much': views about accountability and human resource management from Indian government health administrators and workers. *Int J Health Plann Manage, 24*(3), 205-224. doi:10.1002/hpm.986

George, A., Subha Sri, B., & Ved, R. (2016). *Strengthening people-centered services through improved accountability: The enabling community maternal health project Gujarat, India*. Retrieved from Gujarat, India:

George, A. S., Erchick, D. J., Zubairu, M. M., Barau, I. Y., & Wonodi, C. (2016). Sparking, supporting and steering change: grounding an accountability framework with viewpoints from Nigerian routine immunization and primary health care government officials. *Health Policy Plan, 31*(9), 1326-1332. doi:10.1093/heapol/czw057

George, A. S., Scott, K., Mehra, V., & Sriram, V. (2016). Synergies, strengths and challenges: findings on community capability from a systematic health systems research literature review. *BMC Health Serv Res, 16*(Suppl 7), 623. doi:10.1186/s12913-016-1860-1

Gigler, B.-S., & Bailur, S. (2014). *Closing the feedback loop: Can technology bridge the accountability gap?* : The World Bank.

Gil, A. P. (2019). Quality procedures and complaints: nursing homes in Portugal. *Journal of Adult Protection, 21*(2), 126-143. doi:10.1108/JAP-09-2018-0018

Giles, S. J., Reynolds, C., Heyhoe, J., & Armitage, G. (2017). Developing a patient-led electronic feedback system for quality and safety within Renal PatientView. *J Ren Care, 43*(1), 37-49. doi:10.1111/jorc.12186

Gill, S. D., Redden-Hoare, J., Dunning, T. L., Hughes, A. J., & Dolley, P. J. (2015). Health services should collect feedback from inpatients at the point of service: Opinions from patients and staff in acute and subacute facilities. *International Journal for Quality in Health Care Advance Access, 27*(6), 507-512. doi:10.1093/intqhc/mzv081

Gillespie, A., & Reader, T. W. (2018). Patient‐centered insights: using health care complaints to reveal hot spots and blind spots in quality and safety. *Milbank Quarterly, 96*(3), 530-567. doi:10.1111/1468-0009.12338

Ginter, E. (2000). World Health Report 2000: the position of Slovak Republic. *Bratislavske Lekarske Listy, 101*(9), 477-483. Retrieved from <http://ezproxy.uct.ac.za/login?url=https://search.ebscohost.com/login.aspx?direct=true&db=awn&AN=11187049&site=ehost-live>

Glattstein-Young, G. S. (2010). *Community health committees as a vehicle for participation in advancing the right to health.* (Masters in Public Health). University of Cape Town, Cape Town.

Golding, L. (2014). *Strengthening community health systems through CHWs and mHealth*. Retrieved from Washington DC:

Gooberman-Hill, R., Horwood, J., & Calnan, M. (2008). Citizens' juries in planning research priorities: Process, engagement and outcome. *Health Expectations: An International Journal of Public Participation in Health Care & Health Policy, 11*(3), 272-281. doi:10.1111/j.1369-7625.2008.00502.x

Goodman, C., Opwora, A., Kabare, M., & Molyneux, S. (2011). Health facility committees and facility management - exploring the nature and depth of their roles in Coast Province, Kenya. *BMC Health Services Research, 11*, 229. doi:10.1186/1472-6963-11-229

Gostin, L., Hodge, J. G., Valentine, N. B., & Nygren-Krug, H. (2003). *The domains of health responsiveness a human rights analysis*. Retrieved from

Grandvoinnet, H., Ghazia, A., & Shomikho, R. (2015). *Opening the black box: The contextual drivers of social accountability* (10.1596/978-1-4648-0482-3). Retrieved from Washington, DC: <https://openknowledge.worldbank.org/bitstream/handle/10986/>

Grant, J., Sears, N. A., & Born, K. (2008). Public engagement and the changing face of health system planning. *Healthc Manage Forum, 21*(4), 22-26. doi:10.1016/s0840-4704(10)60052-x

Green, C., & Soyoola, M. (2008). *Strengthening voice and accountability in the health sector*. Retrieved from

Green, L. W., & Kreuter, M. W. (2002). Fighting back or fighting themselves? Community coalitions against substance abuse and their use of best practices. *Am J Prev Med, 23*(4), 303-306.

Groenewegen, P. P., Kerssens, J. J., Sixma, H. J., van der Eijk, I., & Boerma, W. G. W. (2005). What is important in evaluating health care quality? An international comparison of user views. *BMC Health Services Research, 5*. doi:10.1186/1472-6963-5-16

Gromulska, L., Supranowicz, P., & Wysocki, M. J. (2014). Responsiveness to the hospital patient needs in Poland. *Roczniki Panstwowego Zakladu Higieny, 65*(2), 155-164. Retrieved from <http://ezproxy.uct.ac.za/login?url=https://search.ebscohost.com/login.aspx?direct=true&db=cmedm&AN=25272583&site=ehost-live>

Gulland, J. (2006). Second-tier reviews of complaints in health and social care. *Health & Social Care in the Community, 14*(3), 206-214. doi:10.1111/j.1365-2524.2006.00611.x

Gullo, S., Galavotti, C., & Altman, L. (2016). A review of CARE's Community Score Card experience and evidence. *Un resumen de las experiencias y la evidencia de la Carta de Resultados Comunitarios de CARE., 31*(10), 1467-1478. doi:10.1093/heapol/czw064

Gullo, S., Galavotti, C., Sebert Kuhlmann, A., Msiska, T., Hastings, P., & Marti, C. N. (2017). Effects of a social accountability approach, CARE’s Community Score Card, on reproductive health-related outcomes in Malawi: A cluster-randomized controlled evaluation. *PLoS One, 12*(2), 1-20. doi:10.1371/journal.pone.0171316

Gurung, G., Derrett, S., Gauld, R., & Hill, P. C. (2017). Why service users do not complain or have 'voice': A mixed-methods study from Nepal's rural primary health care system. *BMC Health Services Research, 17*(1), 81. doi:10.1186/s12913-017-2034-5

Gurung, G., Derrett, S., Hill, P. C., & Gauld, R. (2016). Governance challenges in the Nepalese primary health care system: time to focus on greater community engagement? *Int J Health Plann Manage, 31*(2), 167-174. doi:10.1002/hpm.2290

Gurung, G., Derrett, S., Hill, P. C., & Gauld, R. (2018). Nepal's Health Facility Operation and Management Committees: exploring community participation and influence in the Dang district's primary care clinics. *Prim Health Care Res Dev, 19*(5), 492-502. doi:10.1017/s1463423618000026

Gurung, G., Gauld, R., Hill, P. C., & Derrett, S. (2018). Citizen's Charter in a primary health-care setting of Nepal: An accountability tool or a 'mere wall poster'? *Health Expectations: An International Journal of Public Participation in Health Care & Health Policy, 21*(1), 149-158. doi:10.1111/hex.12596

Gurung, G., & Tuladhar, S. (2013). Fostering good governance at peripheral public health facilities: an experience from Nepal. *Rural Remote Health, 13*(2), 2042.

Ha, B. T. T., Mirzoev, T., & Morgan, R. (2015). Patient complaints in healthcare services in Vietnam's health system. *SAGE Open Med, 3*, 2050312115610127. doi:10.1177/2050312115610127

Habibullah, S. (2013). *Responsiveness of the federal health system to the needs of 18-45 year old adults with physical disabilities in Islamabad, Pakistan.* (Doctoral degree). ProQuest Information & Learning, Retrieved from <http://ezproxy.uct.ac.za/login?url=https://search.ebscohost.com/login.aspx?direct=true&db=psyh&AN=2013-99180-017&site=ehost-live> Available from EBSCOhost psyh database.

Hagg, E., Dahinten, V. S., & Currie, L. M. (2018). The emerging use of social media for health-related purposes in low and middle-income countries: A scoping review. *International journal of medical informatics, 115*, 92-105. doi:10.1016/j.ijmedinf.2018.04.010

Hamal, M., de Cock Buning, T., De Brouwere, V., Bardají, A., Dieleman, M., & Bardají, A. (2018). How does social accountability contribute to better maternal health outcomes? A qualitative study on perceived changes with government and civil society actors in Gujarat, India. *BMC Health Services Research, 18*(1), N.PAG-N.PAG. doi:10.1186/s12913-018-3453-7

Hamal, M., Dieleman, M., De Brouwere, V., & de Cock Buning, T. (2018). How do accountability problems lead to maternal health inequities? A review of qualitative literature from Indian public sector. *Public health reviews, 39*(1), 9.

Hamal, M., Heiter, K., Schoenmakers, L., Smid, M., de Cock Buning, T., De Brouwere, V., . . . Dieleman, M. (2019). Social accountability in maternal health services in the far-western development region in Nepal: an exploratory study. *International Journal of Health Policy and Management, 8*(5), 280-291. doi:10.15171/ijhpm.2019.05

Hamid, S. A., & Begum, A. (2019). Responsiveness of the urban primary health care delivery system in Bangladesh: A comparative analysis. *The International Journal of Health Planning and Management, 34*(1), 251-262. doi:10.1002/hpm.2626

Han, E., Hudson Scholle, S., Morton, S., Bechtel, C., & Kessler, R. (2013). Survey shows that fewer than a third of patient-centered medical home practices engage patients in quality improvement. *Health Aff (Millwood), 32*(2), 368-375. doi:10.1377/hlthaff.2012.1183

Haq, Z. U., Sood, S., Yansen, S., Javeed, S., & Ali, N. (2010). Using TV talk show for public health media advocacy: A case study. *Journal of the Pakistan Medical Association, 60*(6), 460-464.

Harter, M., Muller, H., Dirmaier, J., Donner-Banzhoff, N., Bieber, C., & Eich, W. (2011). Patient participation and shared decision making in Germany - history, agents and current transfer to practice. *Z Evid Fortbild Qual Gesundhwes, 105*(4), 263-270. doi:10.1016/j.zefq.2011.04.002

Harvey, J., & Powell, J. (2019). Harnessing mobile devices to support the delivery of community-based clinical care: a participatory evaluation. *BMC Medical Informatics & Decision Making, 19*(1), N.PAG-N.PAG. doi:10.1186/s12911-019-0869-x

Hawkins, C. M., DeLaO, A. J., & Hung, C. (2016). Social media and the patient experience. *Journal of the American College of Radiology, 13*(12), 1615-1621. doi:10.1016/j.jacr.2016.09.006

Hefner, J. L., Hilligoss, B., Sieck, C., Walker, D. M., Sova, L., Song, P. H., & McAlearney, A. S. (2016). Meaningful engagement of ACOS with communities: the new population health management. *Med Care, 54*(11), 970-976. doi:10.1097/mlr.0000000000000622

Hernández, A., Ruano, A. L., Hurtig, A.-K., Goicolea, I., San Sebastián, M., & Flores, W. (2019). Pathways to accountability in rural Guatemala: A qualitative comparative analysis of citizen-led initiatives for the right to health of indigenous populations. *World Development, 113*, 392-401. doi:10.1016/j.worlddev.2018.09.020

Ho, L. S., Labrecque, G., Batonon, I., Salsi, V., & Ratnayake, R. (2015). Effects of a community scorecard on improving the local health system in Eastern Democratic Republic of Congo: qualitative evidence using the most significant change technique. *Conflict & Health, 9*(1), 1-11. doi:10.1186/s13031-015-0055-4

Hogg, C., & Williamson, C. (2001). Whose interests do lay people represent? Towards an understanding of the role of lay people as members of committees. In *Health Expect* (2001/04/05 ed., Vol. 4, pp. 2-9).

Holeman, I., Cookson, T. P., & Pagliari, C. (2016). Digital technology for health sector governance in low and middle income countries: a scoping review. *Journal Of Global Health, 6*(2), 1-11. doi:10.7189/jogh.06.020408

Househ, M., Borycki, E., & Kushniruk, A. (2014). Empowering patients through social media: the benefits and challenges. *Health Informatics J, 20*(1), 50-58. doi:10.1177/1460458213476969

Hovey, R. B., Morck, A., Nettleton, S., Robin, S., Bullis, D., Findlay, A., & Massfeller, H. (2010). Partners in our care: patient safety from a patient perspective. *Qual Saf Health Care, 19*(6), e59. doi:10.1136/qshc.2008.030908

Howard‐Grabman, L. (2000). Bridging the gap between communities and service providers: developing accountability through community mobilisation approaches. *IDS Bulletin, 31*(1), 88-96.

Hsu, C.-C., Chen, L., Hu, Y.-W., Yip, W., & Shu, C.-C. (2006). The dimensions of responsiveness of a health system: A Taiwanese perspective. *BMC Public Health, 6*(1), 1-7. doi:10.1186/1471-2458-6-72

Human Rights Watch. (2011). *‘Stop making excuses’ accountability for maternal health care in South Africa*. Retrieved from New York:

Hussein, J., & Okonofua, F. (2012). Time for Action: Audit, accountability and confidential enquiries into maternal deaths in Nigeria. *African Journal of Reproductive Health, 16*(1).

Hussin, A. H., Ali, F. M., Reka, H., & Gjebrea, O. (2015). Tracking access, utilization and health system responsiveness to inform evidence-based health care policy: the case of Qatar. *Journal of Local and Global Health Perspectives, 2015*(1), 2.

Ibe, O., Honda, A., Etiaba, E., Ezumah, N., Hanson, K., & Onwujekwe, O. (2017). Do beneficiaries' views matter in healthcare purchasing decisions? Experiences from the Nigerian tax-funded health system and the formal sector social health insurance program of the National Health Insurance Scheme. *International Journal for Equity in Health, 16*, 1-11. doi:10.1186/s12939-017-0711-y

Jackman, M. (2010). Charter review as a health care accountability mechanism in Canada. *Health LJ, 18*, 1.

James, T. L., Villacis Calderon, E. D., & Cook, D. F. (2017). Exploring patient perceptions of healthcare service quality through analysis of unstructured feedback. *Expert Systems with Applications, 71*, 479-492. doi:10.1016/j.eswa.2016.11.004

Janse van Rensburg, A., Petersen, I., Wouters, E., Engelbrecht, M., Kigozi, G., Fourie, P., . . . Bracke, P. (2018). State and non-state mental health service collaboration in a South African district: A mixed methods study. *Health Policy and Planning*, czy017-czy017. doi:10.1093/heapol/czy017

Jing, L., Assanangkornchai, S., Lin, L., Le, C., Jing, Y., McNeil, E. B., . . . McNeil, E. B. (2016). Can socio-economic differences explain low expectation of health services among HIV patients compared to non-HIV counterparts? *BMC Public Health, 16*(1), 1-9. doi:10.1186/s12889-016-3609-5

Joarder, T. (2015). *Understanding and measuring responsiveness of human resources for health in rural Bangladesh.* Johns Hopkins University,

Joarder, T., George, A., Ahmed, S. M., Rashid, S. F., & Sarker, M. (2017). What constitutes responsiveness of physicians: A qualitative study in rural Bangladesh. *PLoS One, 12*(12), 1-19. doi:10.1371/journal.pone.0189962

Joarder, T., George, A., Sarker, M., Ahmed, S., & Peters, D. H. (2017). Who are more responsive? Mixed-methods comparison of public and private sector physicians in rural Bangladesh. *Health Policy and Planning, 32*(suppl_3), iii14-iii24. doi:10.1093/heapol/czx111

Joarder, T., Mahmud, I., Sarker, M., George, A., & Rao, K. D. (2017). Development and validation of a structured observation scale to measure responsiveness of physicians in rural Bangladesh. *BMC Health Services Research, 17*, 1-12. doi:10.1186/s12913-017-2722-1

Johnston, S., Abelson, J., Wong, S. T., Langton, J., Hogel, M., Burge, F., & Hogg, W. (2019). Citizen perspectives on the use of publicly reported primary care performance information: Results from citizen‐patient dialogues in three Canadian provinces. *Health Expectations: An International Journal of Public Participation in Health Care & Health Policy*. doi:10.1111/hex.12902

Jolles, M. P., & Wells, R. (2017). Does caregiver participation in decision making within child welfare agencies influence children's primary and mental health care service use? *Child Care Health Dev, 43*(2), 192-201. doi:10.1111/cch.12384

Jones, A. M., Rice, N., Robone, S., & Dias, P. R. (2011). Inequality and polarisation in health systems’ responsiveness: A cross-country analysis. *Journal of Health Economics, 30*(4), 616-625. doi:10.1016/j.jhealeco.2011.05.003

Kalter, H. D., Mohan, P., Mishra, A., Gaonkar, N., Biswas, A. B., Balakrishnan, S., . . . Babille, M. (2011). Maternal death inquiry and response in India--the impact of contextual factors on defining an optimal model to help meet critical maternal health policy objectives. *Health Res Policy Syst, 9*, 41. doi:10.1186/1478-4505-9-41

Kamal, A. H., Kirkland, K. B., Meier, D. E., Morgan, T. S., Nelson, E. C., & Pantilat, S. Z. (2018). A Person-Centered, Registry-Based Learning Health System for Palliative Care: A Path to Coproducing Better Outcomes, Experience, Value, and Science. *Journal of Palliative Medicine, 21*, S-61-S-67. doi:10.1089/jpm.2017.0354

Kanthor, J., Seligman, B., Dereje, T., & Tarantino, L. (2014). *Engaging civil society in health finance and governance: A guide for practitioners*. Retrieved from Bethesda, MD:

Kapiriri, L., Norheim, O. F., & Heggenhougen, K. (2003). Public participation in health planning and priority setting at the district level in Uganda. *Health Policy Plan, 18*(2), 205-213. doi:10.1093/heapol/czg025

Karami-Tanha, F., Moradi-Lakeh, M., Fallah-Abadi, H., & Nojomi, M. (2014). Health system responsiveness for care of patients with heart failure: Evidence form a university hospital. *Archives of Iranian Medicine (AIM), 17*(11), 736-740. doi:0141711/AIM.003

Karuga, R. N., Kok, M., Mbindyo, P., Hilverda, F., Otiso, L., Kavoo, D., . . . Dieleman, M. (2019). “It’s like these CHCs don’t exist, are they featured anywhere?”: Social network analysis of community health committees in a rural and urban setting in Kenya. *PLoS One, 14*(8), 1-19. doi:10.1371/journal.pone.0220836

Kashkoli, S. A., Zarei, E., Daneshkohan, A., & Khodakarim, S. (2017). Hospital responsiveness and its effect on overall patient satisfaction. *International Journal Of Health Care Quality Assurance*.

Katahoire, A. R., Henriksson, D. K., Ssegujja, E., Waiswa, P., Ayebare, F., Bagenda, D., . . . Peterson, S. S. (2015). Improving child survival through a district management strengthening and community empowerment intervention: early implementation experiences from Uganda. *BMC Public Health, 15*, 797. doi:10.1186/s12889-015-2129-z

Kaufman, J., Liu, Y., & Fang, J. (2012). Improving reproductive health in rural China through participatory planning. *Glob Public Health, 7*(8), 856-868. doi:10.1080/17441692.2012.674958

Kennedy, K. M., Green, P. G., & Payne-James, J. J. (2017). Complaints against health-care professionals providing police custodial and forensic medical/health-care services and sexual offence examiner services in England, Wales and Northern Ireland. *Medicine, Science & the Law, 57*(1), 12-32. doi:10.1177/0025802417691391

Kerrison, S., & Pollock, A. (2001). Complaints as accountability? the case of health care in the United Kingdom. In *Public Law* (pp. 115-133).

Kerssens, J. J., Groenewegen, P. P., Sixma, H. J., Boerma, W. G. W., & van der Eijk, I. (2004). Comparison of patient evaluations of health care quality in relation to WHO measures of achievement in 12 European countries. *Bulletin of the World Health Organization, 82*(2), 106-114. Retrieved from <http://ezproxy.uct.ac.za/login?url=https://search.ebscohost.com/login.aspx?direct=true&db=aph&AN=12727213&site=ehost-live>

<https://www.ncbi.nlm.nih.gov/pmc/articles/PMC2585905/pdf/15042232.pdf>

Khanna, R. (2013). *Ethical issues in community based monitoring of health programmes: Reflections from India*. Retrieved from SAHAJ, India:

Khunte, P., & Walimbe, A. (2011). *People are reclaiming the public health system*. Retrieved from India:

Kilewo, E. G., & Frumence, G. (2015). Factors that hinder community participation in developing and implementing comprehensive council health plans in Manyoni District, Tanzania. *Glob Health Action, 8*, 26461. doi:10.3402/gha.v8.26461

King, G., Heaney, D. J., Boddy, D., O'Donnell, C. A., Clark, J. S., & Mair, F. S. (2011). Exploring public perspectives on e‐health: Findings from two citizen juries. *Health Expectations: An International Journal of Public Participation in Health Care & Health Policy, 14*(4), 351-360. doi:10.1111/j.1369-7625.2010.00637.x

Kite, J., Foley, B. C., Grunseit, A. C., & Freeman, B. (2016). Please like me: Facebook and public health

communication. *PLoS One, 11*(9), e0162765. doi:10.1371/journal.pone.0162765

Kolasa, K., Dohnalik, J., Borek, E., Siemiątkowski, M., & Ścibiorski, C. (2014). The paradox of public participation in the healthcare in Poland—What citizens want, and what they think. *Health Policy, 118*(2), 159-165. doi:10.1016/j.healthpol.2014.09.015

Kowal, P., Naidoo, N., Williams, S. R., & Chatterji, S. (2011). Performance of the health system in China and Asia as measured by responsiveness. *Health, 3*(10), 638-646.

Kramer, S., Solomon, R., & Dingman, C. (2009). Achieving accountability. *Healthcare Quarterly, 12*, 22-27. Retrieved from <http://ezproxy.uct.ac.za/login?url=https://search.ebscohost.com/login.aspx?direct=true&db=cin20&AN=105360725&site=ehost-live>

Kroneman, M., van Erp, K., & Groenewegen, P. (2019). Community participation in primary care: willingness to participate, a web survey in the Netherlands. *Prim Health Care Res Dev, 20*, e13. doi:10.1017/s1463423618000695

Kypri, K., & Maclennan, B. (2014). Public participation in local alcohol regulation: Findings from a survey of New Zealand communities. *Drug Alcohol Rev, 33*(1), 59-63. doi:10.1111/dar.12094

Lambert, B. L., Centomani, N. M., Smith, K. M., Helmchen, L. A., Bhaumik, D. K., Jalundhwala, Y. J., & McDonald, T. B. (2016). The "seven pillars" response to patient safety incidents: effects on medical liability processes and outcomes. *Health Services Research, 51*, 2491-2515. doi:10.1111/1475-6773.12548

Lapsley, P. M. (2004). Public involvement in health care: public involvement is needed at highest level. *BMJ, 328*(7437), 462. doi:10.1136/bmj.328.7437.462-a

Lawton, R., O'Hara, J. K., Sheard, L., Armitage, G., Cocks, K., Buckley, H., . . . Wright, J. (2017). Can patient involvement improve patient safety? A cluster randomised control trial of the Patient Reporting and Action for a Safe Environment (PRASE) intervention. *BMJ Qual Saf, 26*(8), 622-631. doi:10.1136/bmjqs-2016-005570

Lechat, L., Bonnet, E., Queuille, L., Traoré, Z., Somé, P.-A., & Ridde, V. (2019). Relevance of a toll-free call service using an interactive voice server to strengthen health system governance and responsiveness in Burkina Faso. *International Journal of Health Policy & Management, 8*(6), 353-364. doi:10.15171/ijhpm.2019.13

Lecoanet, A., Sellier, E., Carpentier, F., Maignan, M., Seigneurin, A., & Francois, P. (2014). Experience feedback committee in emergency medicine: a tool for security management. *Emerg Med J, 31*(11), 894-898. doi:10.1136/emermed-2013-202767

Lee, D. (2012). Mental health policy in California: The 'millionaire's tax' and the Mental Health Services Oversight and Accountability Commission. *Mental Health Review Journal, 17*(4), 211-220. doi:10.1108/13619321211289281

Legare, F., Boivin, A., van der Weijden, T., Pakenham, C., Burgers, J., Legare, J., . . . Gagnon, S. (2011). Patient and public involvement in clinical practice guidelines: a knowledge synthesis of existing programs. *Med Decis Making, 31*(6), E45-74. doi:10.1177/0272989x11424401

Leibert, M. (2010). Performance of state medical boards: implications for hospitals and health systems. *Hospital Topics, 88*(4), 107-115. doi:10.1080/00185868.2010.528260

Leskela, J., Viitanen, E., & Piirainen, A. (2005). Client feedback on physiotherapy counselling in primary health care. *Patient Educ Couns, 56*(2), 218-224. doi:10.1016/j.pec.2004.02.015

Li, J., Chongsuvivatwong, V., Assanangkornchai, S., McNeil, E. B., & Cai, L. (2018). Comparison of health system responsiveness between HIV and non-HIV patients at infectious disease clinics in Yunnan, China. *Patient Preference And Adherence, 12*, 1129-1137. doi:10.2147/PPA.S163416

Liabsuetrakul, T., Petmanee, P., Sanguanchua, S., & Oumudee, N. (2012). Health system responsiveness for delivery care in Southern Thailand. *International Journal for Quality in Health Care, 24*(2), 169-175. doi:10.1093/intqhc/mzr085

Lin, S. Y., Yang, H. C., Chiang, H. Y., & Lee, S. L. (2019). A cross‐validation study of the incident‐reporting attitude scale for staff in long‐term care facilities—A cross‐sectional study. *Journal of Clinical Nursing (John Wiley & Sons, Inc.), 28*(15/16), 2858-2867. doi:10.1111/jocn.14869

Lindsay, P., Sandall, J., & Humphrey, C. (2012). The social dimensions of safety incident reporting in maternity care: the influence of working relationships and group processes. *Social Science and Med, 75*(10), 1793-1799. doi:10.1016/j.socscimed.2012.06.030

Listening Project. (2011). *Feedback mechanisms in international assistance organizations*. Retrieved from Cambridge, MA:

Litva, A., Coast, J., Donovan, J., Eyles, J., Shepherd, M., Tacchi, J., . . . Morgan, K. (2002). ‘The public is too subjective’: Public involvement at different levels of health-care decision making. *Social Science & Medicine, 54*(12), 1825-1837. doi:10.1016/s0277-9536(01)00151-4

Lodenstein, E., Dieleman, M., Gerretsen, B., & Broerse, J. E. W. (2016). Health provider responsiveness to social accountability initiatives in low- and middle-income countries: A realist review. *Health Policy and Planning, 32*(1), 125-140. doi:10.1093/heapol/czw089

Lodenstein, E., Ingemann, C., Molenaar, J. M., Dieleman, M., & Broerse, J. E. W. (2018). Informal social accountability in maternal health service delivery: A study in Northern Malawi. *PLoS One, 13*(4), 1-17. doi:10.1371/journal.pone.0195671

Lodenstein, E., Mafuta, E., Kpatchavi, A. C., Servais, J., Dieleman, M., Broerse, J. E. W., . . . Barry, A. A. B. (2017). Social accountability in primary health care in West and Central Africa: Exploring the role of health facility committees. *BMC Health Services Research, 17*, 1-15. doi:10.1186/s12913-017-2344-7

Lodenstein, E., Molenaar, J. M., Ingemann, C., Botha, K., Mkandawire, J. J., Liem, L., . . . Dieleman, M. (2019). “We come as friends”: approaches to social accountability by health committees in Northern Malawi. *BMC Health Services Research, 19*(1), 279. doi:10.1186/s12913-019-4069-2

Lucas, G., Gallagher, A., Zasada, M., Austin, Z., Jago, R., Banks, S., & van der Gaag, A. (2019). Understanding complaints about paramedics: a qualitative exploration in a UK context. *Australasian Journal of Paramedicine, 16*, 1-7. Retrieved from <http://ezproxy.uct.ac.za/login?url=https://search.ebscohost.com/login.aspx?direct=true&db=cin20&AN=135512601&site=ehost-live>

Lucock, M., Halstead, J., Leach, C., Barkham, M., Tucker, S., Randal, C., . . . Saxon, D. (2015). A mixed-method investigation of patient monitoring and enhanced feedback in routine practice: Barriers and facilitators. *Psychother Res, 25*(6), 633-646. doi:10.1080/10503307.2015.1051163

Lunevicius, R., & Rahman, M. H. (2012). Assessment of Lithuanian trauma care service using a conceptual framework for assessing the performance of health system. *European Journal Of Public Health, 22*(1), 26-31. doi:10.1093/eurpub/ckq184

Luo, Q., Wang, Q., Lu, Z., & Liu, J. (2013). Evaluation of responsiveness of community health services in urban China: A quantitative study in Wuhan City. *PLoS One, 8*(5), e62923. doi:10.1371/journal.pone.0062923

MacDonald, D., Barnes, M., Crawford, M., Omeni, E., Wilson, A., & Rose, D. (2015). Service user governors in mental health foundation trusts: accountability or business as usual? *Health Expectations, 18*(6), 2892-2902.

Maddalena, V. (2006). *Governance, public participation and accountability: to whom are regional health authorities accountable?* Paper presented at the Healthcare Management Forum.

Madon, S., & Krishna, S. (2017). Challenges of accountability in resource-poor contexts: lessons about invited spaces from Karnataka’s village health committees. *Oxford Development Studies, 45*(4), 522-541.

Mafuta, E. M., Dieleman, M. A., Essink, L., Khomba, P. N., Zioko, F. M., Mambu, T. N. M., . . . de Cock Buning, T. (2017). Participatory approach to design social accountability interventions to improve maternal health services: A case study from the Democratic Republic of the Congo. *Global Health Research And Policy, 2*, 4-4. doi:10.1186/s41256-017-0024-0

Mafuta, E. M., Dieleman, M. A., Hogema, L. M., Khomba, P. N., Zioko, F. M., Kayembe, P. K., . . . Mambu, T. N. M. (2015). Social accountability for maternal health services in Muanda and Bolenge Health Zones, Democratic Republic of Congo: A situation analysis. *BMC Health Services Research, 15*, 1-17. doi:10.1186/s12913-015-1176-6

Mafuta, E. M., Hogema, L., Mambu, T. N. M., Kiyimbi, P. B., Indebe, B. P., Kayembe, P. K., . . . Dieleman, M. A. (2016). Understanding the local context and its possible influences on shaping, implementing and running social accountability initiatives for maternal health services in rural Democratic Republic of the Congo: a contextual factor analysis. *BMC Health Services Research, 16*, 1-13. doi:10.1186/s12913-016-1895-3

Magruder, K. J., Fields, N. L., & Xu, L. (2019). Abuse, neglect and exploitation in assisted living: an examination of long-term care ombudsman complaint data. *Journal of Elder Abuse & Neglect, 31*(3), 209-224. doi:10.1080/08946566.2019.1590275

Malfait, S., Van Hecke, A., De Bodt, G., Palsterman, N., & Eeckloo, K. (2018). Patient and public involvement in hospital policy-making: Identifying key elements for effective participation. *Health Policy, 122*(4), 380-388. doi:10.1016/j.healthpol.2018.02.007

Malhotra, C., & Do, Y. K. (2013). Socio-economic disparities in health system responsiveness in India. *Health Policy and Planning*. doi:10.1093/heapol/czs051

Malhotra, C., & Do, Y. K. (2017). Public health expenditure and health system responsiveness for low-income individuals: Results from 63 countries. *Health Policy and Planning, 32*(3), 314-319. doi:10.1093/heapol/czw127

Marston, C., Hinton, R., Kean, S., Baral, S., Ahuja, A., Costello, A., & Portela, A. (2016). Community participation for transformative action on women's, children's and adolescents' health. *مشاركة المجتمع للقيام بإجراءات تحويلية بشأن صحة النساء والأطفال والمراهقين., 94*(5), 376-382. doi:10.2471/BLT.15.168492

Martinez, M. G., & Kohler, J. C. (2016). Civil society participation in the health system: The case of Brazil's Health Councils. *Global Health, 12*(1), 64. doi:10.1186/s12992-016-0197-1

Mathews, M., Ryan, D., & Bulman, D. (2015). What does satisfaction with wait times mean to cancer patients? *BMC Cancer, 15*, 1-7. doi:10.1186/s12885-015-2041-z

McCoy, D. C., Hall, J. A., & Ridge, M. (2011). A systematic review of the literature for evidence on health facility committees in low-and middle-income countries. *Health Policy and Planning, 27*(6), 449-466. doi:10.1093/heapol/czr077

McCreaddie, M., Benwell, B., & Gritti, A. (2018). Traumatic journeys; understanding the rhetoric of patients' complaints. *BMC Health Services Research, 18*(1), N.PAG-N.PAG. doi:10.1186/s12913-018-3339-8

McEvoy, R., & MacFarlane, A. (2013). Community participation in primary care in Ireland: The need for implementation research. *Primary Health Care Research and Development, 14*(2), 126-139. doi:10.1017/S1463423612000163

McNamara, P. (2006). Provider-specific report cards: A tool for health sector accountability in developing countries. *Health Policy and Planning, 21*(2), 101-109. doi:10.1093/heapol/czj009

McNatt, Z., Thompson, J. W., Mengistu, A., Tatek, D., Linnander, E., Ageze, L., . . . Bradley, E. H. (2014). Implementation of hospital governing boards: views from the field. *BMC Health Services Research, 14*(1), 1-20. doi:10.1186/1472-6963-14-178

McNeil, M., & Mumvuma, T. (2006). *Demanding good governance: A stocktaking of social accountability initiatives by civil society in anglophone Africa*. Retrieved from Washington, DC: <http://documents.worldbank.org/curated/en/16430146800>

Megbelayin, E. O., Babalola, Y. O., Kurawa, M. S., Opubiri, I., & Okonkwo, S. N. (2014). How satisfied are patients attending a Nigerian eye clinic in University of Calabar Teaching Hospital. *International Archives of Integrated Medicine, 1*(4), 1-9. Retrieved from <http://ezproxy.uct.ac.za/login?url=https://search.ebscohost.com/login.aspx?direct=true&db=aph&AN=100218693&site=ehost-live>

Mehata, S., Paudel, Y. R., Dariang, M., Aryal, K. K., Paudel, S., Mehta, R., . . . Barnett, S. (2017). Factors determining satisfaction among facility-based maternity clients in Nepal. *BMC Pregnancy Childbirth, 17*(1), 319. doi:10.1186/s12884-017-1532-0

Mello, M. M., Studdert, D. M., Kachalia, A. B., & Brennan, T. A. (2006). 'Health Courts' and accountability for patient safety. *Milbank Quarterly, 84*(3), 459-492. doi:10.1111/j.1468-0009.2006.00455.x

Melo, D. d. S., Martins, R. D., Jesus, R. P. F. S. d., Samico, I. C., & Santo, A. C. G. d. E. (2017). Assessment of the responsiveness of a public health service from the perspective of older adults. *Revista De Saude Publica, 51*, 62-62. Retrieved from <http://ezproxy.uct.ac.za/login?url=https://search.ebscohost.com/login.aspx?direct=true&db=awn&AN=28678911&site=ehost-live>

Miatello, A., Mulvale, G., Hackett, C., Mulvale, A., Kutty, A., & Alshazly, F. (2018). Data Elicited Through Apps for Health Systems Improvement: Lessons From Using the myEXP Suite of Smartphone and Web Apps. *International Journal of Qualitative Methods, 17*(1), 1-13. doi:10.1177/1609406918798433

Miller, F. A., Patton, S. J., Dobrow, M., Marshall, D. A., & Berta, W. (2018). Public involvement and health research system governance: a qualitative study. *Health Res Policy Syst, 16*(1), 87. doi:10.1186/s12961-018-0361-6

Miller, J. S., Mhalu, A., Chalamilla, G., Siril, H., Kaaya, S., Tito, J., . . . Hirschhorn, L. R. (2014). Patient satisfaction with HIV/AIDS care at private clinics in Dar es Salaam, Tanzania. *AIDS Care, 26*(9), 1150-1154. doi:10.1080/09540121.2014.882487

Mills, A. (2017). Resilient and responsive health systems in a changing world. *Health Policy and Planning, 32*(suppl_3), iii1-iii2. doi:10.1093/heapol/czx117

Mirzoev, T., & Kane, S. (2018). Key strategies to improve systems for managing patient complaints within health facilities - what can we learn from the existing literature? *Glob Health Action, 11*(1), 1458938-1458938. doi:10.1080/16549716.2018.1458938

Mishima, S. M., Campos, A. C., Matumoto, S., & Fortuna, C. M. (2016). Client satisfaction from the perspective of responsiveness: strategy for analysis of universal systems? *Rev Lat Am Enfermagem, 24*, e2674. doi:10.1590/1518-8345.1089.2674

Mitton, C., Smith, N., Peacock, S., Evoy, B., & Abelson, J. (2009). Public participation in health care priority setting: A scoping review. *Health Policy, 91*(3), 219-228. doi:10.1016/j.healthpol.2009.01.005

Mockford, C., Staniszewska, S., Griffiths, F., & Herron-Marx, S. (2012). The impact of patient and public involvement on UK NHS health care: a systematic review. *Int J Qual Health Care, 24*(1), 28-38. doi:10.1093/intqhc/mzr066

Moeller, A. D., Rasmussen, K., & Nielsen, K. J. (2016). Learning and feedback from the Danish patient safety incident reporting system can be improved. *Danish Medical Journal, 63*(6).

Mohammed, S., Bermejo, J. L., Souares, A., Sauerborn, R., & Dong, H. (2013). Assessing responsiveness of health care services within a health insurance scheme in Nigeria: Users’ perspectives. *BMC Health Services Research, 13*(1), 502. doi:10.1186/1472-6963-13-502

Molyneux, S., Atela, M., Angwenyi, I., & Goodman, C. (2012). Community accountability at peripheral health facilities: A review of the empirical literature and development of a conceptual framework. *Health Policy and Planning*, 1–14. doi:10.1093/heapol/czr083

Molyneux, S., Sariola, S., Allman, D., Dijkstra, M., Gichuru, E., Graham, S., . . . Sanders, E. (2016). Public/community engagement in health research with men who have sex with men in sub-Saharan Africa: challenges and opportunities. *Health Res Policy Syst, 14*(1), 40. doi:10.1186/s12961-016-0106-3

Morone, J. A., & Kilbreth, E. H. (2003). Power to the people? Restoring citizen participation. *J Health Polit Policy Law, 28*(2-3), 271-288. Retrieved from <https://watermark.silverchair.com/JHPPL28.2-3-05Morone.pdf?token=AQECAHi208BE49Ooan9kkhW_Ercy7Dm3ZL_9Cf3qfKAc485ysgAAAlEwggJNBgkqhkiG9w0BBwagggI-MIICOgIBADCCAjMGCSqGSIb3DQEHATAeBglghkgBZQMEAS4wEQQMvvq98Gr-llS-dwwAAgEQgIICBML9YnegKfPd86KpdQzx4gAGAjuw2DkjruFtUk5X5jEjvGfWYQ1QiF265ZGiL4ztPJ67kDf2DexNvP1sUl0fTCV6Y9h6lNbhZDYTtR4dHNLIMLePoZuYAiRIGwZdPaSxKn5GgfyLJRINyw50bYBqOrNbHnaR_fdIqHTZwMGTB_dT-VNN43poeRh2JO8e3H1mbnzgMXbFvl2qmc-R17wfdrw3SFf3-UL9HQI1Ur-2Shmc8sCJvqJ6C1UUaVU2ynm2lNBB44Tpd4Z0SlI7giS0BVqn7q0ugiXmy_zLQdPQ42cnbtA8IF8ljcqsaXsNng7-JhoG_nq0toUI4luWYmw9xE8d6kzqsgpEi1uIC6WweeJv2QJRiHomk7b9O3ZyOsBSsF6MrF0CcwJ_Dfxzk5eiUMlFBepX8_nNcbzrFH2dyuKeRsS0vj_EZSjfMjMbDAgY2WEotbfI7ujI-ElALMolQuGhQrxfb48HUvsIHSmVfhjybgo_iO4p1jf7Dj37DBT1XP3f-toB89Dfh5wBAlVpNgeETxpEYB2W6Fm7IQ1w4tumIrXnmP0xXjELjXO3TMwuUHr69YVjBW60ccfQuzEMMRSbPl9ep_kKFFl8WVmc554DGFe33EwG2YHXqWKgb1D5J6JaR28neHhF_xF3oaS_xCGLJSwg9k-Dn4Dy-XEIx5u_uzLQ6g>

Morrison, C., & Dearden, A. (2013). Beyond tokenistic participation: using representational artefacts to enable meaningful public participation in health service design. *Health Policy, 112*(3), 179-186. doi:10.1016/j.healthpol.2013.05.008

Mosconi, P., Colombo, C., Satolli, R., & Liberati, A. (2007). PartecipaSalute, an Italian project to involve lay people, patients' associations and scientific-medical representatives in the health debate. *Health Expect, 10*(2), 194-204. doi:10.1111/j.1369-7625.2007.00444.x

Mubyazi, G. M., Mushi, A., Kamugisha, M., Massaga, J., Mdira, K. Y., Segeja, M., & Njunwa, K. J. (2007). Community views on health sector reform and their participation in health priority setting: Case of Lushoto and Muheza districts, Tanzania. *Journal of Public Health, 29*(2), 147-156. doi:10.1093/pubmed/fdm016

Mubyazi, G. M., Mushi, A. K., Shayo, E., Mdira, K., Ikingura, J., Mutagwaba, D., . . . Njunwa, K. J. (2007). Local primary health care committees and community-based health workers in Mkuranga district, Tanzania. *Studies on Ethno-Medicine, 1*(1), 27-35. doi:10.1080/09735070.2007.11886298

Mukinda, F. K., Van Belle, S., George, A., & Schneider, H. (2019). The crowded space of local accountability for maternal, newborn and child health: a case study of the South African health system. *Health Policy and Planning*. doi:10.1093/heapol/czz162

Mullen, C., Hughes, D., & Vincent-Jones, P. (2011). The democratic potential of public participation: healthcare governance in England. *Social & Legal Studies, 20*(1), 21-38. doi:10.1177/0964663910391349

Mulumba, M., London, L., Nantaba, J., & Ngwena, C. (2018). Using health committees to promote community participation as a social determinant of the right to health: lessons from Uganda and South Africa. *Health & Human Rights: An International Journal, 20*(2), 11-17. Retrieved from <http://ezproxy.uct.ac.za/login?url=https://search.ebscohost.com/login.aspx?direct=true&db=sih&AN=133554029&site=ehost-live>

Mulvale, G., Chodos, H., Bartram, M., MacKinnon, M. P., & Abud, M. (2014). Engaging civil society through deliberative dialogue to create the first Mental Health Strategy for Canada: Changing Directions, Changing Lives. *Soc Sci Med, 123*, 262-268. doi:10.1016/j.socscimed.2014.07.029

Munro, N., & Duckett, J. (2016). Explaining public satisfaction with health‐care systems: Findings from a nationwide survey in China. *Health Expectations: An International Journal of Public Participation in Health Care & Health Policy, 19*(3), 654-666. doi:10.1111/hex.12429

Murante, A. M., Seghieri, C., Vainieri, M., & Schafer, W. L. A. (2017). Patient-perceived responsiveness of primary care systems across Europe and the relationship with the health expenditure and remuneration systems of primary care doctors. *Soc Sci Med, 186*, 139-147. doi:10.1016/j.socscimed.2017.06.005

Murante, A. M., Vainieri, M., Rojas, D., & Nuti, S. (2014). Does feedback influence patient - professional communication? Empirical evidence from Italy. *Health Policy, 116*(2-3), 273-280. doi:10.1016/j.healthpol.2014.02.001

Murray, C., & Frenk, J. (2001). World Health Report 2000: a step towards evidence-based health policy. *Lancet, 357*(9269), 1698. doi:10.1016/S0140-6736(00)04826-1

Murray, C. J. L., & Evans, D. B. (Eds.). (2003). *Health systems performance assessment: debates, methods and empiricism*. Geneva: World Health Organisation.

Murray, C. J. L., & Frenk, J. (1999). A WHO framework for health system performance assessment. In *GPE Discussion Paper Series: No.6*. Geneva: World Health Organization.

Murray, C. J. L., & Frenk, J. (2000). A framework for assessing the performance of health systems. *Bulletin of the World Health Organization, 78*(6), 717-731. Retrieved from <https://www.ncbi.nlm.nih.gov/pubmed/10916909>

<https://www.ncbi.nlm.nih.gov/pmc/articles/PMC2560787/pdf/10916909.pdf>

Murthy, R. K., & Klugman, B. (2004). Service accountability and community participation in the context of health sector reforms in Asia: Implications for sexual and reproductive health services. *Health Policy and Planning, 19*(suppl 1), i78-i86. doi:10.1093/heapol/czh048

Musgrove, P. (2003). Judging health systems: reflections on WHO's methods. *Lancet, 361*, 1817-1820. Retrieved from <http://ezproxy.uct.ac.za/login?url=https://search.ebscohost.com/login.aspx?direct=true&db=gsa&AN=508851803&site=ehost-live>

NÂDÂȘAn, V., & ÁBrÁM, Z. (2016). How credible are the Romanian health-related websites? A cross-sectional study. *Acta Medica Transilvanica, 21*(3), 1-4. Retrieved from <http://ezproxy.uct.ac.za/login?url=https://search.ebscohost.com/login.aspx?direct=true&db=aph&AN=118857200&site=ehost-live>

Nagy, M., Chiarella, M., Bennett, B., Walton, M., & Carney, T. (2018). Health care complaint journeys for system comparison. *International Journal of Health Care Quality Assurance (09526862), 31*(8), 878-887. doi:10.1108/IJHCQA-01-2017-0002

Najafi, F., Karami-Matin, B., Rezaei, S., Rajabi-Gilan, N., & Soofi, M. (2016). Health system responsiveness after health sector evolution plan (HSEP): An inpatient survey in Kermanshah in 2015. *Medical Journal Of The Islamic Republic Of Iran, 30*, 387-387. Retrieved from <http://ezproxy.uct.ac.za/login?url=https://search.ebscohost.com/login.aspx?direct=true&db=cmedm&AN=27493931&site=ehost-live>

Nakajima, K., Kurata, Y., & Takeda, H. (2005). A web-based incident reporting system and multidisciplinary collaborative projects for patient safety in a Japanese hospital. *Qual Saf Health Care, 14*(2), 123-129. doi:10.1136/qshc.2003.008607

Nambisan, P., Gustafson, D. H., Hawkins, R., & Pingree, S. (2016). Social support and responsiveness in online patient communities: impact on service quality perceptions. *Health Expectations, 19*(1), 87-97. doi:10.1111/hex.12332

Narayanan, A., & Greco, M. (2014). The Dental Practice Questionnaire: a patient feedback tool for improving the quality of dental practices. *Aust Dent J, 59*(3), 334-348. doi:10.1111/adj.12200

Nathan, S., Johnston, L., & Braithwaite, J. (2011). The role of community representatives on health service committees: Staff expectations vs. reality. *Health Expectations, 14*(3), 272-284. doi:10.1111/j.1369-7625.2010.00628.x

Navarro, V. (2001). The new conventional wisdom: An evaluation of the WHO report health systems: Improving performance. *International Journal of Health Services, 31*(1), 23-33.

Njeru, M. K., Blystad, A., Nyamongo, I. K., & Fylkesnes, K. (2009). A critical assessment of the WHO responsiveness tool: Lessons from voluntary HIV testing and counselling services in Kenya. *BMC Health Services Research, 9*(1), 1. doi:10.1186/1472-6963-9-243

Nowotny, B. M., Loh, E., Lorenz, K., & Wallace, E. M. (2019). Sharing the pain: lessons from missed opportunities for healthcare improvement from patient complaints and litigation in the Australian health system. *Australian Health Review, 43*(4), 382-391. doi:10.1071/AH17266

Nswilla, A., Kapologwe, N. A., Kibusi, S. M., Kalolo, A., Chaula, Z., Teuscher, T., . . . Borghi, J. (2019). Understanding the implementation of Direct Health Facility Financing and its effect on health system performance in Tanzania: a non-controlled before and after mixed method study protocol. *Health Research Policy & Systems, 17*(1), N.PAG-N.PAG. doi:10.1186/s12961-018-0400-3

Nunes, R., Brandao, C., & Rego, G. (2011). Public accountability and sunshine healthcare regulation. *Health Care Anal, 19*(4), 352-364. doi:10.1007/s10728-010-0156-6

Nunes, R., Rego, G., & Brandao, C. (2009). Healthcare regulation as a tool for public accountability. *Med Health Care Philos, 12*(3), 257-264. doi:10.1007/s11019-008-9177-4

Nxumalo, N., Gilson, L., Goudge, J., Tsofa, B., Cleary, S., Barasa, E., & Molyneux, S. (2018). Accountability mechanisms and the value of relationships: Experiences of front-line managers at subnational level in Kenya and South Africa. *BMJ global health, 3*(4), e000842. doi:10.1136/bmjgh-2018-000842

Nxumalo, N. L. (2013). *Community health workers, community participation and community level inter-sectoral action: The challenges of implementing primary health care outreach services.* (Doctor of Philosophy). University of the Witwatersrand, Johannesburg.

O'Connell, K. J., Shaw, K. N., Ruddy, R. M., Mahajan, P. V., Lichenstein, R., Olsen, C. S., . . . OʼConnell, K. J. (2018). Incident Reporting to Improve Patient Safety: The Effects of Process Variance on Pediatric Patient Safety in the Emergency Department. *Pediatric Emergency Care, 34*(4), 237-242. doi:10.1097/PEC.0000000000001464

O'Hara, J. K., Armitage, G., Reynolds, C., Coulson, C., Thorp, L., Din, I., . . . Wright, J. (2017). How might health services capture patient-reported safety concerns in a hospital setting? An exploratory pilot study of three mechanisms. *BMJ Qual Saf, 26*(1), 42-53. doi:10.1136/bmjqs-2015-004260

O'Hara, J. K., Lawton, R. J., Armitage, G., Sheard, L., Marsh, C., Cocks, K., . . . Wright, J. (2016). The patient reporting and action for a safe environment (PRASE) intervention: a feasibility study. *BMC Health Serv Res, 16*(1), 676. doi:10.1186/s12913-016-1919-z

O'Shea, A., Boaz, A. L., & Chambers, M. (2019). A Hierarchy of Power: The Place of Patient and Public Involvement in Healthcare Service Development. *Frontiers in Sociology, 4*(38). doi:10.3389/fsoc.2019.00038

O’Meara, W. P., Tsofa, B., Molyneux, S., Goodman, C., & McKenzie, F. E. (2011). Community and facility-level engagement in planning and budgeting for the government health sector–A district perspective from Kenya. *Health Policy, 99*(3), 234-243.

Ocloo, J., & Matthews, R. (2016). From tokenism to empowerment: progressing patient and public involvement in healthcare improvement. *BMJ Qual Saf, 25*(8), 626-632. doi:10.1136/bmjqs-2015-004839

Ocloo, J. E., & Fulop, N. J. (2012). Developing a 'critical' approach to patient and public involvement in patient safety in the NHS: learning lessons from other parts of the public sector? *Health Expect, 15*(4), 424-432. doi:10.1111/j.1369-7625.2011.00695.x

Oguntunde, O., Surajo, I. M., Dauda, D. S., Salihu, A., Anas-Kolo, S., & Sinai, I. (2018). Overcoming barriers to access and utilization of maternal, newborn and child health services in northern Nigeria: an evaluation of facility health committees. *BMC Health Serv Res, 18*(1), 104. doi:10.1186/s12913-018-2902-7

Ogwang, S., Najjemba, R., Tumwesigye, N. M., & Orach, C. G. (2012). Community involvement in obstetric emergency management in rural areas: a case of Rukungiri district, Western Uganda. *BMC Pregnancy Childbirth, 12*, 20. doi:10.1186/1471-2393-12-20

Okafor, N. G., Doshi, P. B., Miller, S. K., McCarthy, J. J., Hoot, N. R., Darger, B. F., . . . Chathampally, Y. G. (2015). Voluntary medical incident reporting tool to improve physician reporting of medical errors in an emergency department. *West J Emerg Med, 16*(7), 1073-1078. doi:10.5811/westjem.2015.8.27390

Okeyo, I., Lehmann, U., & Schneider, H. (2020). The impact of differing frames on early stages of intersectoral collaboration: the case of the First 1000 Days Initiative in the Western Cape Province. *Health Research Policy and Systems, 18*(1), 3. doi:10.1186/s12961-019-0508-0

Olayo, R., Wafula, C., Aseyo, E., Loum, C., & Kaseje, D. (2014). A quasi-experimental assessment of the effectiveness of the community health strategy on health outcomes in Kenya. *BMC Health Services Research, 14 Suppl 1*, S3. doi:10.1186/1472-6963-14-s1-s3

Oliver, S., Armes, D. G., & Gyte, G. (2009). Public involvement in setting a national research agenda: A mixed methods evaluation. *The Patient: Patient-Centered Outcomes Research, 2*(3), 179-190. doi:10.2165/11314860-000000000-00000

Ortiz, J. P., Valentine, N. B., Gakidou, E., Tandon, A., Kawabata, K., Evans, D. B., & Murray, C. J. (2003). Inequality in responsiveness: Population surveys from 16 OECD countries. *Health Systems Performance Assessment: Debates, Methods and Empiricism*, 653-668.

Padarath, A., & Friedman, I. (2008). *The status of clinic committees in primary level public health sector facilities in South Africa*. Retrieved from Durban:

Pagatpatan, C. P., & Ward, P. R. (2017). Understanding the factors that make public participation effective in health policy and planning: a realist synthesis. *Australian Journal of Primary Health, 23*(6), 516-524. doi:10.1071/PY16129

Paina, L., Saracino, J., Bishai, J., & Sarriot, E. (2019). *Monitoring and evaluation of evolving social accountability efforts in health: a literature synthesis*. Retrieved from

Panda, B., Zodpey, S. P., & Thakur, H. P. (2016). Local self governance in health - a study of it's functioning in Odisha, India. *BMC Health Services Research, 16*, 15-27. doi:10.1186/s12913-016-1785-8

Park, Y., Kim, C. Y., You, M. S., Lee, K. S., & Park, E. (2014). Public participation in the process of local public health policy, using policy network analysis. *J Prev Med Public Health, 47*(6), 298-308. doi:10.3961/jpmph.14.029

Paschke, A., Dimancesco, D., Vian, T., Kohler, J. C., & Forte, G. (2018). Increasing transparency and accountability in national pharmaceutical systems. *Bull World Health Organ, 96*(11), 782-791. doi:10.2471/blt.17.206516

Patterson, M. E., & Pace, H. A. (2016). A cross-sectional analysis investigating organizational factors that influence near-miss error reporting among hospital pharmacists. *J Patient Saf, 12*(2), 114-117. doi:10.1097/pts.0000000000000125

Pearce, M. G. (2016). *Examining eye care in the South Pacific through a health systems strengthening lens.* (78). ProQuest Information & Learning, Retrieved from <http://ezproxy.uct.ac.za/login?url=https://search.ebscohost.com/login.aspx?direct=true&db=psyh&AN=2017-10864-264&site=ehost-live> Available from EBSCOhost psyh database.

Peltzer, K. (2009). Patient experiences and health system responsiveness in South Africa. *BMC Health Services Research, 9*, 117. doi:10.1186/1472-6963-9-117

Peltzer, K., & Phaswana-Mafuya, N. (2012). Patient experiences and health system responsiveness among older adults in South Africa. *Glob Health Action, 5*(1), 18545. doi:ARTN 18545

10.3402/gha.v5i0.18545

Penney, C. (2004). Understanding accountability in the Canadian health system. *Healthc Manage Forum, 17*(2), 9-15, 48-55. doi:10.1016/S0840-4704(10)60322-5

Penno, E., & Gauld, R. (2017). The role, costs and value for money of external consultancies in the health sector: A study of New Zealand's District Health Boards. *Health Policy, 121*(4), 458-467. doi:10.1016/j.healthpol.2017.02.005

Perera, W., Seneviratne, R., & Fernando, T. (2011). Development and validation of an instrument assessing Health System Responsiveness of family planning services in Sri Lanka. *South East Asia Journal of Public Health, 1*(1), 46-52.

Perera, W. L. S. P., Mwanri, L., de A Seneviratne, R., & Fernando, T. (2012). Health systems responsiveness and its correlates: Evidence from family planning service provision

in Sri Lanka. *WHO South-East Asia Journal of Public Health, 1*(4), 457-466.

Perrott, B. E. (2013). Including customers in health service design. *Health Mark Q, 30*(2), 114-127. doi:10.1080/07359683.2013.787882

Peters, D. H., Noor, A. A., Singh, L. P., Kakar, F. K., Hansen, P. M., & Burnham, G. (2007). A balanced scorecard for health services in Afghanistan. *Un cuadro de mando para los servicios de salud del Afganistán., 85*(2), 146-151. doi:10.2471/BLT.06.033746

Phaswana-Mafuya, N., Peltzer, K., Hoosain, E., & Maseko, B. (2017). Factors contributing to patients’ satisfaction with public health services in the Eastern Cape, South Africa. *23*, 59-68. Retrieved from <http://ezproxy.uct.ac.za/login?url=https://search.ebscohost.com/login.aspx?direct=true&db=awn&AN=ajpherd-154819&site=ehost-live>

Phiri, S. N. a., Fylkesnes, K., Ruano, A. L., & Moland, K. M. (2014). 'Born before arrival': user and provider perspectives on health facility childbirths in Kapiri Mposhi district, Zambia. *BMC Pregnancy & Childbirth, 14*(1), 1-10. doi:10.1186/1471-2393-14-323

Pichert, J. W., Moore, I. N., Karrass, J., Jay, J. S., Westlake, M. W., Catron, T. F., & Hickson, G. B. (2013). An intervention model that promotes accountability: peer messengers and patient/family complaints. *Jt Comm J Qual Patient Saf, 39*(10), 435-446.

Pieterse, P. (2019). Citizen feedback in a fragile setting: social accountability interventions in the primary healthcare sector in Sierra Leone. *Disasters, 43*, S132-S150. doi:10.1111/disa.12331

Polancich, S., Poe, T., Hackney, N., & Williamson, J. (2017). Using technology to support data analytics: a chief nursing officer accountability scorecard. *Journal for Healthcare Quality: Promoting Excellence in Healthcare, 39*(5), 315-320. doi:10.1097/JHQ.0000000000000109

Poles, G., Li, M., Siril, H., Mhalu, A., Hawkins, C., Kaaya, S., . . . Hirschhorn, L. R. (2014). Factors associated with different patterns of nonadherence to HIV care in Dar es Salaam, Tanzania. *Journal Of The International Association Of Providers Of AIDS Care, 13*(1), 78-84. doi:10.1177/1545109712467068

Pongsupap, Y., & Van Lerberghe, W. (2006a). Is motivation enough? Responsiveness, patient-centredness, medicalization and cost in family practice and conventional care settings in Thailand. *Hum Resour Health, 4*, 19-19. doi:10.1186/1478-4491-4-19

Pongsupap, Y., & Van Lerberghe, W. (2006b). Patient experience with self-styled family practices and conventional primary care in Thailand. *Asia Pacific Family Medicine, 5*, 4-11. Retrieved from <http://ezproxy.uct.ac.za/login?url=https://search.ebscohost.com/login.aspx?direct=true&db=aph&AN=31566648&site=ehost-live>

Post, D., Sanjay Agarwal, & Venugopa, V. (2014). *Rapid feedback: The role of community scorecards in improving service delivery*. Retrieved from <http://documents.worldbank.org/curated/en/462221468333561977/Rapid-feedback-the-role-of-community-scorecards-in-improving-service-delivery>

Potts, H., & Hunt, P. (2008). *Accountability and the right to the highest attainable standard of health*. Retrieved from

Prakash, G., & Singh, A. (2013). *Responsiveness in a district health system: The changing relationship of the state with its citizen*. Retrieved from

Pratt, B., & Hyder, A. A. (2015). Reinterpreting responsiveness for health systems research in low and middle-income countries. *Bioethics, 29*(6), 379-388. doi:10.1111/bioe.12138

Quinn, A., & Otteson, M. (2019). Strengthening the Voice of Those with Mental Health Issues: A Community Approach to Developing a Mental Health Identification System. *Social Work, 64*(3), 216-223. doi:10.1093/sw/swz017

Radford, A., Sheps, C. G., Pink, G., & Ricketts, T. (2007). A Comparative Performance Scorecard for Federally Funded Community Health Centers in North Carolina. *Journal of Healthcare Management, 52*(1), 20-31. doi:10.1097/00115514-200701000-00006

Rafiei, S., Irany Nasab, M., Montazerolfaraj, R., Sepaseh, F., Dehghani Tafti, A., & Askari, R. (2016). Managers’ Perspective toward Responsiveness in Non Clinical Services. *International Journal of Hospital Research, 5*(3), 93-97.

Rahman, M. H. U., Singh, A., & Madhavan, a. (2019). Disability-based disparity in outpatient health system responsiveness among the older adults in low- to upper-middle-income countries. *Health Policy and Planning*. doi:10.1093/heapol/czz013

Rajamani, S., Bieringer, A., Sowunmi, S., & Muscoplat, M. (2017). Stakeholder use and feedback on vaccination history and clinical decision support for immunizations offered by public health. *AMIA Annu Symp Proc, 2017*, 1450-1457.

Ramiro, L. S., Castillo, F. A., Tan-Torres, T., Torres, C. E., Tayag, J. G., Talampas, R. G., & Hawken, L. (2001). Community participation in local health boards in a decentralized setting: Cases from the Philippines. *Health Policy and Planning, 16 Suppl 2*, 61-69. doi:10.1093/heapol/16.suppl_2.61

Rashidian, A., Kavosi, Z., Majdzadeh, R., Pourreza, A., Pourmalek, F., Arab, M., & Mohammad, K. (2011). Assessing health system responsiveness: A household survey in 17th district of Tehran. *Iran Red Crescent Medical Journal, 13*(5), 302-308. Retrieved from <https://www.ncbi.nlm.nih.gov/pubmed/22737485>

Rawson, T. M., Castro‐Sánchez, E., Charani, E., Husson, F., Moore, L. S. P., Holmes, A. H., & Ahmad, R. (2018). Involving citizens in priority setting for public health research: Implementation in infection research. *Health Expectations: An International Journal of Public Participation in Health Care & Health Policy, 21*(1), 222-229. doi:10.1111/hex.12604

Reeleder, D., Goel, V., Singer, P. A., & Martin, D. K. (2006). Accountability agreements in Ontario hospitals: are they fair? *Journal of Public Administration Research and Theory, 18*(1), 161-175.

Reeve, C., Humphreys, J., Wakerman, J., Carroll, V., Carter, M., O'Brien, T., . . . Smith, B. (2015). Community participation in health service reform: the development of an innovative remote Aboriginal primary health-care service. *Aust J Prim Health, 21*(4), 409-416. doi:10.1071/py14073

Reid, P., Paine, S.-J., Curtis, E., Jones, R., Anderson, A., Willing, E., & Harwood, M. (2017). Achieving health equity in Aotearoa: strengthening responsiveness to Māori in health research. *New Zealand Medical Journal, 130*(1465), 96-103. Retrieved from <http://ezproxy.uct.ac.za/login?url=https://search.ebscohost.com/login.aspx?direct=true&db=cin20&AN=126158698&site=ehost-live>

Reinders, M. E., Ryan, B. L., Blankenstein, A. H., van der Horst, H. E., Stewart, M. A., & van Marwijk, H. W. (2011). The effect of patient feedback on physicians' consultation skills: a systematic review. *Acad Med, 86*(11), 1426-1436. doi:10.1097/ACM.0b013e3182312162

Renedo, A., & Marston, C. (2015). Spaces for citizen involvement in healthcare: An ethnographic study. *Sociology, 49*(3), 488-504. doi:10.1177/0038038514544208

Rezaei, S., Matin, B. K., Moradi, K., Bijan, B., Fallahi, M., Shokati, B., & Saeidi, H. (2016). Measurement of Quality of Educational Hospital Services by the SERVQUAL Model: The Iranian Patients' Perspective. *Electronic Physician, 8*(3), 2101-2106. doi:10.19082/2101

Rice, N., Robone, S., & Smith, P. (2011). Analysis of the validity of the vignette approach to correct for heterogeneity in reporting health system responsiveness. *European Journal of Health Economics, 12*(2), 141-162. doi:<https://link.springer.com/journal/volumesAndIssues/10198>

Rice, N., Robone, S., & Smith, P. C. (2008). *The measurement and comparison of health system responsiveness*. Retrieved from <https://pdfs.semanticscholar.org/c65d/92504b06480272faaf1fecfa8858208e54a9.pdf>

Rice, N., Robone, S., & Smith, P. C. (2010). International comparison of public sector performance: the use of anchoring vignettes to adjust self-reported data. *Comparaison internationale des performances du secteur public: l'utilisation des cas types pour ajuster les données d'auto-rapportage., 16*(1), 81-101. doi:10.1177/1356389009350127

Rice, N., Robone, S., & Smith, P. C. (2012). Vignettes and health systems responsiveness in cross-country comparative analyses. *Journal of the Royal Statistical Society: Series A (Statistics in Society), 175*(2), 337-369. doi:10.1111/j.1467-985X.2011.01021.x

Rifkin, S. B. (2014). Examining the links between community participation and health outcomes: A review of the literature. *Health Policy and Planning, 29*(Suppl 2), 98-106. doi:10.1093/heapol/czu076

Ringold, D., Holla, A., Koziol, M., & Srinivasan, S. (2012). *Citizens and service delivery: Assessing the use of social accountability approaches in human development*. Retrieved from Washington DC:

Rise, M. B., Eriksen, L., Grimstad, H., & Steinsbekk, A. (2012). The short-term effect on alliance and satisfaction of using patient feedback scales in mental health out-patient treatment. A randomised controlled trial. *BMC Health Serv Res, 12*, 348. doi:10.1186/1472-6963-12-348

Rise, M. B., Eriksen, L., Grimstad, H., & Steinsbekk, A. (2016). The long-term effect on mental health symptoms and patient activation of using patient feedback scales in mental health out-patient treatment. A randomised controlled trial. *Patient Educ Couns, 99*(1), 164-168. doi:10.1016/j.pec.2015.07.016

Rise, M. B., Solbjor, M., Lara, M. C., Westerlund, H., Grimstad, H., & Steinsbekk, A. (2013). Same description, different values. How service users and providers define patient and public involvement in health care. *Health Expect, 16*(3), 266-276. doi:10.1111/j.1369-7625.2011.00713.x

Ristea, A.-L., Stegaroiu, I., Ioan-Franc, V., & Dinu, V. (2009). Responsiveness of health systems: A barometer of the qualityof health services. *Quality Management in Services, XI*(26).

Robinson, V. A., Hunter, D., Shortt, S. E. D., Robinson, V. A., Hunter, D., & Shortt, S. E. D. (2003). Accountability in public health units: using a modified nominal group technique to develop a balanced scorecard for performance measurement. *Canadian Journal of Public Health, 94*(5), 391-396. Retrieved from <http://ezproxy.uct.ac.za/login?url=https://search.ebscohost.com/login.aspx?direct=true&db=cin20&AN=106761486&site=ehost-live>

Robone, S., Rice, N., & Smith, P. C. (2010). *Health systems’ responsiveness and its characteristics: A cross-country comparative analysis*. Retrieved from

Robone, S., Rice, N., & Smith, P. C. (2011). Health systems' responsiveness and its characteristics: A cross‐country comparative analysis. *Health Services Research, 46*(6pt2), 2079-2100. doi:10.1111/j.1475-6773.2011.01291.x

Rosenberg, S., & Rosen, A. (2012). Can mental health commissions really drive reform? Towards better resourcing, services, accountability and stakeholder engagement. *Australasian Psychiatry, 20*(3), 193-198. Retrieved from <https://journals.sagepub.com/doi/pdf/10.1177/1039856212447860>

Rossouw, L., & Smith, A. (2017). A comparable yardstick: Adjusting for education bias in South African health system responsiveness ratings. *Health Policy and Planning, 32*(ppl_3), iii67-iii74. Retrieved from <http://ezproxy.uct.ac.za/login?url=https://search.ebscohost.com/login.aspx?direct=true&db=awn&AN=29149309&site=ehost-live>

Rottger, J., Blumel, M., Engel, S., Grenz-Farenholtz, B., Fuchs, S., Linder, R., . . . Busse, R. (2015). Exploring health system responsiveness in ambulatory care and disease management and its relation to other dimensions of health system performance (RAC) - study design and methodology. *International Journal of Health Policy and Management, 4*(7), 431-437. doi:10.15171/ijhpm.2015.97

Rottger, J., Blumel, M., Fuchs, S., & Busse, R. (2014). Assessing the responsiveness of chronic disease care - is the World Health Organization's concept of health system responsiveness applicable? *Social Science & Medicine, 113*, 87-94. doi:10.1016/j.socscimed.2014.05.009

Röttger, J., Blümel, M., Köppen, J., & Busse, R. (2016). Forgone care among chronically ill patients in Germany-Results from a cross-sectional survey with 15,565 individuals. *Health Policy, 120*(2), 170-178. doi:10.1016/j.healthpol.2016.01.004

Röttger, J., Blümel, M., Linder, R., & Busse, R. (2017). Health system responsiveness and chronic disease care—What is the role of disease management programs? An analysis based on cross-sectional survey and administrative claims data. *Social Science & Medicine, 185*, 54-62. doi:10.1016/j.socscimed.2017.05.034

Roussos, S. T., & Fawcett, S. B. (2000). A review of collaborative partnerships as a strategy for improving community health. *Annual Review of Public Health, 21*(1), 369-402. doi:doi:10.1146/annurev.publhealth.21.1.369

Rowe, R., & Shepherd, M. (2002). Public participation in the new NHS: no closer to citizen control? *Social Policy & Administration, 36*(3), 275. Retrieved from <http://ezproxy.uct.ac.za/login?url=https://search.ebscohost.com/login.aspx?direct=true&db=sih&AN=6832259&site=ehost-live>

Roy, I. (2008). Civil society and good governance: (Re-) conceptualizing the interface. *World Development, 36*(4), 677-705. doi:10.1016/j.worlddev.2007.04.020

Rozenblum, R., Greaves, F., & Bates, D. W. (2017). The role of social media around patient experience and engagement. In *BMJ Qual Saf* (2017/04/22 ed., Vol. 26, pp. 845-848).

Rubrichi, S., Battistotti, A., & Quaglini, S. (2014). Patients' involvement in e-health services quality assessment: a system for the automatic interpretation of SMS-based patients' feedback. *J Biomed Inform, 51*, 41-48. doi:10.1016/j.jbi.2014.03.003

Saadat, M., Panah, A. D., Noroozi, F., & Alamdari, A. (2017). Evaluating relationship between the implementation of healthcare reform and patient satisfaction in health care centers of Yasuj. *Middle East Journal of Family Medicine*, 157-161. doi:10.5742/MEWFM.2017.93155

Sajjadi, F., Moradi-Lakeh, M., Nojomi, M., Baradaran, H. R., & Azizi, F. (2015). Health system responsiveness for outpatient care in people with diabetes Mellitus in Tehran. *Medical Journal Of The Islamic Republic Of Iran, 29*, 1-13. Retrieved from <http://ezproxy.uct.ac.za/login?url=https://search.ebscohost.com/login.aspx?direct=true&db=aph&AN=125165395&site=ehost-live>

Schaad, B., Bourquin, C., Panese, F., & Stiefel, F. (2019). How physicians make sense of their experience of being involved in hospital users' complaints and the associated mediation. *BMC Health Services Research, 19*(1), 1-8. doi:10.1186/s12913-019-3905-8

Schaaf, M., Chhabra, S., Flores, W., Feruglio, F., Dasgupta, J., & Ruano, A. L. (2018). Does information and communication technology add value to citizen-led accountability initiatives in health? Experiences from india and Guatemala. *Health Hum Rights, 20*(2), 169-184.

Schaaf, M., & Dasgupta, J. (2019). "Our fear is finished," but nothing changes: efforts of marginalized women to foment state accountability for maternal health care in a context of low state capacity. *BMC Public Health, 19*(1), 732-732. doi:10.1186/s12889-019-7028-2

Schaaf, M., Fox, J., Topp, S. M., Warthin, C., Freedman, L. P., Robinson, R. S., . . . Closser, S. (2018). Community health workers and accountability: reflections from an international "think-in". *Int J Equity Health, 17*(1), 66. doi:10.1186/s12939-018-0781-5

Schaaf, M., Topp, S. M., & Ngulube, M. (2017). From favours to entitlements: Community voice and action and health service quality in Zambia. *Health Policy and Planning, 32*(6), 847-859. doi:10.1093/heapol/czx024

Schnitzer, S., Kuhlmey, A., Adolph, H., Holzhausen, J., & Schenk, L. (2012). Complaints as indicators of health care shortcomings: which groups of patients are affected? *International Journal for Quality in Health Care, 24*(5), 476-482. doi:10.1093/intqhc/mzs036

Schwartz, R., Price, A., Deber, R. B., Manson, H., & Scott, F. (2014). Hopes and realities of public health accountability policies. *Healthc Policy, 10*(Spec issue), 79-89.

Sciamanna, C. N., Novak, S. P., Houston, T. K., Gramling, R., & Marcus, B. H. (2004). Visit satisfaction and tailored health behavior communications in primary care. *Am J Prev Med, 26*(5), 426-430. doi:10.1016/j.amepre.2004.02.003

Scott, D. A. H., & Grant, S. M. (2018). A meta‐ethnography of the facilitators and barriers to successful implementation of patient complaints processes in health‐care settings. *Health Expectations, 21*(2), 508-517. doi:10.1111/hex.12645

Scott, H., & Danel, I. (2016). Accountability for improving maternal and newborn health. *Best Pract Res Clin Obstet Gynaecol, 36*, 45-56. doi:10.1016/j.bpobgyn.2016.05.009

Scott, J., Heavey, E., Waring, J., Jones, D., & Dawson, P. (2016). Healthcare professional and patient codesign and validation of a mechanism for service users to feedback patient safety experiences following a care transfer: a qualitative study. *BMJ Open, 6*(7), e011222-e011222. doi:10.1136/bmjopen-2016-011222

Scott, K., Jessani, N., Qiu, M., & Bennett, S. (2018). Developing more participatory and accountable institutions for health: identifying health system research priorities for the Sustainable Development Goal-era. *Health Policy Plan, 33*(9), 975-987. doi:10.1093/heapol/czy079

Scuffham, P. A., Whitty, J. A., Taylor, M., & Saxby, R. C. (2010). Health system choice: A pilot discrete-choice experiment eliciting the preferences of British and Australian citizens. *Applied Health Economics and Health Policy, 8*(2), 89-97. doi:10.2165/11531170-000000000-00000

Serapioni, M., & Duxbury, N. (2014). Citizens' participation in the Italian health-care system: the experience of the Mixed Advisory Committees. *Health Expect, 17*(4), 488-499. doi:10.1111/j.1369-7625.2012.00775.x

Serapioni, M., & Matos, A. R. (2014). Citizen participation and discontent in three Southern European health systems. *Social Science & Medicine, 123*, 226-233. doi:10.1016/j.socscimed.2014.06.006

Serrate, P. C.-F., Lausanne, R. C., Jean-Claude, M. M., Espinosa, C. S., & Gonzalez, T. C. (2007). *Study on intersector practices in health in Cuba: Report to the pan American health organization, stage one*. Retrieved from Havanah:

Shaikh, M., Miraldo, M., & Renner, A.-T. (2018). Waiting time at health facilities and social class: Evidence from the Indian caste system. *PLoS One, 13*(10), 1-16. doi:10.1371/journal.pone.0205641

Sharma, A. E., & Grumbach, K. (2017). Engaging patients in primary care practice transformation: Theory, evidence and practice. *Family Practice, 34*(3), 262-267. Retrieved from <http://ezproxy.uct.ac.za/login?url=https://search.ebscohost.com/login.aspx?direct=true&db=psyh&AN=2017-29956-002&site=ehost-live>

anjana.sharma@ucsf.edu

Shaw, H., Rohde, P., & Stice, E. (2016). Participant feedback from peer-led, clinician-led, and internet-delivered eating disorder prevention interventions. *Int J Eat Disord, 49*(12), 1087-1092. doi:10.1002/eat.22605

Sheard, L., Marsh, C., O'Hara, J., Armitage, G., Wright, J., & Lawton, R. (2017). The Patient Feedback Response Framework - Understanding why UK hospital staff find it difficult to make improvements based on patient feedback: A qualitative study. *Soc Sci Med, 178*, 19-27. doi:10.1016/j.socscimed.2017.02.005

Shepherd, A., Sanders, C., Doyle, M., & Shaw, J. (2015). Using social media for support and feedback by mental health service users: Thematic analysis of a twitter conversation. *BMC Psychiatry, 15*. Retrieved from <http://ezproxy.uct.ac.za/login?url=https://search.ebscohost.com/login.aspx?direct=true&db=psyh&AN=2015-08679-001&site=ehost-live>

jennifer.j.shaw@manchester.ac.uk

michael.doyle@manchester.ac.uk

caroline.sanders@manchester.ac.uk

andrew.shepherd-4@postgrad.manchester.ac.uk

Shiyanbola, O. O., Smith, P. D., Mansukhani, S. G., & Huang, Y. M. (2016). Refining prescription warning labels using patient feedback: a qualitative study. *PLoS One, 11*(6), e0156881. doi:10.1371/journal.pone.0156881

Shrivastava, S. R., Shrivastava, P. S., & Ramasamy, J. (2013). Community monitoring. *Gateways: International Journal of Community Research & Engagement, 6*, 170-177. Retrieved from <http://ezproxy.uct.ac.za/login?url=https://search.ebscohost.com/login.aspx?direct=true&db=aph&AN=95846560&site=ehost-live>

Shukla, A., & Sinha, S. S. (2014). *Reclaiming public health through community-based monitoring: The case of Maharashtra, India*. Retrieved from

Simpson, E. L., & House, A. O. (2002). Involving users in the delivery and evaluation of mental health services: systematic review. *BMJ, 325*(7375), 1265. doi:10.1136/bmj.325.7375.1265

Siriwardena, A. N., & Gillam, S. (2014). Patient perspectives on quality. *Qual Prim Care, 22*(1), 11-15.

Sirven, N., Santos-Eggimann, B., & Spagnoli, J. (2008). *Comparability of health care responsiveness in Europe using anchoring vignettes from SHARE.* Retrieved from

Skeen, S., Kleintjes, S., Lund, C., Petersen, I., Bhana, A., & Flisher, A. J. (2010). 'Mental health is everybody's business': roles for an intersectoral approach in South Africa. *Int Rev Psychiatry, 22*(6), 611-623. doi:10.3109/09540261.2010.535510

Slowiak, J. M., & Huitema, B. E. (2015). Reducing pharmacy wait time to promote customer service: a follow-up study. *Qual Manag Health Care, 24*(1), 9-20. doi:10.1097/qmh.0000000000000045

Slutsky, J., Tumilty, E., Max, C., Lu, L., Tantivess, S., Hauegen, R. C., . . . Cubillos, L. (2016). Patterns of public participation: Opportunity structures and mobilization from a cross-national perspective. *J Health Organ Manag, 30*(5), 751-768. doi:10.1108/jhom-03-2016-0037

Smailhodzic, E., Hooijsma, W., Boonstra, A., & Langley, D. J. (2016). Social media use in healthcare: A systematic review of effects on patients and on their relationship with healthcare professionals. *BMC Health Serv Res, 16*, 442. doi:10.1186/s12913-016-1691-0

Smith, L. U. (2017). Community engagement framework for community assessment and improvement planning. *J Public Health Manag Pract, 23 Suppl 4 Suppl, Community Health Status Assessment*, S22-s28. doi:10.1097/phh.0000000000000600

Solbjor, M., Rise, M. B., Westerlund, H., & Steinsbekk, A. (2013). Patient participation in mental healthcare: when is it difficult? A qualitative study of users and providers in a mental health hospital in Norway. *Int J Soc Psychiatry, 59*(2), 107-113. doi:10.1177/0020764011423464

Solon, O., Woo, K., Stella, A. Q., Riti, S., Jhiedon, F., & John, W. P. (2009). A novel method for measuring health care system performance: experience from QIDS in the Philippines. *Health Policy & Planning, 24*(3), 167-167. doi:10.1093/heapol/czp003

Sorensen, R., & Iedema, R. (2008). Redefining accountability in health care: managing the plurality of medical interests. *Health:, 12*(1), 87-106.

Souliotis, K. (2015). Quality in healthcare and the contribution of patient and public involvement: talking the talk and walking the walk? *Health Expect, 18*(1), 1-2. doi:10.1111/hex.12321

Souliotis, K. (2016). Patient participation: Are we far from fulfilling the vision of patient‐centred care? *Health Expectations: An International Journal of Public Participation in Health Care & Health Policy, 19*(4), 787-789. doi:10.1111/hex.12483

South, J., & Phillips, G. (2014). Evaluating community engagement as part of the public health system. *J Epidemiol Community Health, 68*, 692-696. doi:10.1136/jech-2013-203742

Southwick, F. S., Cranley, N. M., & Hallisy, J. A. (2015). A patient-initiated voluntary online survey of adverse medical events: the perspective of 696 injured patients and families. *BMJ Quality & Safety, 24*(10), 620-629. doi:10.1136/bmjqs-2015-003980

Sri B, S., Sarojini, N., & Khanna, R. (2012). An investigation of maternal deaths following public protests in a tribal district of Madhya Pradesh, central India. *Reprod Health Matters, 20*(39), 11-20. doi:10.1016/s0968-8080(12)39599-2

Srivastava, A., Bhattacharyya, S., Gautham, M., Schellenberg, J., & Avan, B. I. (2016). Linkages between public and non-government sectors in healthcare: A case study from Uttar Pradesh, India. *Global Public Health, 11*(10), 1216-1230. Retrieved from <http://ezproxy.uct.ac.za/login?url=https://search.ebscohost.com/login.aspx?direct=true&db=awn&AN=26947898&site=ehost-live>

Srivastava, A., Gope, R., Nair, N., Rath, S., Rath, S., Sinha, R., . . . Bhattacharyya, S. (2016). Are village health sanitation and nutrition committees fulfilling their roles for decentralised health planning and action? A mixed methods study from rural eastern India. *BMC Public Health, 16*(1), 59. doi:10.1186/s12889-016-2699-4

Stepurko, T., Pavlova, M., & Groot, W. (2016). Overall satisfaction of health care users with the quality of and access to health care services: a cross-sectional study in six Central and Eastern European countries. *BMC Health Services Research, 16*, 1-13. doi:10.1186/s12913-016-1585-1

Stevenson, D., & Sinclair, N. (2018). Complaints about hospice care in the United States, 2005–2015. *Journal of Palliative Medicine, 21*(11), 1580-1587. doi:10.1089/jpm.2018.0125

Stewardson, A. J., Sax, H., Gayet-Ageron, A., Touveneau, S., Longtin, Y., Zingg, W., & Pittet, D. (2016). Enhanced performance feedback and patient participation to improve hand hygiene compliance of health-care workers in the setting of established multimodal promotion: a single-centre, cluster randomised controlled trial. *Lancet Infect Dis, 16*(12), 1345-1355. doi:10.1016/s1473-3099(16)30256-0

Street, J., Duszynski, K., Krawczyk, S., & Braunack-Mayer, A. (2014). The use of citizens' juries in health policy decision-making: A systematic review. *Social Science & Medicine, 109*, 1-9. doi:10.1016/j.socscimed.2014.03.005

Subhedar, N. V., & Parry, H. A. (2010). Critical incident reporting in neonatal practice. *Arch Dis Child Fetal Neonatal Ed, 95*(5), F378-382. doi:10.1136/adc.2008.137869

Tabrizi, J. S., Farahbakhsh, M., Bazargani, H. S., Saadati, M., Golestani, M., & Zakeri, A. (2018). Health services utilization and responsiveness: a comparison of slum and Non-slum regions in Tabriz, Iran. *Medical Science, 22*, 577-582.

Tancred, T., Mandu, R., Hanson, C., Okuga, M., Manzi, F., Peterson, S., . . . The EQUIP Study Team. (2014). How people-centred health systems can reach the grassroots: experiences implementing community-level quality improvement in rural Tanzania and Uganda. *Health Policy and Planning*. doi:10.1093/heapol/czu070

Taylor, D. M., Wolfe, R. S., & Cameron, P. A. (2004). Analysis of complaints lodged by patients attending Victorian hospitals, 1997–2001. *MJA, 181*(1), 31-35. Retrieved from <Go to ISI>://WOS:000222819900013

Taylor, S., Abbott, S., & Hardy, S. (2012). The INFORM project: a service user-led research endeavor. *Arch Psychiatr Nurs, 26*(6), 448-456. doi:10.1016/j.apnu.2012.02.005

Teklehaimanot, H. D., Teklehaimanot, A., Tedella, A. A., & Abdella, M. (2016). Use of balanced scorecard methodology for performance measurement of the health extension program in Ethiopia. *American Journal of Tropical Medicine and Hygiene, 94*(5), 1157-1169. Retrieved from <http://ezproxy.uct.ac.za/login?url=https://search.ebscohost.com/login.aspx?direct=true&db=awn&AN=26928842&site=ehost-live>

Teno, J. M., Montgomery, R., Valuck, T., Corrigan, J., Meier, D. E., Kelley, A., . . . Engelberg, R. (2018). Accountability for community-based programs for the seriously ill. *Journal of Palliative Medicine, 21*(S2), S-81-S-87.

Thornton, R. D., Nurse, N., Snavely, L., Hackett-Zahler, S., Frank, K., & DiTomasso, R. A. (2017). Influences on patient satisfaction in healthcare centers: a semi-quantitative study over 5 years. *BMC Health Serv Res, 17*(1), 361. doi:10.1186/s12913-017-2307-z

Thurston, W. E., MacKean, G., Vollman, A., Casebeer, A., Weber, M., Maloff, B., & Bader, J. (2005). Public participation in regional health policy: A theoretical framework. *Health Policy, 73*(3), 237-252. doi:10.1016/j.healthpol.2004.11.013

Thurston, W. E., Robinson Vollman, A., Meadows, L. M., & Rutherford, E. (2005). Public participation for women's health: strange bedfellows or partners in a cause? *Health Care for Women International, 26*(5), 398-421. Retrieved from <http://ezproxy.uct.ac.za/login?url=https://search.ebscohost.com/login.aspx?direct=true&db=cin20&AN=106505320&site=ehost-live>

Tierney, E., McEvoy, R., Hannigan, A., & MacFarlane, A. E. (2018). Implementing community participation via interdisciplinary teams in primary care: An Irish case study in practice. *Health Expectations: An International Journal of Public Participation in Health Care & Health Policy, 21*(6), 990-1001. doi:10.1111/hex.12692

Tighe, C. M., Woloshynowych, M., Brown, R., Wears, B., & Vincent, C. (2006). Incident reporting in one UK accident and emergency department. *Accid Emerg Nurs, 14*(1), 27-37. doi:10.1016/j.aaen.2005.10.001

Tille, F., Röttger, J., Gibis, B., Busse, R., Kuhlmey, A., & Schnitzer, S. (2019). Patients’ perceptions of health system responsiveness in ambulatory care in Germany. *Patient education and counseling, 102*(1), 162-171. doi:10.1016/j.pec.2018.08.020

Topp, S. M., Black, J., Morrow, M., Chipukuma, J. M., & Van Damme, W. (2015). The impact of human immunodeficiency virus (HIV) service scale-up on mechanisms of accountability in Zambian primary health centres: A case-based health systems analysis. *BMC Health Services Research, 15*(67). doi:10.1186/s12913-015-0703-9

Topp, S. M., Edelman, A., & Taylor, S. (2018). "We are everything to everyone": A systematic review of factors influencing the accountability relationships of Aboriginal and Torres Strait Islander health workers (AHWs) in the Australian health system. *International Journal for Equity in Health, 17*(1), 67-67. doi:10.1186/s12939-018-0779-z

Topp, S. M., Sharma, A., Chileshe, C., Magwende, G., Henostroza, G., & Moonga, C. N. (2018). The health system accountability impact of prison health committees in Zambia. *International Journal for Equity in Health, 17*(1), 74. doi:10.1186/s12939-018-0783-3

Torabipour, A., Gharacheh, L., Lorestani, L., & Salehi, R. (2017). Comparison of responsiveness level in Iranian public and private physiotherapy clinics: A cross-sectional multi-center study. *Materia Socio-Medica, 29*(3), 172-175. doi:10.5455/msm.2017.29.172-175

Tremblay, D., Roberge, D., & Berbiche, D. (2015). Determinants of patient-reported experience of cancer services responsiveness. *BMC Health Services Research, 15*(1), 425.

Trevena, L., Shepherd, H. L., Bonner, C., Jansen, J., Cust, A. E., Leask, J., . . . Hoffmann, T. (2017). Shared decision making in Australia in 2017. *Z Evid Fortbild Qual Gesundhwes, 123-124*, 17-20. doi:10.1016/j.zefq.2017.05.011

Tripathy, J. P., Aggarwal, A. K., Patro, B. K., & Verma, H. (2015). Process evaluation of community monitoring under national health mission at Chandigarh, union territory: Methodology and challenges. *J Family Med Prim Care, 4*(4), 539-545. doi:10.4103/2249-4863.174282

Tritter, J. Q. (2011). Public and patient participation in health care and health policy in the United Kingdom. *Health Expectations, 14*(2), 220-223. doi:10.1111/j.1369-7625.2011.00697.x

Turney, B. W., & Reynard, J. M. (2014). Obtaining patient feedback in an outpatient lithotripsy service is facilitated by use of a touch-screen tablet (iPad) survey. *Urolithiasis, 42*(4), 317-321. doi:10.1007/s00240-014-0662-3

Tursunbayeva, A., Franco, M., & Pagliari, C. (2017). Use of social media for e-Government in the public health sector: A systematic review of published studies. *Government Information Quarterly, 34*(2), 270-282. doi:10.1016/j.giq.2017.04.001

Twiddy, M., Czoski Murray, C. J., Mason, S. J., Meads, D., Wright, J. M., Mitchell, E. D., & Minton, J. (2018). A qualitative study of patients' feedback about Outpatient Parenteral Antimicrobial Therapy (OPAT) services in Northern England: implications for service improvement. *BMJ Open, 8*(1), e019099. doi:10.1136/bmjopen-2017-019099

Ughasoro, M. D., Okanya, O. C., Uzochukwu, B. S. C., & Onwujekwe, O. E. (2017). An exploratory study of patients’ perceptions of responsiveness of tertiary health-care services in Southeast Nigeria: A hospital-based cross-sectional study. *Nigerian Journal of Clinical Practice, 20*, 267-273. Retrieved from <http://ezproxy.uct.ac.za/login?url=https://search.ebscohost.com/login.aspx?direct=true&db=awn&AN=njcp-152440&site=ehost-live>

Ugurluoglu, O., & Celik, Y. (2006). How responsive Turkish health care system is to its citizens: the views of hospital managers. *Journal of Medical Systems, 30*(6), 421-428. doi:10.1007/s10916-005-9006-8

Unknown. (2003). Technical consultation on concepts and methods for measuring the responsiveness of health systems. In C. J. L. Murray & D. B. Evans (Eds.), *Health systems performance assessment: debates, methods and empiricism.* (pp. 115-123). Geneva: World Health Organization.

USAID. (2001). *The citizens monitoring and feedback mechanism: A guide for LGUs in installing a participatory monitoring and evaluation system*. Retrieved from

Ustun, T. B., Chatterji, S., Villanueva, M. V., Bendib, L., Celik, C., Sadana, R., . . . Murray, C. J. L. (2003). *WHO Multi-Country Survey Study on Health and Responsiveness 2000–2001*. Retrieved from Geneva:

Uzochukwu, B., Mbachu, C., Okeke, C., Onwujekwe, E., Molyneux, S., & Gilson, L. (2018). Accountability mechanisms for implementing a health financing option: the case of the basic health care provision fund (BHCPF) in Nigeria. *International Journal for Equity in Health, 17*(1), N.PAG-N.PAG. doi:10.1186/s12939-018-0807-z

Uzochukwu, B. S. C. (2011). Trust, accountability and performance in health facility committees in Orumba south local government area, Anambra state, Nigeria. Retrieved from <http://www.crehs.lshtm.ac.uk/nigeria_accountability_hr_14june2011.pdf>

Uzwiak, B. A., & Curran, S. (2016). Gendering the burden of care: health reform and the paradox of community participation in Western Belize. *Med Anthropol Q, 30*(1), 100-121. doi:10.1111/maq.12195

Valentine, N., Darby, C., & Bonsel, G. J. (2008). Which aspects of non-clinical quality of care are most important? Results from WHO's general population surveys of 'health systems responsiveness' in 41 countries. *Social Science & Medicine, 66*, 1939-1950.

Valentine, N., de Silva, A., & Murray, C. J. L. (2000). Estimating responsiveness level and distribution for 191 Countries: Methods and results. In *GPE Discussion Paper Series: No.22*. Geneva: World Health Organization.

Valentine, N., Verdes-Tennant, E., & Bonsel, G. (2015). Health systems' responsiveness and reporting behaviour: Multilevel analysis of the influence of individual-level factors in 64 countries. *Social Science & Medicine, 138*, 152-160. doi:10.1016/j.socscimed.2015.04.022

Valentine, N. B., & Bonsel, G. J. (2016). Exploring models for the roles of health systems' responsiveness and social determinants in explaining universal health coverage and health outcomes. *Glob Health Action, 9*(29329). doi:10.3402/gha.v9.29329

Valentine, N. B., Bonsel, G. J., & Murray, C. J. L. (2007). Measuring quality of health care from the user's perspective in 41 countries: Psychometric properties of WHO's questions on health systems responsiveness. *Quality of Life Research: An International Journal of Quality of Life Aspects of Treatment, Care & Rehabilitation, 16*(7), 1107-1125. doi:10.1007/s11136-007-9189-1

Valentine, N. B., de Silva, A., Kawabata, K., Darby, C., Murray, C. J. L., & Evans, D. B. (2003). Health system responsiveness: Concepts, domains and measurement. In C. J. L. Murray & D. B. Evans (Eds.), *Health Systems Performance Assessment: Debates, Methods and Empiricism*. Geneva: World Health Organisation.

Valentine, N. B., Lavallée, R., Bao, L., Bonsel, G. J., & Murray, C. J. L. (2003). Classical psychometric assessment of the responsiveness instrument in the who multi-country survey study on health and responsiveness 2000-2001. In *Health systems performance assessment: debates, methods and empiricism.* (pp. 597-629). Geneva: World Health Organization.

Valentine, N. B., Ortiz, J. P., Tandon, A., Kawabata, K., Evans, D. B., & Murray, C. J. L. (2003). Patient experiences with health services: population surveys from 16 OECD countries. In C. J. L. Murray & D. B. Evans (Eds.), *Health systems performance assessment: debates, methods and empiricism.* (pp. 643-652). Geneva: World Health Organization.

Valentine, N. B., Salomon, J. A., Murray, C., Evans, D., Murray, C., & Evans, D. (2003). Weights for responsiveness domains: analysis of country variation in 65 national sample surveys. In C. J. L. Murray & D. B. Evans (Eds.), *Health systems performance assessment: debates, methods and empiricism.* (pp. 631-652). Geneva: World Health Organization.

Van Belle, S., & Mayhew, S. H. (2016a). Public accountability needs to be enforced –a case study of the governance arrangements and accountability practices in a rural health district in Ghana. *BMC Health Services Research, 16*(568), 1-14.

Van Belle, S., & Mayhew, S. H. (2016b). What can we learn on public accountability from non-health disciplines: A meta-narrative review. *BMJ Open, 6*, e010425. doi:10.1136/bmjopen-2015-010425

van der Kooy, J., Birnie, E., Valentine, N. B., de Graaf, J. P., Denktas, S., Steegers, E. A. P., & Bonsel, G. J. (2017). Quality of perinatal care services from the user's perspective: a Dutch study applies the World Health Organization's responsiveness concept. *BMC Pregnancy & Childbirth, 17*, 1-11. doi:10.1186/s12884-017-1464-8

van der Kooy, J., Valentine, N. B., Birnie, E., Vujkovic, M., de Graaf, J. P., Denktas, S., . . . Bonsel, G. J. (2014). Validity of a questionnaire measuring the World Health Organization concept of health system responsiveness with respect to perinatal services in the dutch obstetric care system. *BMC Health Services Research, 14*, 622. doi:10.1186/s12913-014-0622-1

Van der Stuyft, P., & Unger, J. P. (2000). Editorial: Improving the performance of health systems: the World Health Report as go-between for scientific evidence and ideological discourse. *Tropical Medicine & International Health, 5*(10), 675-677. doi:10.1046/j.1365-3156.2000.00635.x

van Oenen, F. J., Schipper, S., Van, R., Schoevers, R., Visch, I., Peen, J., & Dekker, J. (2016). Feedback-informed treatment in emergency psychiatry; a randomised controlled trial. *BMC Psychiatry, 16*. doi:10.1186/s12888-016-0811-z

van Velthoven, M. H., Atherton, H., & Powell, J. (2018). A cross sectional survey of the UK public to understand use of online ratings and reviews of health services. *Patient Educ Couns, 101*(9), 1690-1696. doi:10.1016/j.pec.2018.04.001

Vanzetta, M., Vellone, E., Dal Molin, A., Rocco, G., De Marinis, M. G., & Rosaria, A. (2014). Communication with the public in the health-care system: a descriptive study of the use of social media in local health authorities and public hospitals in Italy. *Ann Ist Super Sanita, 50*(2), 163-170. doi:10.4415/ann_14_02_10

Veillard, J., Huynh, T., Ardal, S., Kadandale, S., Klazinga, N. S., & Brown, A. D. (2010). Making health system performance measurement useful to policy makers: aligning strategies, measurement and local health system accountability in Ontario. *Healthcare Policy, 5*(3), 49-65. Retrieved from <http://ezproxy.uct.ac.za/login?url=https://search.ebscohost.com/login.aspx?direct=true&db=cin20&AN=105147611&site=ehost-live>

Velonis, A. J., Molnar, A., Lee-Foon, N., Rahim, A., Boushel, M., & O'Campo, P. (2018). "One program that could improve health in this neighbourhood is ____?" using concept mapping to engage communities as part of a health and human services needs assessment. *BMC Health Serv Res, 18*(1), 150. doi:10.1186/s12913-018-2936-x

Vincent, C. A. (2004). Analysis of clinical incidents: a window on the system not a search for root causes. In *Qual Saf Health Care* (2004/08/04 ed., Vol. 13, pp. 242-243).

Vrbnjak, D., Denieffe, S., O’Gorman, C., & Pajnkihar, M. (2016). Barriers to reporting medication errors and near misses among nurses: A systematic review. *Int J Nurs Stud, 63*, 162-178. doi:10.1016/j.ijnurstu.2016.08.019

Wada, K., Kakuma, R., Hoshi, K., Sato, Y., Hasegawa, T., & Satoh, T. (2011). Factors associated with preferences for health system goals in Japan: A pilot study of the World Health Survey. *Asia-Pacific Journal of Public Health, 23*(5), 721-729. doi:10.1177/1010539511418101

Wagner, C., Merten, H., Zwaan, L., Lubberding, S., Timmermans, D., & Smits, M. (2016). Unit-based incident reporting and root cause analysis: variation at three hospital unit types. *BMJ Open, 6*(6), e011277. doi:10.1136/bmjopen-2016-011277

Wahedi, K., Flores, W., Beiersmann, C., Bozorgmehr, K., & Jahn, A. (2018). Using information communication technology to identify deficits in rural health care: a mixed-methods evaluation from Guatemala. *Glob Health Action, 11*(1), 1-1. doi:10.1080/16549716.2018.1455347

Waldman, L., Theobald, S., & Morgan, R. (2018). *Key considerations for accountability and gender in health systems in low-and Middle-income countries*.

Wallack, L. (2000). *The Role of Mass Media in Creating Social Capital: A New Direction for Public Health*.

Wallcraft, J. (2012). What has been learned from joint working between mental health professionals, patients and users of psychiatric services, their families and friends? *Curr Opin Psychiatry, 25*(4), 317-321. doi:10.1097/YCO.0b013e32835462d0

Ward, V., Pinkney, L., & Fry, G. (2016). Developing a framework for gathering and using service user experiences to improve integrated health and social care: the SUFFICE framework. *BMC Res Notes, 9*(1), 437. doi:10.1186/s13104-016-2230-0

Weale, A. (2016). Between consensus and contestation. *Journal Of Health Organization And Management, 30*(5), 786-795. doi:10.1108/jhom-03-2016-0040

Weir, E., d'Entremont, N., Stalker, S., Kurji, K., & Robinson, V. (2009). Applying the balanced scorecard to local public health performance measurement: deliberations and decisions. *BMC Public Health, 9*, 127. doi:10.1186/1471-2458-9-127

Whitney, J., Easter, A., & Tchanturia, K. (2008). Service users' feedback on cognitive training in the treatment of anorexia nervosa: a qualitative study. *International Journal of Eating Disorders, 41*(6), 542-550. Retrieved from <http://ezproxy.uct.ac.za/login?url=https://search.ebscohost.com/login.aspx?direct=true&db=cin20&AN=105551403&site=ehost-live>

Williamson, L. (2014). Patient and citizen participation in health: the need for improved ethical support. *Am J Bioeth, 14*(6), 4-16. doi:10.1080/15265161.2014.900139

Willig, J. H., Krawitz, M., Panjamapirom, A., Ray, M. N., Nevin, C. R., English, T. M., . . . Berner, E. S. (2013). Closing the feedback loop: an interactive voice response system to provide follow-up and feedback in primary care settings. *J Med Syst, 37*(2), 9905. doi:10.1007/s10916-012-9905-4

Wilson, L. J., Yepuri, J. N., & Moses, R. E. (2016). The advantages and challenges of measuring patient experience in outpatient clinical practice. Part 3: patient satisfaction and your practice. In *American Journal of Gastroenterology* (2016/04/06 ed., Vol. 111, pp. 757-759).

Witvliet, M. I., Stronks, K., Kunst, A. E., Mahapatra, T., & Arah, O. A. (2015). Linking health system responsiveness to political rights and civil liberties: a multilevel analysis using data from 44 countries. *International Journal of Health Services, 45*(4), 622-642. doi:10.1177/0020731415585980

Woollard, R., Buchman, S., Meili, R., Strasser, R., Alexander, I., & Goel, R. (2016). Social accountability at the meso level: Into the community. *Canadian Family Physician, 62*(7), 538-540.

World Bank. (2011). *Accountability in public services in South Africa*. Retrieved from Washington, DC: <http://siteresources.worldbank.org/INTSOUTHAFRICA/Resources/Accountability_in_Public_Services_in_Africa.pdf>

World Health Organization. (2000a). *Overall health systems achievement for 191 countries* Retrieved from

World Health Organization. (2000b). *The world health report 2000: Health systems: Improving performance* (924156198X). Retrieved from Geneva: <https://www.who.int/whr/2000/en/>

World Health Organization. (2003). *Health system responsiveness survey results: Equitable, humane, patient-centred treatment by health systems, or not? Sample report*. Retrieved from Geneva, Switzerland: <https://www.who.int/responsiveness/KIS%20Report.pdf>

World Health Organization. (2005). *The health systems responsiveness analytical guidelines for surveys in the Multi-country Survey Study*. Retrieved from Geneva:

World Health Organization. (2011). *Intersectoral action on health: A path for policy-makers to implement effective and sustainable action on health*. Retrieved from Kobe:

World Health Organization. (2015). Accountability for women's and children's health: 2015 progress report.

World Health Organization. (2016). *Framework on integrated people-centred health services*. Retrieved from Geneva: <http://www.who.int/servicedeliverysafety/areas/people-centred-care/framework/en/>

Wouters, E., Heunis, C., van Rensburg, D., & Meulemans, H. (2008). Patient satisfaction with antiretroviral services at primary health-care facilities in the Free State, South Africa–a two-year study using four waves of cross-sectional data. *BMC Health Services Research, 8*(1), 1. Retrieved from <http://download.springer.com/static/pdf/478/art%253A10.1186%252F1472-6963-8-1.pdf?originUrl=http%3A%2F%2Fhttp%3A%2F%2Fbmchealthservres.biomedcentral.com%2Farticle%2F10.1186%2F1472-6963-8-1&token2=exp=1462886636~acl=%2Fstatic%2Fpdf%2F478%2Fart%25253A10.1186%25252F1472-6963-8-1.pdf*~hmac=c5c997bf84d60e52f62f6cfdcc147790171eaf0087f1180e967300f6a9f3baae>

Wright, C., Davey, A., Elmore, N., Carter, M., Mounce, L., Wilson, E., . . . Campbell, J. (2017). Patients' use and views of real-time feedback technology in general practice. *Health Expect, 20*(3), 419-433. doi:10.1111/hex.12469

Wu, X., Li, S., Xu, N., Wu, D., & Zhang, X. (2019). Establishing a balanced scorecard measurement system for integrated care organizations in China. *International Journal of Health Planning & Management, 34*(2), 672-692. doi:10.1002/hpm.2726

Yakob, B., & Ncama, B. P. (2016a). Client satisfaction: correlates and implications for improving HIV/AIDS treatment and care services in southern Ethiopia. *International Health (1876-3413), 8*(4), 292-298. doi:10.1093/inthealth/Ihw008

Yakob, B., & Ncama, B. P. (2016b). Correlates of strengthening lessons from HIV/AIDS treatment and care services in Ethiopia perceived access and implications for health system. *PLoS One, 11*(8), 1-19. doi:10.1371/journal.pone.0161553

Yakob, B., & Ncama, B. P. (2017). Measuring health system responsiveness at facility level in Ethiopia: performance, correlates and implications. *BMC Health Services Research, 17*(1), 263. doi:10.1186/s12913-017-2224-1

Yamin, A. E., & Lander, F. (2015). Implementing a circle of accountability: A proposed framework for judiciaries and other actors in enforcing health-related rights. *Journal of Human Rights, 14*(3), 312-331. doi:10.1080/14754835.2015.1056874

Yasobant, S., Saxena, D., Trivedi, M., Gaurav, K., Patel, S., & Patel, M. (2016). Advocacy for a responsive health system to control diabetes: Learning from western Indian state Gujarat, India. *International Journal of Medical Science and Public Health, 5*(11), 1-5. doi:10.5455/ijmsph.2016.06042016447

Yassoub, R., Hashimi, S., Awada, S., & El‐Jardali, F. (2014). Responsiveness of Lebanon's primary healthcare centers to non‐communicable diseases and related healthcare needs. *The International Journal of Health Planning and Management, 29*(4), 407-421.

Zakkar, M. (2019). Patient experience: determinants and manifestations. *International Journal of Health Governance, 24*(2), 143-154. doi:10.1108/IJHG-09-2018-0046

Zalmanovitch, Y., & Vashdi, D. R. (2015). The relationship between socio-economic factors and responsiveness gaps in primary, preventative and health promotion services. *Health Expectations, 18*(6), 2638-2650. doi:10.1111/hex.12238

Ziewitz, M. (2017). Experience in action: Moderating care in web-based patient feedback. *Soc Sci Med, 175*, 99-108. doi:10.1016/j.socscimed.2016.12.028
